# CDS-localized m^6^A drives co-translational RNA decay to relieve biotic and abiotic endoplasmic reticulum stresses

**DOI:** 10.1038/s41477-026-02299-4

**Published:** 2026-05-08

**Authors:** Songxiao Zhong, Taerin Oh, Xindi Li, Jinming Zhang, Suk Won Choi, Chuyun Gao, Xu Peng, Juan Dong, Chuan He, Xiuren Zhang

**Affiliations:** 1 Department of Biochemistry and Biophysics, Texas A&M University, College Station, TX 77843, USA.; 2 Department of Chemistry, The University of Chicago, Chicago, IL 60637, USA.; 3 Howard Hughes Medical Institute, The University of Chicago, Chicago, IL 60637, USA.; 4 Xenohelix Research Institute, Incheon 21984, Republic of Korea; 5 CAS Center for Excellence in Molecular Plant Sciences, Institute of Plant Physiology and Ecology, Chinese Academy of Sciences, Shanghai 200032, China; 6 Department of Medical Physiology, Vashisht College of Medicine, Texas A&M University, Bryan, TX 77807, USA; 7 Institute for Biophysical Dynamics, The University of Chicago, Chicago, IL 60637, USA.; 8 Department of Biochemistry and Molecular Biology, The University of Chicago, Chicago, IL 60637, USA.; 9 Former: Department of Biochemistry and Biophysics, Texas A&M University, College Station, TX 77843, USA.; 10 Institute of Emerging Agricultural Technology, Shenzhen University of Advanced Technology, Shenzhen, Guangdong, 518107, China.; 11 Faculty of Synthetic Biology, Shenzhen University of Advanced Technology, Shenzhen, Guangdong, 518107, China.; 12 These authors contributed equally.

## Abstract

Endoplasmic reticulum (ER) mitigates stress typically through unfolded protein response (UPR) and ER-associated degradation (ERAD) pathways, yet post-transcriptional regulation of ER stress remains poorly defined. N6-methyladenosine (m^6^A) modification, predominantly enriched near stop codons, can govern mRNA fates via P-bodies or stress granules in stress conditions. m^6^A also occurs within coding sequences (CDS-m^6^A), but its role remains unappreciated in plants. Here we demonstrate that m^6^A ablation sensitizes Arabidopsis ER stress despite normal UPR and ERAD activities. Mechanistically, CDS-m^6^A colocalizes with ribosome stalling sites and directs co-translational RNA decay (CTRD). Under stress, activation of m^6^A-triggered CTRD accelerates clearance of ER-engaged transcripts, thereby alleviating translational overload. During Geminivirus infection, which increases translational demand on ER, m^6^A-triggered CTRD also targets viral RNAs, restricting their accumulation, translation and disease progression. Thus, CDS-m^6^A functions as a pivotal regulator of ER-linked RNA surveillance, establishing an organelle-specific mechanism that integrates RNA stability, protein homeostasis, and antiviral defense.

## Introduction

The *N*^*6*^-methyladenosine (m^6^A) represents the most prevalent internal RNA modification in eukaryotes, dynamically regulating transcript stability, splicing, and translation to orchestrate adaptive responses to environmental stressors^1–4^. In plants, m^6^A is deposited by a multicomponent writer complex, including the methyltransferases A (MTA) and MTB, which target adenosine residues within consensus motifs such as RRACH (R = A and G; H = A, C and U) and the plant-specific URUAY (Y = C and U) sequences^5^. These consensus sequences are typically detected in the regions close to stop codons, whereas m^6^A is less frequently observed in the CDS due to the conflict between m^6^A writing machinery and exon junction complex (EJC)^6,7^. However, a substantial proportion of m^6^A modification is located within the CDS, particularly in proximity to the start codon^8,9^ where the modification been implicated in promoting RNA decay in mammals^10–13^. Nevertheless, precise function of CDS localized m^6^A remains to be elucidated in plants.

The m^6^A mark is interpreted by reader proteins, such as YTH domain-containing evolutionarily conserved C-terminal region (ECT) family members, which mediate context-dependent determination of RNA fates^14–16^. Moreover, m^6^A erasers including alpha-ketoglutarate-dependent dioxygenase homolog 9B and 10B (ALKBH9B/10B) mediate the dynamic regulation of m^6^A during plant floral transition and adaptation to abiotic stresses^17–19^. Recent advances highlight that m^6^A, especially 3ÙTR localized ones (3ÙTR-m^6^A), is critical in adapting plants to both biotic and abiotic stresses, by shaping the transcriptome in response to environmental cues^20^. For instance, the ECT readers sequester salicylic acid (SA)-induced m^6^A-modified mRNAs into cytoplasmic biomolecular condensates, accelerating their degradation and fine-tuning SA-mediated immunity^21–23^. Similarly, m^6^A modulates abscisic acid (ABA) signaling, which is a central axis of abiotic stress resilience, and dictates transcript behaviors and fates through processing-bodies (P-bodies) and stress granules (SGs)^17,19,24–27^. In biotic interactions such as viral and pathogen infection, m^6^A serves as a dynamic regulatory interface, with its deposition on responsive transcripts being closely associated with changes in their accumulation abundance^23,28–31^. Interestingly, 3ÙTR-m^6^A exerts a biphasic regulatory effect on RNA stability, simultaneously stabilizing some transcripts to support normal development while promoting the decay of others to regulate adaptive stress responses^8,23–25,30,32–34^. Although extensive research has explored the functional significance of 3ÙTR-m^6^A, a proportion of m^6^A sites occur within CDS, and whether they play any specific roles in biological processes remains unexplored. In addition, the mechanisms by which different compartmentations coordinate m^6^A-mediated regulation of RNA in plants remain largely enigmatic, highlighting a pressing need to define contextual determinants and subcellular effectors.

The endoplasmic reticulum (ER) serves as an eukaryotic nexus for protein synthesis, folding, and quality control, and is pivotal for proteostasis amid environmental fluctuations^35^. Recent studies have expanded the scope of ER translation to encompass approximately one-third of cellular proteome including both membrane-bound and cytosolic fractions, underscoring its regulatory significance in maintaining translational homeostasis^36,37^. This process is highly sensitive to a spectrum of disturbances: biotic challenges such as viral and bacterial infection and abiotic stresses including hormonal imbalance or thermal fluctuations, can provoke ER stress and trigger adaptive recalibration of the proteome^38–40^. In eukaryotes, ER stress activates the unfolded protein response (UPR) which typically increase the production of molecular chaperones and help proteins fold properly whereas removing misfolded proteins promptly. The UPR is executed via some primary sensors. For example, the transcription factors exemplified by basic leucine zipper protein (bZIP) 28 and 17, and activating transcription factor 6 (ATF6) can reprogram the transcription of chaperone genes. On the other hand, inositol requiring enzyme 1(IRE1)mediates RNA cleavage and cytosolic splicing; protein kinase RNA-like ER kinase (PERK) attenuates global translation initiation^37,41^. Moreover, the ER-associated degradation (ERAD) pathway can clear misfolded or aberrant proteins^42^. However, plants lack PERK orthologs and thus were previously thought to rely primarily on UPR and ERAD, which may increase the burden on the pathways, suggesting the involvement of additional yet-to-be characterized mechanisms involved in ER stress resolution^43^.

Mechanisms coupling translation with RNA decay have recently gained prominence in molecular research since the initial observation in yeast, where RPF-associated quality control plays a central role in mRNA nonsense-mediated decay (NMD)^44^. This mechanism, when ribosome encounters a premature stop codon in an mRNA, ensures timely termination and degradation of aberrant RNAs, thereby protecting cells from stress-induced damage, and preventing physiological disorders in humans^45^. In plants, translation-coupled RNA decay has been identified and can be stimulated by heat stress^46,47^, and subsequent degradome sequencing has identified significant ribosome protected fragments (RPFs)^48–51^, thus revealing a pervasive presence of the phenomena. Yet, the features that govern RNA susceptibility to degradation during translation remain largely undetermined.

In this study, we interrogated possible roles of m^6^A in the ER stress response and antiviral defense in *Arabidopsis*, with particular focus on the contribution of CDS-m^6^A to RNA decay. Chemical induction of ER stress leads to upregulation of m^6^A methyltransferases, and this response is essential for optimal adaptation, as evidenced by the reduced resilience of m^6^A writer mutants to the stress. Notably, loss of m^6^A does not impair the activation of the UPR or ERAD pathways. Interestingly, although CDS-m^6^A is less abundant than that in the 3' UTR, the transcripts bearing CDS-m^6^A exhibit significantly shorter half-lives. Furthermore, the transcripts bearing both CDS-m^6^A and overlapping ribosome footprints are particularly prone to rapid degradation. In lines with this, degradome sequencing confirms the accumulation of RNA decay intermediates at these sites. Under ER stress, the spatial convergence of m^6^A and ribosome occupancy accelerates co-translational RNA decay at the ER membrane. In the *mta* plants lacking m^6^A, these transcripts are instead stabilized and aberrantly accumulate. Moreover, a viral infection triggers a robust ER stress response, and methylated viral transcripts are similarly targeted for m^6^A-mediated degradation at the ER. Abrogation of the m^6^A-dependent decay in the mutants leads to excessive accumulation of viral RNA. Collectively, these findings identify CDS-m^6^A as a critical regulator of RNA decay at ER, essential for resolving both abiotic and biotic ER stress responses in plants.

## Results

### RNA m^6^A writers contribute to ER stress response

To explore a potential role of m^6^A in the plant ER stress response, we examined public transcriptomic datasets for *Arabidopsis thaliana*. A few abiotic and biotic exposures are known to trigger ER stress response, including heat^52,53^, abscisic acid (ABA)^54^, salicylic acid (SA)^55^, bacterial flagellin^56^, and pathogens^57^. Intriguingly, under these conditions, the expression of m^6^A writer components was consistently induced, reminiscent of the pattern of the ER stress marker *BiP3* (Extended Data Fig. 1a–f). We next performed a pan-expression analysis across more than 1,000 independent *Arabidopsis* transcriptomes^58^, an underlying rationale for the analysis is that functionally related genes typically evolve to exhibit correlated expression patterns^24,25,59^. Strikingly, the m^6^A writer genes displayed clearly positive co-expression with key regulators of the UPR and ERAD pathways, whereas no such correlation was observed with housekeeping genes such as *ACTIN2* and *UBQ10* (Extended Data Fig. 1g). These results suggested that m^6^A might have some genetic association with ER stress pathways.

We next experimentally examined if the m^6^A pathway responded to the ER stress. We treated plant seedlings with tunicamycin (Tm), a standard inhibitor of N-linked glycosylation that promotes the accumulation of misfolded proteins in the ER lumen^60^. Indeed, the transcript abundance of m^6^A writer genes was significantly increased in treatment with Tm vs DMSO control (Fig. 1a). Consistently, the protein levels of the writers also accumulated upon Tm exposure (Fig. 1b, Extended Data Fig. 2a). We previously reported that the MTB subunit of the writer complex contains a large intrinsically disordered N-terminal domain and tends to form insoluble aggregates^59^. On the other hand, the prevailing model is that ER stress is accompanied by widespread protein misfolding^37^. Then we asked whether the accumulated m^6^A writer proteins corresponded to an active or inactive form. Cellular fractionation of the samples collected after stress induction, followed by immunoblotting, revealed that the soluble portion of both MTA and MTB rose pronouncedly (Extended Data Fig. 2a,b). This increase might reflect sustained UPR activation, which employs chaperones to facilitate protein folding. Thus, the increased soluble fraction of writers is reasonably assumed to represent its catalytically competent conformation. In agreement, global m^6^A level on mRNA was significantly elevated following the treatment with Tm versus DMSO control (Fig. 1c).

To determine whether m^6^A contributed to the ER stress response, we subjected seedlings to prolonged Tm-induced ER stress. In wild-type (WT, Col-0) seedlings, chronic exposure resulted in developmental defects, including reduced root length and plant stature (Fig. 1d,e). At a higher dose (60 ng/mL), these effects were exacerbated, with additional cotyledon yellowing (Fig. 1d,e). We found that *mta* and *mtb* mutants, where m^6^A levels are impaired, exhibited hypersensitivity even at a lower dose (30 ng/mL), as they displayed significantly shortened roots and more pronounced growth inhibition compared to Col-0 (Fig. 1d–f). At a dose of 60 ng/mL, the mutants showed further reductions in root length and stature, and increased cotyledon yellowing (Fig. 1d–f). Cotyledon yellowing reflects ER stress that has surpassed the cell tolerance; therefore, we classified seedlings into three phenotypic categories based on cotyledon coloration: type I (fully green), type II (partially yellow), and type III (fully yellow) (Extended Data Fig. 2d). While most Col-0 seedlings fell into type I, exhibiting green cotyledons and robust root growth, m^6^A writer mutants predominantly displayed type III phenotypes, with smaller size and widespread cotyledon yellowing; a subset showed type II (Fig. 1e,g). Conversely, transgenic lines overexpressing *MTA* and *MTB*^59^ exhibited similar tolerance with Col-0, mirroring WT root lengths and morphologies at Tm doses of both 30 ng/mL and 60 ng/mL (Extended Data Fig. 2e,f). Phenotypic distributions in these lines were also comparable to WT (Extended Data Fig. 2g), indicating that MTA and MTB might function in a m^6^A dependent manner at ER stress response. These results demonstrate that m^6^A writer mutants are hypersensitive to ER stress compared to Col-0.

### m^6^A facilitates ER stress responses independently of canonical UPR and ERAD pathways

To further clarify whether m^6^A writers were implicated in the ER stress response, we examined the impact of m^6^A writer mutations on the expression of ER stress response machinery. Interestingly, a global differential expression analysis revealed modest accumulation of genes that are associated with the UPR branch of the ER stress pathway in *mta* (Extended Data Fig. 3a). Further, gene set enrichment analysis (GSEA) indicated an overall upregulation of the ER response pathway in *mta* (Fig. 1h). These results aligned with the observation that upon the treatment with under low-dose of Tm, a chronic ER stress, the plant UPR system exhibited a moderate yet sustained activation state (Extended Data Fig. 3b).

We next dissected the ER stress response pathways in *mta* and *mtb* mutants by subjecting seedlings to short-term treatment with Tm. Immunoblot analysis showed that the writer proteins had no visible accumulation in the mutants upon Tm exposure (Extended Data Fig. 3c). While, in the writer mutants, the transcripts of the ER stress marker *BiP3*, and *bZIP28*-driven *ERdj3B* accumulated, indicating that Tm induced the activation of UPR in the ER stress responses as normally as in Col-0 (Fig. 1i,j). We also monitored the splicing efficiency of *bZIP60* transcript (*sbZIP60),* for which, the generation of a shorter isoform would indicate IRE1 activation and can be efficiently detected by the indicated primer set^61^ (Extended Data Fig. 3d). Treatment with Tm significantly induced splicing efficiency with the accumulation of shorter isoform, indicative of intact sensor signaling in both WT and the mutants (Fig. 1h). Notably, the expression of core ERAD components was increased in plants treated with Tm (Extended Data Fig. 3e). Intriguingly, the expression of UPR markers and ERAD genes was already mildly upregulated in *mta* and *mtb vs* Col-0, prior to treatments with Tm (Fig. 1i,j, Extended Data Fig. 3e). The accumulation of these transcripts in the mutants further increased and reached to the levels of Col-0 upon the Tm exposure. Since the expression of UPR markers and ERAD genes started from a higher baseline in the mutants, the fold-change appeared to be relatively smaller despite of eventually achieving a level equivalent to that of Col-0 (Fig. 1i–h, Extended Data Fig. 3c,d). In parallel, we employed treatment with dithiothreitol (DTT), which disrupts disulfide bonds and induces strong reductive stress, thereby triggering protein misfolding and activating UPR and ERAD pathways^62^. Similar to Tm, treatment with DTT could increase the accumulation of transcripts and proteins of *MTA* and *MTB* as well as the total m^6^A level on mRNAs (Fig. 1b,c, Extended Data Fig. 3f). Furthermore, treatment with DTT could also cause upregulation of downstream UPR and ERAD and associated marker genes in *mta* and *mtb*, attaining levels equivalent to those in WT plants (Extended Data Fig. 3g–i).

We then examined whether additional components of the m^6^A machinery, including readers and erasers, were similarly linked to the ER stress response. The triple mutant *ect2 ect3 ect4* of cytosolic m^6^A readers, whose members are highly expressed and act in a partially redundant manner, exhibited a pronounced hypersensitivity to Tm, manifested as a severe inhibition of root growth (Extended Data Fig. 4a,b). In contrast, disruption of the m^6^A demethylases ALKBH9B and ALKBH10B caused increased tolerance to Tm, with *alkbh9b* displaying the more prominent phenotype (Extended Data Fig. 4c,d). Despite these divergent growth responses, the induction levels of the canonical ER stress markers *BiP3* and s*bZIP60* in these mutants were comparable to those observed in WT plants (Extended Data Fig. 4e,f). These results indicate that neither the UPR nor the ERAD pathway is overtly compromised in m^6^A pathway mutants relative to Col-0. Instead, our data suggest that m^6^A modification positively contributes to ER stress adaption.

### CDS-m^6^A modulates mRNA stability

Given that m^6^A regulates mRNA stability and that RNA degradation can be coupled to translation in eukaryotes^1^, we hypothesized that m^6^A might contribute to ER stress response via co-translationally regulating decay of mRNAs on ER. To test this, we first analyzed MeRIP-seq datasets^59^ to characterize the genome-wide distribution of m^6^A across mRNA regions. We categorized m^6^A sites according to their localizations into 5′ UTR-, CDS-, and 3ÙTR-m^6^A, identifying 957, 3,638, and 12,627 loci, respectively (Supplementary table. 1). The predominant distribution of the m^6^A modification in the 3′ UTR likely results from exclusion of the m^6^A writer machinery by exon junction complex (EJC); and this pattern is highly conserved among eukaryotes^6,7^. Notably, the number of m^6^A-modified transcripts considerably exceeds the count of upregulated genes in *mta* mutants (approximately 6,000)^59^, suggesting that m^6^A sites do not exert uniform regulatory effects. Indeed, transcript abundance varied according to regions harboring m^6^A modification: mRNAs with CDS-m^6^A exhibited the lowest steady-state levels, followed by the ones with 3′ UTR-m^6^A, while the transcripts with 5′ UTR-m^6^A showed the highest abundance (Fig. 2a). Intriguingly, this pattern was reversed in *mta*, where the transcripts that originally harbor CDS-m^6^A now displayed the greatest increase upon m^6^A loss; in contrast, the transcripts with 5′ UTR- and 3′ UTR-m^6^A were much less affected this time (Fig. 2b). Importantly, these differences did not result from the m^6^A modification levels, rather, they were essentially determined by the localizations of m^6^A sites in the transcripts. For instance, CDS-m^6^A exhibited intermediate enrichment - significantly lower than that observed in the 3′ UTR yet comparable to the 5′ UTR, thus, the CDS-m^6^A seemingly conferred its distinct regulatory impact on transcript abundance, compared to the m^6^A locations in either 5’ UTR or 3’ UTR (Fig. 2c).

We next explored how CDS-m^6^A influenced transcript levels. Given the pronounced accumulation of the m^6^A-harbored transcripts in *mta*, we assessed transcriptional output in response to treatments with Tm via polymerase II (Pol II) chromatin immunoprecipitation followed by qPCR (ChIP-qPCR). This analysis revealed no significant changes in transcription at the selected genes, with or without m^6^A in the CDS, in Tm-treated samples compared to mock samples (Extended Data Fig. 5a). We then evaluated relationship between m^6^A distribution and RNA stability using two independent metrics: the alpha index^63^ (representing degradation rate) and RNA half-life (t1/2)^64^, both derived from young seedlings. For ease of comparison, we plotted -log_2_(alpha index) and log_2_(t_1/2_), where both higher values denote greater stability. The transcripts with m^6^A in the CDS consistently exhibited significantly lower stability across both datasets than those with m^6^A in 5′ UTR- or 3′ UTR (Fig. 2d, Extended Data Fig. 5b). Notably, within CDS, the proximity of m^6^A modifications to the start codon is inversely correlated with RNA stability; in other words, transcripts with CDS-m^6^A sites located closer the start codon were associated with significantly reduced stability (Fig. 2f, Extended Data Fig. 5c). This positional dependency of mRNA stability was unique to CDS-localized m^6^A; as neither the distance of 5′ UTR-m^6^A from the start codon nor that of 3′ UTR-m^6^A from the stop codon showed significant correlations with RNA stability (Fig. 2e–g, Extended Data Fig. 5d–h). In contrast, RNA abundance and stability showed a consistent correlation for all three m^6^A positional classes, despite the degrees varied across the classes (Fig. 2h–j, Extended Data Fig. 5i–k). These results suggest that m^6^A distribution within the CDS region may play a critical role in modulating transcript stability.

### CDS-m^6^A and ribosome protected fragments overlapping transcripts are unstable

Given that the CDS is densely occupied by ribosomes, we next investigated whether CDS-m^6^A associates with ribosome dynamics. To do this, we utilized Translating Ribosome Affinity Purification sequencing dataset (TRAP-seq)^63^, which enriches ribosome-bound and polyadenine tailed mRNAs and provides a high-resolution map of ribosome occupancy, while capturing both translating transcripts and co-translationally degraded RNA substrates. TRAP-seq identified 46,984 potential ribosome-enriched peaks (Supplementary table. 2), with most of peaks mapping to CDS (37,234) and near stop codon regions (5,200), respectively (Extended Fig. 6a), closely mirroring distributions observed via ribosome profiling (Ribo-seq)^65^. To assess ribosome stalling, we analyzed RPFs to known m^6^A sites and observed overlaps of 0, 1,054, and 3,304 of ribosome peaks within the near start codon (comparing to 5` UTR-m^6^A), coding sequence (comparing to CDS-m^6^A), and near stop codon regions (comparing to 3` UTR-m^6^A), respectively (Extended Fig. 6b,c). Further characterization of the spatial relationship between RPFs and m^6^A sites within the CDS revealed a non-uniform distribution (Fig. 2k, bottom). Interestingly, these overlaps displayed a periodic pattern centered around m^6^A sites on the transcripts (Fig. 2k, top), reminiscent of the ~three-nucleotide phasing pattern characteristic of mRNA decay intermediates within coding regions as detected by degradome sequencing^49^.

Next, we examined the relationship between RNA stability and the overlap of RPFs and m^6^A signals. Among transcripts with overlapping peaks, transcripts bearing m^6^A in the CDS (referring to RPF-CDS-m^6^A) exhibited a stronger destabilization pattern compared to those with m^6^A in 3′ UTR (Fig. 2l, Extended Data Fig. 6d). In contrast, for transcripts without RPF overlap, mRNAs with CDS-m^6^A did not exhibit this destabilization pattern relative to those with m^6^A in 3′ UTR (Fig. 2m, Extended Data Fig. 6e). Moreover, increased RPF enrichment at CDS-m^6^A overlapping sites was associated with greater transcript instability (Fig. 2n, Extended Data Fig. 6f). Furthermore, ribosome stalling positively correlated with CDS-m^6^A stoichiometry, but not with 5ÙTR- and 3ÙTR-m^6^A, coinciding with increased ribosome occupancy at these sites (Fig. 2o, Extended Data Fig. 6g,h). Finally, among the RPF-overlapping sites, CDS-m^6^A distribution, unlike 3′ UTR-m^6^A, showed significant correlation with instability (Fig. 2p, Extended Data Fig. 6i–k). Further motif analysis of the above-mentioned overlapping regions identified enrichment of both canonical DRACH and plant-specific URUAY sequences^66^ (Extended Data Fig. 6j), implying a conserved and potentially broad interaction between m^6^A modification and ribosome dynamics across plant and animal systems. Collectively, these findings indicate that CDS-m^6^A modulates RNA stability through mechanisms that might closely tie to translation dynamics.

### CDS-m^6^A promotes co-translational RNA decay

To obtain a comprehensive understanding of CDS-m^6^A associated RNA cleavage and co-translational decay, we performed an integrative analysis combining Degradome-seq^67^ (Supplementary table. 3) and PARE-seq datasets^68^ (Supplementary table. 4). Both approaches capture RNA decay intermediates characterized by a 5′ monophosphate generated at the cleavage sites, which were selectively ligated to an RNA adapter^69,70^. We first examined how many transcripts underwent degradation events that initiated within coding sequences. For this purpose, we required the first 5′-end read of a transcript to map within the CDS and be located more than 17 nucleotides upstream of the stop codon. Applying these criteria, we identified 12,639 and 11,716 candidate transcripts from the Degradome-seq and PARE-seq datasets, respectively (Fig. 3a, Extended Data Fig. 7a). Strikingly, transcripts harboring RPF-CDS-m^6^A sites were markedly enriched among these candidates, showing substantially higher odds ratios compared with transcripts carrying non-RPF-overlapping CDS-m^6^A sites (Fig. 3a, Extended Data Fig. 7a)

To further assess the contribution of CDS-m^6^A to CTRD, we defined a Transcript Degradation Index (TDI) as the ratio of transcript abundance in the degradome to that in the RNA-seq (Extended Data Fig. 7b), with higher TDI values indicating a greater propensity to accumulate decay intermediates. Transcripts bearing CDS-m^6^A and exhibiting high TDI values showed more pronounced upregulation in *mta* vs WT plants (Fig.3b, Extended Data Fig. 7c). Notably, this trend was even stronger for transcripts containing RPF-CDS-m^6^A sites (Fig.3c, Extended Data Fig. 7d). Moreover, degradation initiation sites of these transcripts were preferentially enriched in the vicinity of m^6^A sites, particularly on the 5′ side (Fig.3d, Extended Data Fig. 7e), as illustrated by *AT1G09580*, *AT2G34730*, and other related genes (Fig. 3e; Extended Data Fig. 7f). In contrast, *AT5G61270* carried both 5′ UTR- and 3′ UTR-m^6^A yet displayed remarkable stability of transcripts, indicating that CDS-localized m^6^A is effective at promoting mRNA decay.

To further determine the role of translation in CDS-m^6^A mediating RNA decay, we analyzed GMUCT-seq datasets, another 5′-monophosphated decay intermediate capturing analysis, which were generated following the treatment with translation inhibitor cycloheximide (CHX)^49,71^. Prior to CHX application, transcripts bearing CDS-m^6^A were significantly more abundant in the degradome than those carrying 3′ UTR-m^6^A (Fig.3e). In contrast, after treatment with CHX, this relationship was reversed, with CDS-m^6^A–containing transcripts displaying even lower abundance than their 3′ UTR-m^6^A counterparts (Fig. 3e). Moreover, transcripts with RPF-CDS-m^6^A sites showed the highest abundance in the control condition but, upon treatment with CHX, dropped to levels comparable to those of 3′ UTR-m^6^A–bearing transcripts (Fig. 3f). Notably, gene ontology (GO) analysis of transcripts with RPF-CDS-m^6^A revealed a strong enrichment for pathways involved in RNA synthesis and protein transport, indicating preferential targeting of these functional categories for decay (Fig. 3g). Collectively, these results establish a tight association between CDS-m^6^A modification and CTRD.

### CDS-m^6^A promotes RNA decay at the ER

Having observed that CDS-m^6^A promotes mRNA decay, we next asked whether this mechanism contributes to RNA clearance at the ER. To test this, we examined mRNA accumulation on ER following loss of m^6^A. We isolated mRNA from subcellular fractions of Col-0 seedlings by using ultracentrifugation through a sucrose gradient to separate cytosolic and microsomal fractions, the latter being enriched for rough ER (Fig. 4a). RNA gel electrophoresis revealed clear enrichment of 28S and 18S ribosomal RNAs in the microsomal fraction without chloroplast rRNAs, validating successful rough ER isolation (Fig. 4b). Additionally, western blot analysis confirmed the specificity of the fractionations: the cytosolic marker UGPase was predominantly detected in the cytosolic fraction with minimal presence in the microsomal fraction, while the ER luminal protein BiP and the ribosomal protein RPL13 were substantially enriched in the microsomal fraction (Fig. 4c, Extended Data Fig. 8a).

We then assessed RNA content in ER-associated and cytosolic fractions. We found that ER-associated RNAs are methylated, but they exhibited lower m^6^A levels compared to their cytosolic counterparts in WT plants, while this modification was largely absent in the *mta* (Fig. 4g). We further performed MeRIP-qPCR and validated that ER-associated RNAs retained detectable m^6^A marks in Col-0, albeit at lower levels than their cytosolic counterparts (Fig. 4h). Meanwhile, these m^6^A-bearing transcripts displayed substantially reduced distributional disparities between cytosolic and microsomal fractions in *mta* vs Col-0, despite that the m^6^A modification was largely decreased in *mta* (Extended Data Fig. 9a). Specifically, in WT plants, the m^6^A-bearing RNAs were predominantly localized to the cytosolic fraction, whereas in *mta*, the corresponding transcripts showed remarkable enrichment on the microsomal fraction, accumulating 1.8- to 3-fold higher than that in wild type (Fig. 4i).

Interestingly, treatment with Tm increased total m^6^A levels in WT plants (Fig. 1c), with a pronounced shift toward ER-associated fractions (Fig. 4g,h). The increase in m^6^A modification corresponded with a reduction in RNA abundance on the ER (Fig. 4i). Corroborating the CDS-m^6^A accumulation, ribosome exhibited increased stalling near m^6^A loci (referring to loci 2) relative to a distal, non-overlapping site (referring to loci 1) (Fig. 4j, Extended Data Fig. 9b). In contrast, *mta* showed neither increase in m^6^A levels following treatment with Tm (Fig. 4g, Extended Data Fig. 9a) nor sustained RNA accumulation on the ER (Fig. 4i), with ribosome enrichment pattern unchanged (Fig. 4j). To further assess the relationship between ribosome enrichment and RNA stability, we treated seedlings with actinomycin D to block new RNA synthesis and measured half-lives of ER-associated transcripts (Fig. 4k–m). In Col-0, transcripts with RPF-CDS-m^6^A exhibited shorter half-lives compared to the control *AT5G61270* ((Fig. 4n). In contrast, this degradation pattern was significantly attenuated in *mta* (Fig. 4k–n).

Notably, parallel treatment with dithiothreitol (DTT) yielded similar results: Col-0 showed elevated CDS-m^6^A and ribosome overlaps alongside decreased RNA retention on ER, whereas *mta* displayed reduced m^6^A and ribosome signals and persistent RNA accumulation on ER (Extended Data Fig. 7e–h, 8d–i). Under co-treatment with actinomycin D, Col-0 exhibited an increase in RNA degradation rate, while *mta* showed only a marginal change (Extended Data Fig. 9g–i).

To further examine this process, we generated a semi-in vivo system expressing three kinds of transcripts that differ exclusively in the distribution of m^6^A sites: a reporter containing CDS-m^6^A-only (transcript #1), a reporter harboring m^6^A in both the CDS and the 3′UTR (transcript #2), and a mutant variant in which the CDS-m^6^A site was eliminated while the 3′UTR-m^6^A was preserved (transcript #3) (Fig. 4o, Supplementary table. 5). Microsomal fractions were isolated from these lines, and RNA decay intermediates were captured by 5′-adapter ligation followed by RT-PCR validation (Extended Data Fig. 10a). Under a basal conditions, the two reporters containing CDS-m^6^A (transcripts #1 and #2) accumulated comparable levels of decay intermediates, indicating that the presence of 3′UTR-m^6^A contributes minimally to this pathway (Fig. 4p,q). By contrast, abrogation of the CDS-m^6^A site resulted in a pronounced stabilization of the transcript, as reflected by the markedly reduced accumulation of intermediates in transcript #3 relative to transcript #2 (Fig. 4p,q). Upon treatment with Tm, the production of decay intermediates was further enhanced for transcripts #1 and #2, whereas transcript #3 remained largely refractory to induction (Fig. 4p,q).

Taken together, these results suggest an inverse correlation between m^6^A deposition and mRNA retention on the ER in stress conditions.

### m^6^A facilitates the biotic induced ER stress response

Given that as viral infections overload plant ER with the translation of exogenous proteins, we investigated whether CDS-m^6^A–mediated RNA decay also targeted viral transcripts during infection of Geminivirus, a major plant pathogen responsible for severe worldwide crop losses^72^. RNA-seq analysis revealed strong activation of ER stress response pathways in Col-0 plants (Fig. 5a). We also found increased expression of m^6^A writers’ transcripts and proteins, accompanied by elevated global RNA methylation in infected plants (Extended Data Fig. 10a–d). To examine ER-specific effects, we purified microsomal fractions from infected plants, and found increased RNA methylation in microsomal fraction (Extended Data Fig. 10d), indicating that Geminivirus infection also activates m^6^A pathway.

We next asked whether virus transcripts on ER are methylated. Since the highly compact and overlapping coding sequences of Geminivirus genome do not enable clear resolution of m^6^A via MeRIP sequencing, we designed gene-specific primers to resolve individual viral transcripts (Fig. 5b). MeRIP-PCR with full length RNA revealed clear CDS-m^6^A modifications on *AC1* and *AC2*, two early genes, and on the late genes, *BC1* and *BV1* (Fig. 5c). Be noted: the early genes are expressed predominantly during the initial stages of infection and function to promote the expression of the late genes^72^. *AC3* and *AV1*, which are embedded within AC1, were also enriched in the immunoprecipitate and therefore classified as potentially methylated transcripts (Fig. 5b,c, Extended Data Fig. 10e). We next examined the subcellular distribution of methylated viral RNAs in cytosolic and microsomal fractions. Methylated viral RNAs were detected in both compartments, and their levels were slightly lower in the microsomal fraction comparing to those in the cytosolic fraction (Fig. 5d). Intriguingly, Geminivirus early and late genes display distinct distribution patterns. In Col-0, early genes (*AC1* and *AC2*)—but not late genes (*BC1* and *BV1*)—showed lower abundance in the microsomal fraction compared to the cytosol (Fig. 5e). In contrast, in *mta*, viral RNA methylation was significant decreased, accompanied by marked accumulation of viral transcripts in both microsomal and cytosolic fractions (Fig. 5d,e). These results indicates that the transcripts of virus early genes might be regulated by m^6^A in plants.

To assess whether viral RNA undergoes translation-dependent degradation, we purified microsomal fraction and analyzed uncapped intermediates of viral RNA — a hallmark of 5′ to 3′ decay^48,49,67^ (Extended Data Fig. 10f). In Col-0, uncapped viral RNAs were detected via first-round RT-PCR, with amplicons corresponding primarily to *AC1*, *AC2*, *AC3*, and *AV1*, but not to the late genes, *BC1* and *BV1*, nor to unmethylated gene *AC4* (Fig. 5f, Extended Data Fig. 10g). In contrast, degradation intermediates of most viral transcripts were largely absent in *mta* (Fig. 5f). To further verify these PCR products, we performed a second round of nested PCR (Extended Data Fig. 10f bottom). Indeed, the assay validated the gene-specific bands as no satellite amplification was observed (Extended Data Fig. 10h). These results suggest that m^6^A promotes co-translational degradation of viral transcripts to suppress viral replication in Col-0 plants. Predicted from this model was that the m^6^A writer mutants would exhibit increased susceptibility to the virus infection compared to Col-0. Indeed, *mta* plants showed markedly elevated viral RNA accumulation and developed more severe disease symptoms than Col-0, characterized by pronounced leaf curling and extensive chlorosis (Fig. 5g–i). Thus, these findings demonstrate that plants employ CDS-m^6^A-mediated co-translational degradation of virus early genes to clear translational burden, thereby mitigating ER stress and enhancing plant immunity against virus infection.

## Discussion

Here, we identified CDS-m^6^A as a pivotal regulator of RNA stability and ER stress adaptation in *Arabidopsis thaliana*, with broad implications for antiviral defense. By facilitating co-translational RNA degradation on the ER, CDS-m^6^A offers a rapid and efficient mechanism to relieve translational stress, acting in parallel with the canonical UPR and ERAD pathways. These findings advance our understanding of epitranscriptomic regulation in plants and unveil a novel, organelle-specific role for m^6^A in modulating RNA fate under stress conditions. This work opens new avenues for exploring m^6^A-mediated regulatory mechanisms in stress tolerance and pathogen resistance.

Our study resolves a longstanding uncertainty regarding the biological significance of CDS-m^6^A modification. Previous work has reported dynamic levels of m^6^A along transcript bodies under stress conditions^20^, such as photodamage^73^, salt^25,74,75^, cold^76–78^, drought^24,79^, heat^19,26^, nutrient deficiency^80^, and virus and pathogen infection^17,23,27,30,81^, with pronounced increases in the CDS region. However, it has remained unclear whether these changes reflect functionally meaningful accumulation of CDS-m^6^A or merely statistical artifacts, limiting a comprehensive understanding of m^6^A’s role. Here, our findings fill this gap by demonstrating that CDS-m^6^A is functionally essential for ER-associated RNA degradation, facilitating stress adaptation through a mechanism independent of the UPR and ERAD pathways. This distinction is particularly critical in plants as they lack PERK homologs—a key translational inhibition regulatory mechanism in animals^37^. Thus, this difference highlights the evolutionary significance of CDS-m^6^A as an alternative regulatory axis that mitigates translational burden on the ER for stress resilience. Furthermore, we show that CDS-m^6^A modifications occur at conserved RRACH and URUAY motifs^1^, suggesting that this mechanism may be conserved across plants and animals. Notably, CDS-m^6^A-mediated RNA decay is not exclusive to stress conditions. Our analyses reveal that these events also occur in unperturbed cells, with GO enrichment implicating transcriptional regulatory networks. This indicates that CDS-m^6^A may participate in broader regulatory events to fine-tune developmental processes.

The subcellular localization of m^6^A marks is a critical determinant of their functions^82^. In the cytoplasm, m^6^A-modified mRNAs, mainly the ones with m^6^A in the 3’ UTR, are dynamically sorted into stress SGs^15,19,22,24^ or P-bodies^25^ for storage or degradation. Here our work establishes the ER as a new and distinct subcellular fraction of m^6^A action. This function is critical as the membrane associated compartment is responsible for producing over one-third of proteins^37^. We found that nearly half of CDS-m^6^A sites co-localize with ribosome. Also, an early mass spectrometry of the *Arabidopsis* m^6^A readers captured numerous RPF-associated and ER-localized proteins, including BiP and HSPs^33^. Thus, these observations set up a direct link m^6^A to ER-associated translation machinery. To test whether this spatial association drives RNA turnover, we leveraged the high abundance of viral transcripts to overcome the low steady-state levels of degradation intermediates^46^. This approach revealed that CDS-m^6^A deposition, together with ribosome co-accumulation, actively promotes co-translational RNA degradation. Therefore, the functional role of CDS-m^6^A modification on the ER differs from the one associated with SGs and P-bodies, which predominantly involve 3’ UTR-localized m^6^A. This distinction explains the statistically observed pattern whereby CDS-m^6^A correlate more strongly with RNA degradation^15,22,24,25^. By identifying a causal, organelle-specific function for CDS-m^6^A, our study not only uncovers an unrecognized mechanism of RNA surveillance at the ER, but also redefine how subcellular localization shapes the regulatory landscape of the epitranscriptome.

Co-translational RNA degradation (CTRD) has long been recognized as a key mechanism for controlling mRNA stability, most notably exemplified by nonsense-mediated decay (NMD) during development and stress responses^45^. In plants, CTRD was first demonstrated under heat stress, where it fine-tunes transcript stability to support adaptive responses^46,83,84^. Degradome sequencing, which captures 5′-monophosphate termini of uncapped RNAs, further revealed a pervasive landscape of RNA degradation intermediates, indicating that CTRD is a widespread process^48,49^. Yet the identity of RNA substrates, molecular triggers, and biological consequences of CTRD have remained elusive. Here, we uncover m^6^A as a central epitranscriptomic mark driving translation-dependent mRNA degradation. We show that under basal conditions, a substantial fraction of CTRD events is mediated by CDS-localized m^6^A, and that ER stress amplifies this process through increased m^6^A deposition. Enhanced m^6^A-mediated turnover facilitates the release of ribosome-engaged RNAs, thereby alleviating ER translational overload. Critically, this mechanism extends to viral RNAs beyond endogenous transcripts. Geminivirus contains a compact viral genome and lacks clear 3` UTRs, with m^6^A modifications reported to be predominantly enriched within CDS regions^81,85,86^. Importantly, these CDS-m^6^A modifications serve as signals for degradation, thereby contributing to host antiviral defense during the viral infection.

In conclusion, our findings establish CDS-m^6^A as a central regulator of RNA stability and ER stress adaptation in *Arabidopsis*, with critical roles in antiviral defense. This work expands the current conceptual framework of epitranscriptomic regulation in plants by revealing an organelle-specific pathway that directly links RNA modification to protein homeostasis and immunity. Beyond advancing basic understanding, our findings underscore the potential of manipulating m^6^A-mediated processes to enhance stress resilience and pathogen resistance in crops. Future investigation into the molecular machinery and evolutionary conservation of this pathway promises to further define its significance across plant and animal systems.

## Methods and materials

No statistical methods were used to predetermine sample sizes. Randomization and blinding design were not relevant to this study.

### Plant materials and growth conditions

*Arabidopsis thaliana* ecotype Columbia (Col-0) was used for this study. *pBA-p35S::amiR-MTA*, Col-0; *pBA-p35S::amiR-MTB*, Col-0; *p35S::Flag-MTA*, Col-0, and *p35S::Flag-MTB*, Col-0 were generated in a previously study^59^. Col-0 (WT), mutants and transgenic lines were grown on soil (Jorry Gardener/LP5) or half-strength Murashige and Skoog (MS) plates containing 1% sucrose under a 12-h light/12-h dark regime at 22° ± 1°C and ~50% relative humidity.

### Plasmid construction

The sequences were initially introduced into the pENTR vector (Invitrogen, A10462) using the primers listed in Supplementary Table 5. Following sequence verification, the entry clones were subsequently recombined into destination vectors via attL–attR (LR) recombination reactions.

For constructs containing the 3′ UTRs of *AT2G34730* and *AT1G09580*, the full-length sequences were first amplified using the primer pair F1_pEN and UTR_R2. The resulting products were then re-amplified with F1_pEN and R3_pEN to generate insertion fragments suitable for homologous recombination. In addition, PCR products amplified with F1_pEN and CDS_R1_pEN were directly used for homologous recombination. For site-directed mutagenesis constructs, the N-terminal and C-terminal fragments were first amplified separately using the primer pairs F1_pEN/N_R1 and C_F1/R3_pEN, respectively. These fragments were then assembled together with a synthesized double-stranded DNA fragment containing the desired mutation into a linearized vector via three-fragment homologous recombination.

### Treatments with Tm and DTT

Ten-day-old *Arabidopsis* seedlings were typically utilized for treatments with Tm and DTT. For microsomal fractionation and subsequent analyses, seedlings were harvested at designated time points. Tunicamycin (Sigma-Aldrich, T7765) was dissolved in DMSO and added directly to half-strength MS medium at concentrations specified. For chronic treatment, seeds were germinated directly on Tm-containing medium to assess ER stress tolerance over developmental stages. For acute treatment, 10-day-old seedlings were transferred to 30 mL of liquid MS medium supplemented with Tm, DTT, or DMSO as a control, and incubated for 2 or 4 hours prior to harvest.

### Translating ribosome affinity purification

Ten-day-old Arabidopsis seedlings were used for this assay. Approximately 1 g of frozen tissue was ground to a fine powder in liquid nitrogen and immediately resuspended in 5 mL of ice-cold polysome extraction buffer (200 mM Tris-HCl pH 9.0, 200 mM KCl, 25 mM EGTA, 35 mM MgCl_2_, 1x protease inhibitor cocktail, 1 mM DTT, 1 mM PMSF, 100 μg/mL cycloheximide, 50 μg/mL chloramphenicol, and 100 U/mL RNase inhibitor) supplemented with 1% detergent mix [20% (w/v) polyoxyethylene (23) lauryl ether (Sigma-Aldrich, 1372468), 20% (v/v) Triton X-100, and 20% (v/v) Tween 20]. Samples were incubated on ice until fully thawed. The suspension was homogenized using a rotor and incubated on ice for 10 min, followed by centrifugation at 16,000 × g for 15 min at 4°C. The supernatant was transferred to a new tube and filtered through sterile Miracloth (Millipore) to obtain a clarified extract. To isolate ribosome–mRNA complexes, the clarified extract was incubated with anti-RPL13 (Agrisera, AS13 2650, 1:200) conjugated Protein A Dynabeads at 4°C for 2 h with gentle rotation. Beads were then magnetically captured, and the supernatant was discarded. The beads were washed five times with wash buffer, each wash performed with gentle rotation for 5 min. Purified RNA was subsequently used for oligo(dT)-primed reverse transcription PCR and quantitative PCR (qPCR) analysis.

### Global mRNA decay analysis

Ten-day-old Arabidopsis seedlings were used for this assay. For each biological replicate, approximately 0.2 μg fresh weight of whole seedlings was harvested. Actinomycin D (Sigma-Aldrich, A1410) was dissolved in DMSO to prepare a 5 mg/mL stock solution, from which a fresh 100 μg/mL working solution was generated immediately prior to use. For decay analysis, seedlings were treated with 100 μg/mL Actinomycin D prior to treatment with 30 ng/mL Tm. Samples were collected at the following timepoints post-treatment: 0 h (untreated control, harvested immediately prior to Tm addition), 0.5 h, 1 h, 2 h, and 4 h.

### CaLCuV inoculation assays

*Arabidopsis* plants were initially grown on Murashige and Skoog (MS) agar plates under an 8-hour light/16-hour dark photoperiod for 10 days, followed by transfer to soil with the same light regime for approximately 14 days until reaching the developmental stage of eight true leaves. Plants of various genotypes were infected via agroinfiltration using infectious clones of CaLCuV DNA A (pNSB1090) and DNA B (pNSB1091). Freshly cultured Agrobacterium tumefaciens cells harboring the Geminivirus genome were mixed with silicon carbide powder and sprayed onto the center of newly emerged rosette leaves of *Arabidopsis* at a pressure of 80 psi using a pump sprayer. Following inoculation, plants were monitored daily for symptom development, particularly yellow mosaic and chlorosis indicative of viral infection. To evaluate systemic infection, eight newly emerged rosette leaves from CaLCuV-inoculated plants were harvested. Additionally, to assess plasmid transfection efficiency, twelve whole CaLCuV-inoculated plants (excluding cotyledons) were collected at 11-, 13-, and 16-days post-inoculation (dpi) for downstream analyses.

### Microsomal fractionation

Ten-day-old seedlings or virus-infected plants were used for microsomal fractionation. Briefly, 3 g of plant tissue was flash-frozen in liquid nitrogen and finely ground to a powder, which was then resuspended in 7 mL of lysis buffer containing 100 mM Tris-HCl (pH 7.5), 15 mM MgCl_2_, 5 mM EGTA, 250 mM sucrose, 5 mM DTT, 50 μM MG-132, 50 μg/mL cycloheximide, 50 μg/mL chloramphenicol, 2.5 U/mL SUPERase IN, 1 mM PMSF, and a protease inhibitor cocktail (Roche). A 500 μL aliquot of the lysate was reserved as the “total” fraction for subsequent protein and RNA analyses. The crude lysate was filtered twice through two layers of Miracloth and centrifuged at 10,000 ×g for 10 minutes at 4°C for twice to remove nuclei and large organelles, such as chloroplasts and mitochondria. The resulting supernatant was carefully layered onto a step sucrose gradient composed of 2.0 mL 20% sucrose overlaid on 2.0 mL 60% sucrose (prepared with the same buffer composition as above, except that 20% or 60% sucrose was used). Ultracentrifugation was performed using Beckman SW41Ti rotors in Ultra-Clear tubes (Beckman Coulter, C14277) at 38,000 rpm (~248,000 ×g) for 2 hours at 4°C. Following centrifugation, tubes were carefully removed, and individual cellular fractions were collected and transferred to clean microcentrifuge tubes for downstream applications.

### Liquid chromatography-mass spectrometry / mass spectrometry

Approximately 200 ng of purified mRNA was enzymatically digested using Nuclease P1 (NEB, M0660S) at 37°C for 6 hours. Subsequently, NH4HCO3 was added to a final concentration of 110 mM, along with 1 unit of alkaline phosphatase (Sigma-Aldrich, P6774), and the mixture was incubated at 37°C for an additional 6 hours. Enzymatic reactions were terminated by heating the samples to 70°C for 10 minutes, followed by a 5-minute incubation on ice. After centrifugation at 21,000 × g for 15 minutes, the supernatants were collected for analysis. Commercially available pure adenosine (Sigma-Aldrich, A1852) and m^6^A (TargetMol, T6599) standards were processed in parallel using the same protocol to generate standard curves with a range of concentrations.

Ultra-performance liquid chromatography (UPLC) was performed using a C18 reversed-phase column directly coupled to an Agilent 6460 mass spectrometer. Nucleosides were identified based on retention times and characteristic mass transitions (268 → 136 for adenosine, and 282 → 150 for m^6^A). Quantification of nucleosides was achieved by comparing sample peak intensities to standard curves generated from purified nucleoside standards analyzed within the same experimental batch.

### Western blot

The blots were detected with primary antibodies against m^6^A (Cell Signal Technology, 56593S, 1:200), RPL13 (Agrisera, AS13 2650, IB 1:5000), Actin (Sigma-Aldrich, A0480, IB 1:5000), Anti-NRPB2 (Agrisera, AS20 4376, ChIP 1:200), UGPase (Agrisera, AS14 2813, IB 1:5000), BiP (Agrisera, AS09 481, IB 1:5000), MTA (homemade, IB 1:1000), and MTB (homemade, IB 1:1000). Secondary antibodies were goat-developed anti-rabbit (Cytiva, NA934, IB 1:3000) and anti-mouse IgG (Cytiva, NA931, IB 1:3000). The anti-MTA and anti-MTB antibodies were generated in previous study^59^ using full-length recombinant proteins from E. coli.

### RT-qPCR

RT–qPCR was performed using the primer sets listed in Supplementary table. 5. Relative gene expression levels were determined using the 2^−ΔΔCt^ method.

### MeRIP assay

Approximately 1 μg of full-length mRNA was subjected to immunoprecipitation using an anti-m^6^A antibody (Cell Signaling Technology, 56593S; 1:200 dilution). Immunoprecipitates were sequentially washed with IP buffer (20 mM HEPES, pH 7.5, 200 mM NaCl, 5 mM MgCl2; 0.5% NP-40, and 100 U/mL RNase inhibitor), high-salt wash buffer (20 mM HEPES, pH 7.5, 500 mM NaCl, 5 mM MgCl2, 0.5% NP-40, and 100 U/mL RNase inhibitor), LiCl wash buffer (0.25 M LiCl, 1% NP-40, 1% sodium deoxycholate, 20 mM HEPES, pH 7.5, and 100 U/mL RNase inhibitor), and low salt buffer (20 mM HEPES, pH 7.5, 200 mM NaCl, and 100 U/mL RNase inhibitor). Bound RNA was eluted in 200 μL proteinase K solution (20 mM HEPES, pH 7.5; 200 mM NaCl, 2 mg/mL proteinase K, 1% SDS, and 100 U/mL RNase inhibitor) at 37°C for 1 hour with agitation on a Thermomixer. RNA was then purified using a combination of Trizol extraction and RNA Clean & Concentrator Kits (ZYMO, R1017). cDNA synthesis was performed with both oligo(dT) primers and random hexamers. Enrichment of m^6^A-containing transcripts was quantified by first normalizing immunoprecipitated fractions to input, and subsequently to Col-0, for calculation of relative m^6^A enrichment.

### Processing of MeRIP-seq data

Raw data were initially filtered for a quality control and trimmed to remove the adaptor sequences by Cutadapt. Reads were aligned to *Arabidopsis thaliana* reference genome (TAIR 10) using HISAT2. High-confidence m^6^A peaks were obtained through exomePeak2 using a threshold that P < 0.05 and log_2_(enrichment) > 1. Log_2_(enrichment) represents log_2_(IP/input). Differ-methylated level represents log_2_(enrichment in mutant / enrichment in WT). To detect differential methylation levels, peak candidates calling by exomePeak2 with P < 0.99, log2(enrichment) > 0, and Deseq2 normalization were separated into m^6^A loci and background depending on whether or not the candidates overlapped with HC-m^6^A peaks, respectively. Metagene profiles for the distribution of m^6^A sites within transcripts and m^6^A assignment to transcript regions were done with the R/Bioconductor package cliProfiler (v1.6.0). The motif of m^6^A peaks was analyzed using HOMER Motif Analysis. Integrative Genomics Viewer was used to visualize the m^6^A peaks. The m^6^A sites within different transcript regions were assigned to their corresponding categories. To assess the impact of m^6^A on transcript stability, multiple modifications within the same region of a given transcript were consolidated into a single count for that category; In contrast, transcripts bearing m^6^A sites across multiple regions were accounted for independently in each corresponding category.

### Processing of Ribo-seq data

Raw fastq files underwent an initial quality control filter, necessitating a quality score >25 at the 3′ end and subsequently trimmed to eliminate adaptor sequences using Cutadapt. Reads were aligned to *Arabidopsis thaliana* reference genome (TAIR 11) using HISAT2. All of reads mapped to the mRNA were extracted to analysis their overlap with m^6^A loci. MACS2 was used for peak calling with default parameters. Metagene analysis was conducted using the summit of RPFs.

### Processing of Degradome-seq and PARE-seq data

Raw Degradome-seq^67^ and PARE-seq^68^ reads were subjected to quality control and adaptor trimming using Cutadapt. Clean reads were then aligned to the Arabidopsis thaliana reference genome (TAIR10) using HISAT2 for degradome-seq data and STAR for PARE-seq data, respectively. Only uniquely mapped reads were retained for downstream analyses. A degradation initiation site was defined as a genomic position supported by at least 10 reads for degradome-seq data or 20 reads for PARE-seq data. And the degradation initiation sites located between 13 nucleotides upstream of the start codon and 17 nucleotides upstream of the stop codon were considered to occur within the CDS region. To calculate the Transcript Degradation Index (TDI), transcript-level abundance of degradation products was quantified as TPM for degradome-seq data and RPM for PARE-seq data. These values were normalized by the corresponding transcript abundance derived from RNA-seq data used as input for TRAP-seq.

### Degradome PCR

Aboveground tissues from *Arabidopsis thaliana* at 15 days post imbibition (DPI) and leaves from *Nicotiana benthamiana* at 5 DPI were collected for degradome PCR analysis. Approximately 0.5 g of frozen plant tissue was ground into fine powder and total RNA was extracted using TRIzol reagent. Genomic DNA contamination was removed by treatment with TURBO DNase. DNase-treated RNA was purified using the RNA Clean & Concentrator (RCC) kit (ZYMO, R1017). For adaptor ligation, 2 μg of total RNA was ligated to 1 μL of 50 μM RNA adaptor using T4 RNA ligase 1 (NEB, M0437M) ligation reaction and incubated at 16 °C overnight. Ligation products were purified using the RCC kit and reverse transcribed using oligo(dT) primers and SuperScript^™^ IV Reverse Transcriptase (Invitrogen, 18090050). The first round of PCR amplification was performed using a forward primer complementary to the RNA adaptor and a gene-specific reverse primer located at the 3′ end of the target transcript. PCR amplification was carried out for 25–32 cycles for gel visualization or 15 cycles for subsequent nested PCR. Nested PCR was performed using primer pairs with no sequence overlap with the first-round. The second-round PCR was amplified for 10–16 cycles and PCR products were analyzed by agarose gel electrophoresis.

### ChIP assays

Ten-day-old seedlings of Col-0 and *mta* were crosslinked in buffer containing 20 mM HEPES (pH 7.4), 0.4 M sucrose, 1 mM EDTA, 1 mM PMSF, and 1% formaldehyde, then ground to a fine powder in liquid nitrogen. Nuclei were isolated from 2 g of this powder and resuspended in sonication buffer (40 mM Tris-HCl, pH 7.4; 150 mM NaCl; 5 mM MgCl2; 1% SDS; 1 mM DTT; 1% Triton X-100; 2% glycerol; 1 mM PMSF; 50 μM MG-132; 1 pellet/10 mL complete EDTA-free protease inhibitor; 100 U/mL RNase inhibitor). Chromatin was sheared by 15 cycles of sonication (30 s on/90 s off). The resulting supernatant was diluted 1:9 with dilution buffer (same composition as above except that SDS was omitted). Immunoprecipitation was carried out overnight at 4 °C with agitation using anti-NRPB2 (PhytoAB, PHY2429S). Immunoprecipitates were sequentially washed with dilution buffer, high-salt buffer (20 mM Tris-HCl, pH 7.4; 500 mM NaCl; 5 mM MgCl2; 50 μM ZnCl2; 1 mM DTT; 1 mM PMSF; 0.5% Triton X-100; 50 μM MG-132; 1 pellet/10 mL protease inhibitor), LiCl buffer (0.25 M LiCl; 1% NP-40; 1% sodium deoxycholate; 20 mM Tris-HCl, pH 7.4; 1 mM EDTA), and proteinase K buffer (20 mM Tris-HCl, pH 7.4; 200 mM NaCl). De-crosslinks were performed in proteinase K solution (20 mM Tris-HCl, pH 7.4; 200 mM NaCl; 1 mg/mL proteinase K; 1% SDS; 10 mM EDTA) for 6 hours at 65 °C on a thermomixer. Samples were subsequently treated with RNase. DNA was purified using the DNA Clean & Concentrator kit (ZYMO, D4004). ChIP-qPCR was quantified by normalizing the immunoprecipitated fraction to input DNA and then to Col-0, which was arbitrarily set as 1.

## Extended Data

**Extended Data Fig 1.**
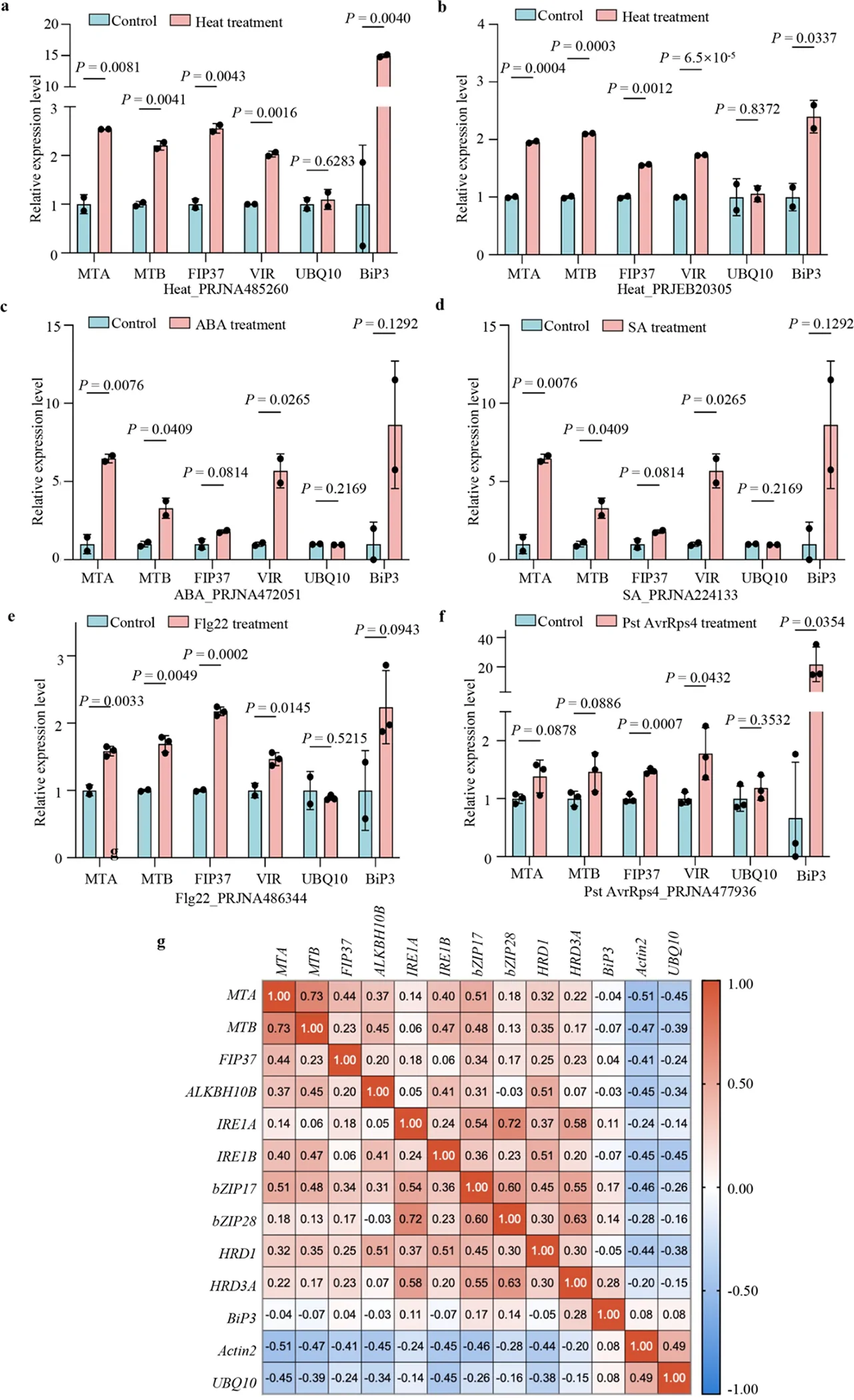
RNA-seq and pan-transcriptome analyses show gene expression correlation between the m^6^A pathway and ER stress response. a–f. RNA-seq analysis reveals increased expression of m^6^A writer complex components under heat stress (a, b), treatment with ABA (c), treatment with SA (d), treatment with flagellin 22 (e), and treatment with Pst AvrRps4 (f). Data represent mean ± s.d from two (a–d) or three (e,f) independent replicates. P values were calculated using an unpaired two-sided t-test. g. Transcriptome-wide analysis demonstrates a strong positive expression correlation between components in m^6^A pathway and ER stress response pathways. *Actin2* and *UBQ10* serve as reference controls. Correlation, Pearson coefficients.

**Extended Data Fig 2.**
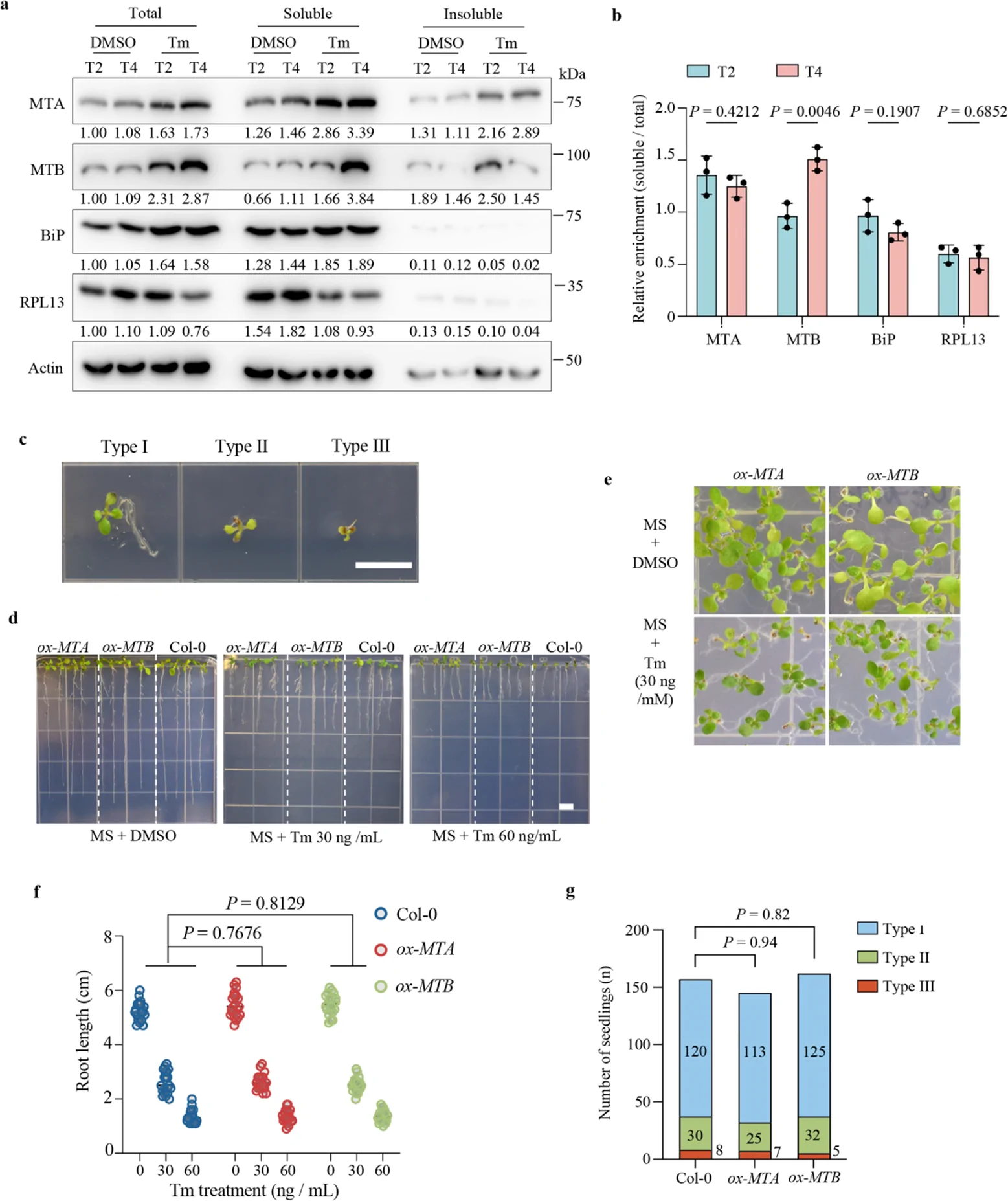
Molecular and phenotypic analyses show that the expression and function of m^6^A writers are activated upon treatment with Tm. a,b, Immunoblots (a) and statistical analysis (b) of sedimentation assays demonstrate the increasement of soluble m^6^A writers in treatment with Tm vs DMSO control. Actin was a loading control. The ratio was calculated as (soluble target / soluble Actin) / (total target / total Actin). Data represent mean ± s.d. from three independent replicates. P values were calculated using an unpaired two-sided t-test. c, Images depicting three typical morphological phenotypes of Col-0 plants upon treatment with Tm. d-g, Images (d,e) and quantitative analyses (f,g) of m^6^A writers’ overexpression lines show comparable response to treatment with Tm relative to wild type control plants. For c-g, photographs were taken of 10-day-old seedlings. Scale bars, 1 cm. For f, g, data represent at least 20 independent plants.

**Extended Data Fig 3.**
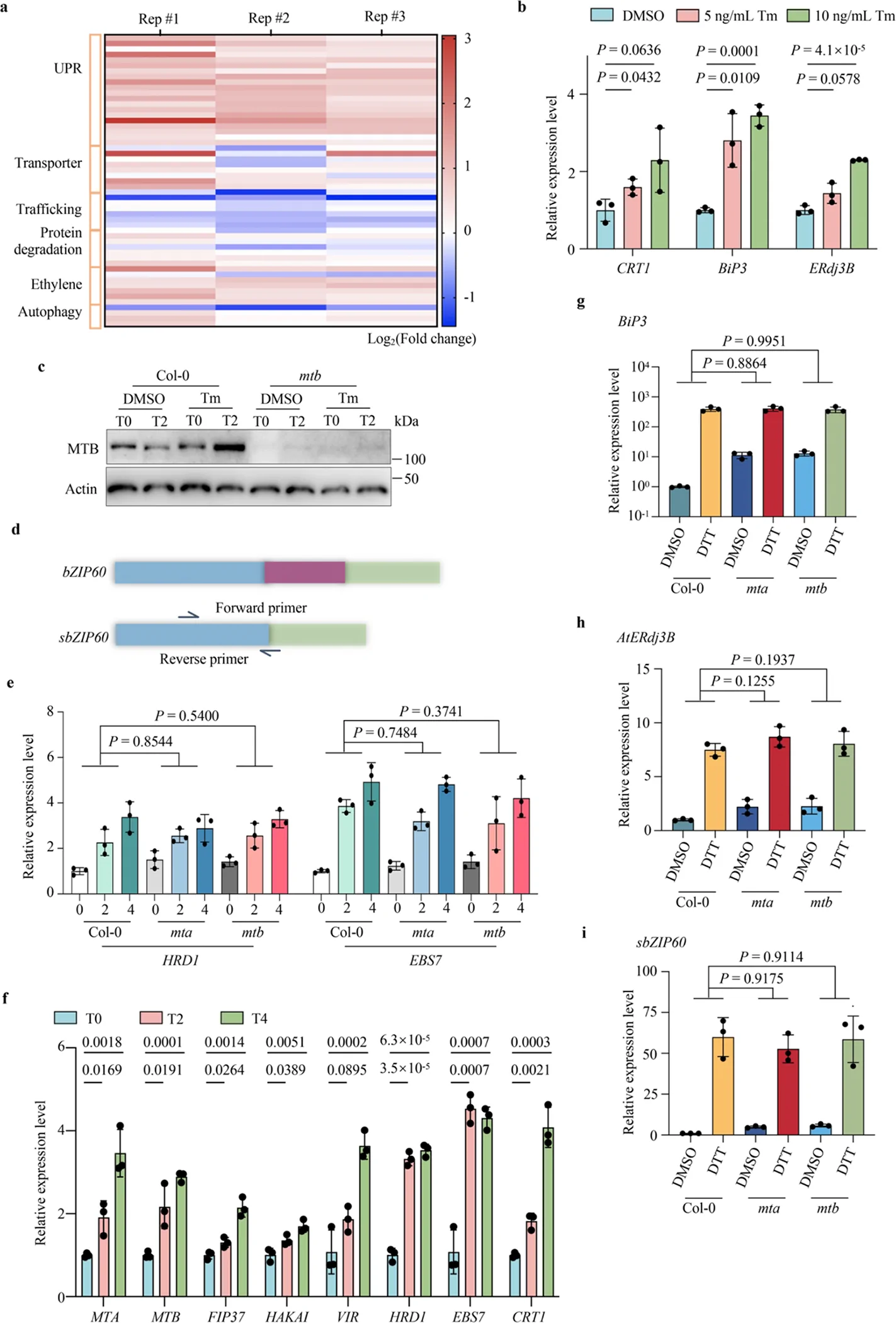
Expression and phenotypic analysis of ER stress response and m^6^A modification under treatment with Tm. a, Heatmap illustrating expression levels of ER-related pathways in *mta*, with an overall upregulation of UPR related genes in *mta* versus Col-0. b, Expression levels of ER stress response markers in seedlings subjected to chronic treatment with Tm for two weeks. P values were obtained by an unpaired two-side t-test. c. Immunoblot analysis using the antibody specifically against the endogenous protein shows that the accumulation of m^6^A writer MTB remained reduced in amiR knock mutants after treatment with Tm. Actin served as a loading control. At least three independent experiments were performed, and representative images are shown. d. Images depict that the primer set specifically amplifies the cytoplasmic spliced form of *bZIP60* (bottom), which accumulates upon ER stress activation, but not the unspliced form (top). e, RT-PCR analysis reveals elevated expression of ERAD pathway genes in both Col-0 and m^6^A writer mutants following treatment with Tm. f, RT-PCR analysis reveals that DTT-induced ER stress triggers transcriptional activation of m^6^A writers and ERAD pathway genes. P values are indicated above the bars. g–i, RT-qPCR analysis demonstrates the expression of UPR markers *AtERdj3B* (g), *BiP* (h), and spliced *bZIP60* (i) are increased in *mta* and *mtb* mutants. Values were normalized to the Col-0 value at T0, set as 1. For panels b and e–i, data are presented as means ± s.d. from three biological replicates. For normalization, *Actin* serves as an internal control. P values, unpaired two-sided t-test (b, e) and two-way ANOVA with Dunnett’s multiple comparison test (g–i).

**Extended Data Fig 4.**
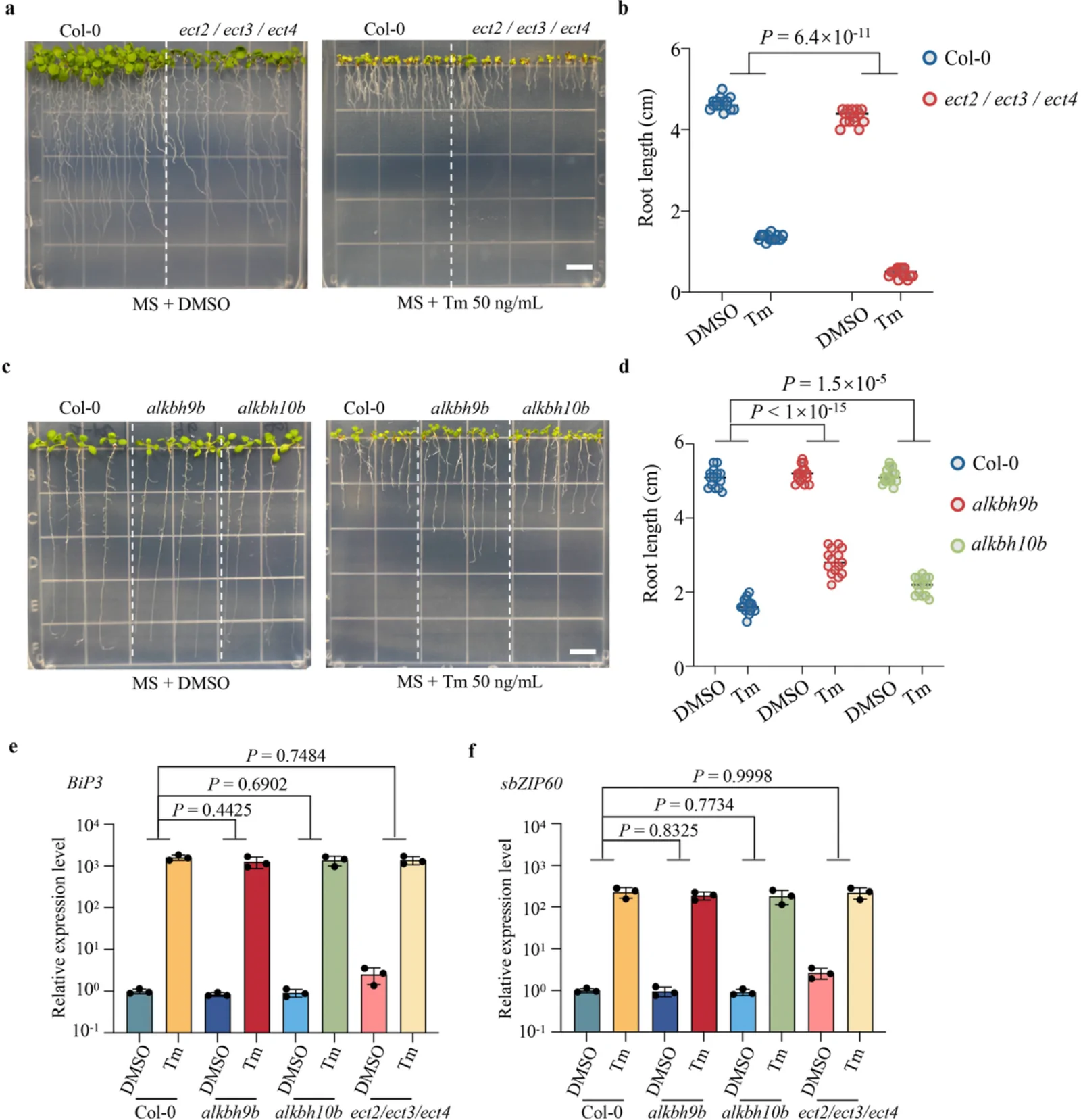
The ER stress response is altered in m^6^A reader and eraser mutants without affecting canonical UPR activation. a-d, Images (a,c) and quantitative analyses (b,d) show that m^6^A reader and eraser mutants exhibited differential tolerance or sensitivity to treatment with Tm compared with WT plants. Photographs were taken of 10-day-old seedlings. Data represent at least 15 independent plants Scale bars, 1 cm. e,f, RT-qPCR analysis demonstrates the expression of UPR markers *BiP* (e) and *sbZIP60* (f) are increased in mutants. Values were normalized to the Col-0 value at T_0_ (set as 1). Data, means ± s.d. from three biological replicates. *Actin* serves as an internal control. P values, two-way ANOVA with Dunnett’s multiple comparison test (b, d–f).

**Extended Data Fig 5.**
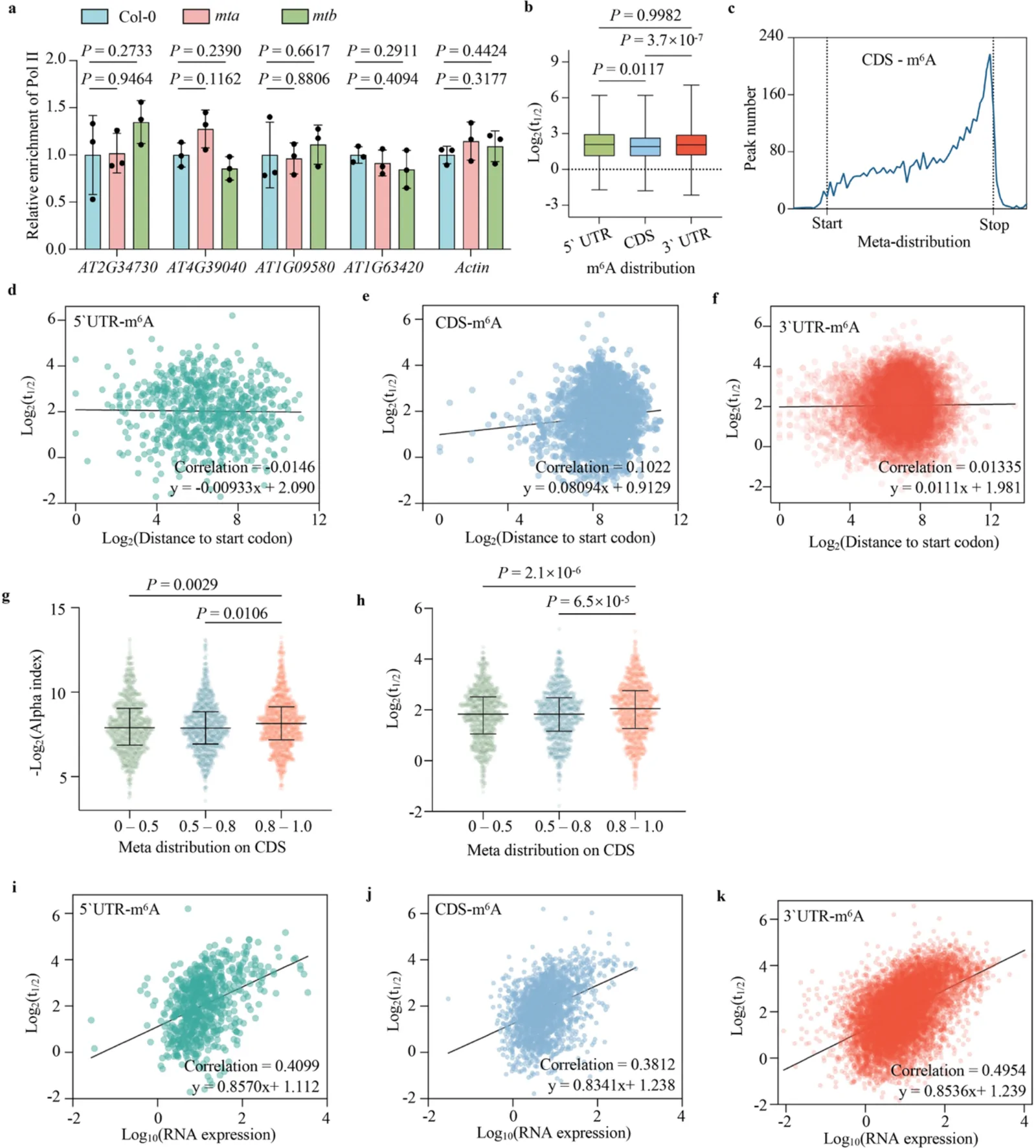
CDS-m^6^A promotes RNA degradation. a, Pol II ChIP-qPCR analysis demonstrates that reduced m^6^A levels do not affect transcription at selected loci. ChIP assays were performed using an anti-NRPB2 antibody. Relative enrichment was normalized to Col-0, set as 1. *AT2G34730*, *AT4G39040*, *AT1G09580*, and *AT1G63420* harbors CDS-m^6^A. *Actin*, which does not have CDS-m^6^A, serves as a control. Data are presented as means ± s.d. from three biological replicates. P values, unpaired two-sided t-test. b, Boxplot analysis shows the half-life variations of methylated transcripts influenced by m^6^A localization. P-values, one-way ANOVA with Tukey’s multiple comparison test. c, Metagene profile illustrates the distribution of CDS-m^6^A across the gene body. The x-axis represents the relative position along the transcript, and the y-axis indicates the number of m^6^A modification peaks. d–f, Correlation analysis of the positional distribution of 5′ UTR-m^6^A(d), CDS-m^6^A (e), and 3′ UTR-m^6^A (f) on transcripts and their RNA half-life. Correlations, Pearson coefficients. g,h, The box plots demonstrates that CDS-m6A transcripts with m^6^A sites closer to the start codon decay more rapidly and have shorter half-lives. Lines, medians; bars, quartiles. P values, one-way ANOVA with Dunnett’s multiple comparison test. i–k, Correlation analysis of transcript abundance and RNA half-life for 5′ UTR-m^6^A(i), CDS-m^6^A (j), and 3′ UTR-m^6^A (k) transcripts. Correlations, Pearson coefficients. Boxes and bars indicate interquartile ranges (25th–75th percentiles), with horizontal lines representing the medians (b, g, and h).

**Extended Data Fig 6.**
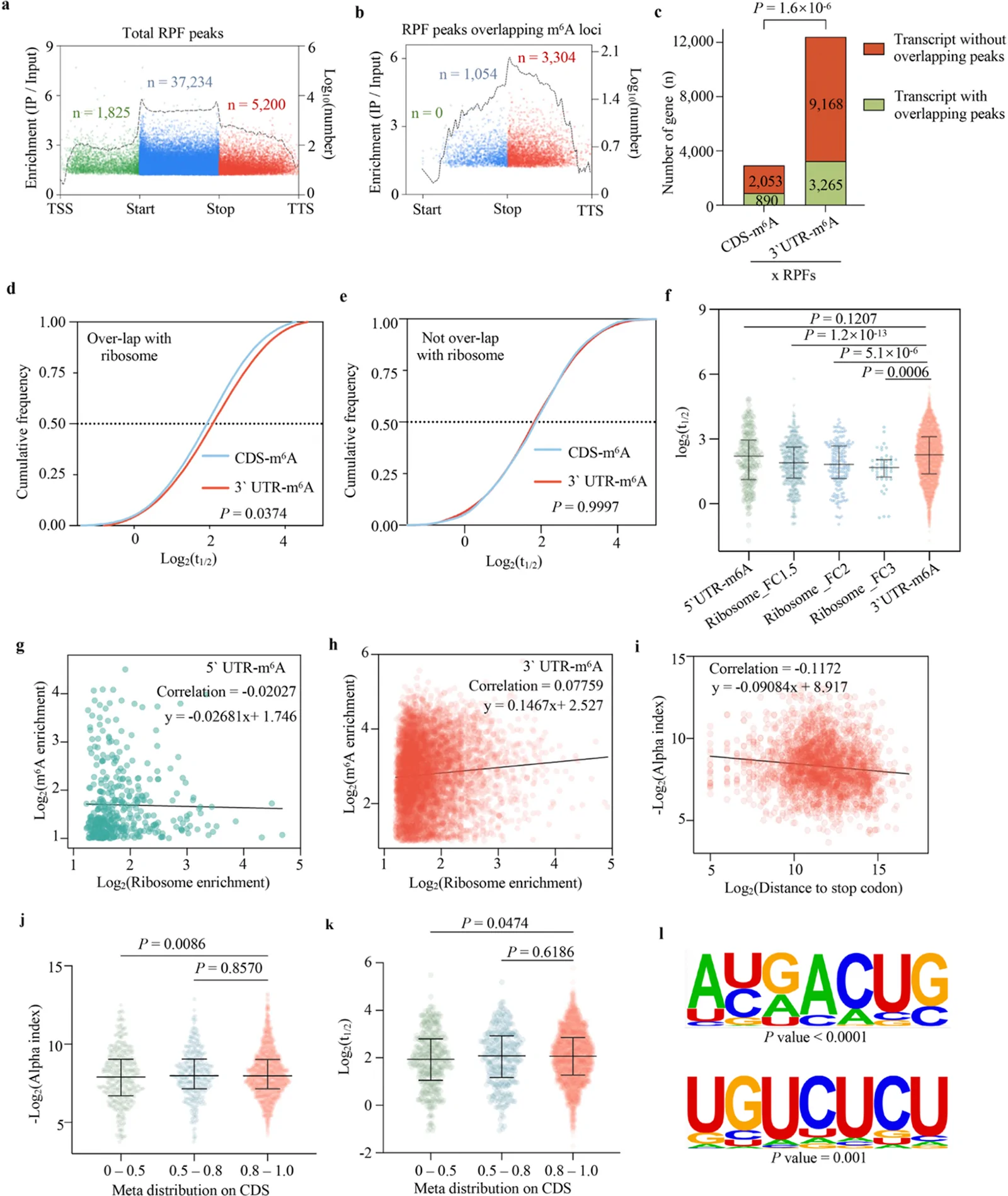
CDS-m^6^A enhances RNA degradation in the context of ribosome protected fragments overlap. a,b, Metagene analysis of (a) total RPF peaks and (b) RPF peaks overlapping with m^6^A sites. The left Y-axis represents enrichment levels of ribosome peaks (scatter plot), while the right Y-axis indicates the number of peaks at each metagene position (line plot). Scatter points are color-coded by transcript region: green, blue and red for 5′ UTR, CDS, and 3′ UTR, respectively. X-axis indicates metagene distribution of each site. c, Bar plot shows that transcripts with CDS-m^6^A sites overlap with RPF signals significantly more frequently than those with 3′ UTR-m^6^A sites. d,e, Cumulative distribution plots show the half-lives of methylated transcripts with (d) and without (e) RPF overlap. The intersection of dashed lines indicates the median degradation rate. Blue and red lines represent CDS-m^6^A and 3′ UTR-m^6^A transcripts, respectively. f, Scatter plot illustrates that CDS-m^6^A transcripts exhibit a greater reduction in RNA half-lives with increasing occupancy of ribosome in the overlapping regions. Sites were stratified by RPF enrichment levels at >1.5-, 2-, and 3-fold (IP/input) to distinguish ribosomal association. FC, foldchange. g,h, Correlation analysis of methylation levels and ribosome enrichment at identical loci for transcripts with 5′ UTR-m^6^A(g) and 3′ UTR-m^6^A (h) shows a notable weaker or opposite trend to that observed for CDS-m^6^A in Fig. 2p. i, Correlation between m^6^A loci distribution and RNA decay rate in 3′ UTR-m^6^A transcripts overlapping with ribosome peaks. j,k, The metagene profile demonstrates that transcripts with ribosome-bound and CDS-m^6^A overlapped sites closer to the start codon decay more rapidly and have shorter half-lives. j, Motif sequences associated with CDS-m^6^A modifications were identified in regions that overlap with ribosome-bound sites. Bars indicate interquartile ranges (25th–75th percentiles), with horizontal lines representing the medians (f, j, and k). P values, two-sided fisher exact test (c), Kolmogorov-Smirnov test (d,e), and one-way ANOVA with Dunnett’s multiple comparison test (f, j, and k). Correlations, Pearson coefficients (g–i).

**Extended Data Figure 7.**
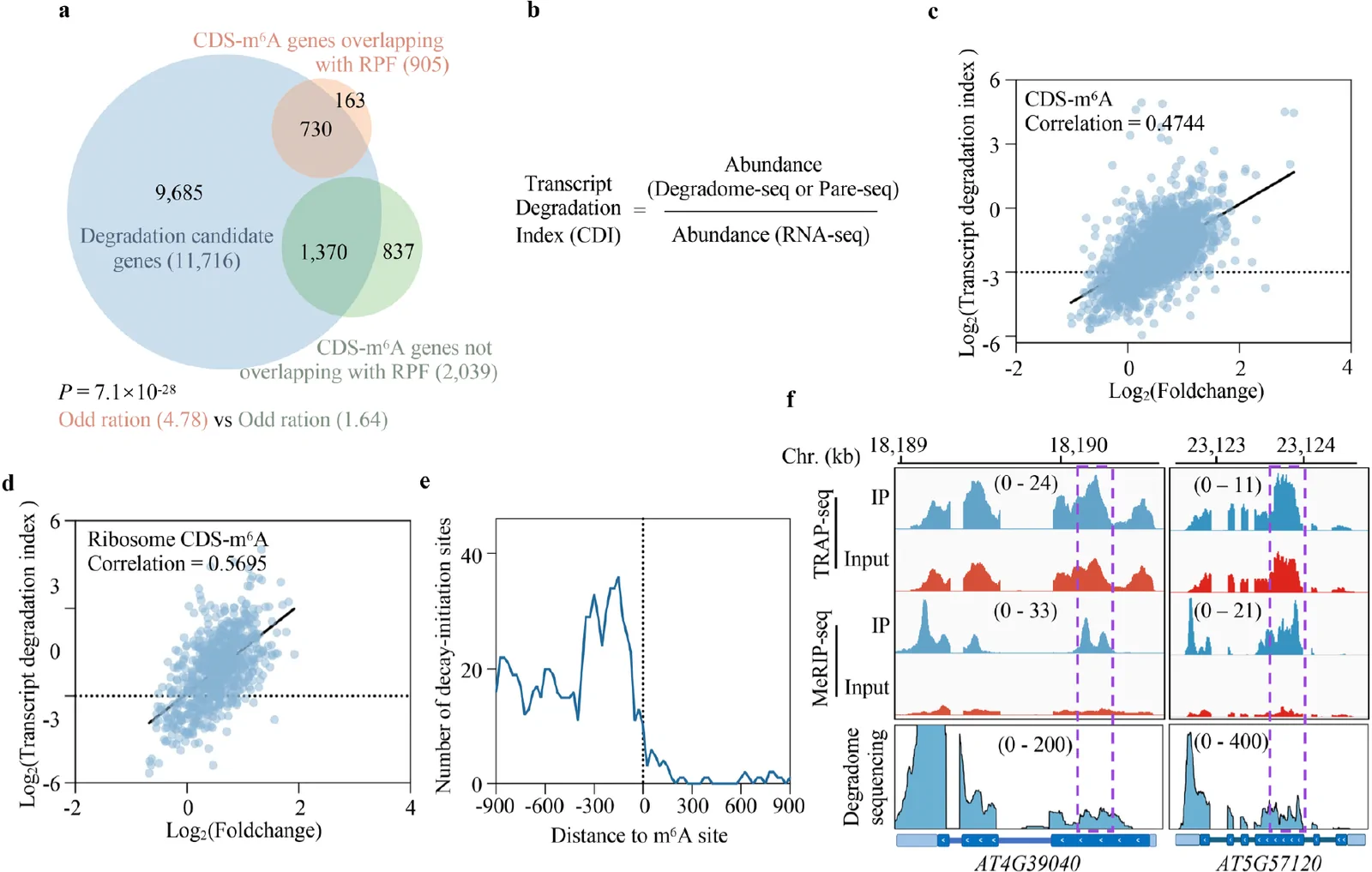
Association between RPF-CDS-m^6^A and RNA decay intermediates. a, Venn analysis shows that transcripts harboring RPF-CDS-m^6^A are highly enriched among those initiating decay within the CDS, as identified in PARE-seq. The odds ratio represents the ratio of overlapping to non-overlapping transcripts. P value, two-sided fisher exact test. b, Schematic illustrating the definition of the transcript degradome Index. c,d, Correlation between co-translational decay and expression changes in mta versus WT for transcripts with CDS-m^6^A (c) or RPF-CDS-m^6^A (d) based on PARE-seq data. Both show positive correlations, with stronger associations for RPF-CDS-m^6^A transcripts. Correlations, Pearson coefficients. e, Distance distribution between decay-initiation sites and m^6^A within transcripts containing RPF-CDS-m^6^A based on PARE-seq data. Profiles are centered on m^6^A sites; negative and positive values on the x-axis indicate positions upstream (5′) and downstream (3′) of m^6^A, respectively. Data are shown in 50-nt windows with 25-nt overlap. The y-axis denotes the number of decay-initiation sites within each window. f, IGV tracks of representative genes show the accumulation of RNA decay intermediates around loci with overlapping CDS-m^6^A and ribosome footprints. Input and IP tracks are shown in red and blue, respectively. Ribo-seq and MeRIP-seq X-axes show reads per million; Degradome-seq shows raw read counts. Chr., chromosome.

**Extended Data Fig 8.**
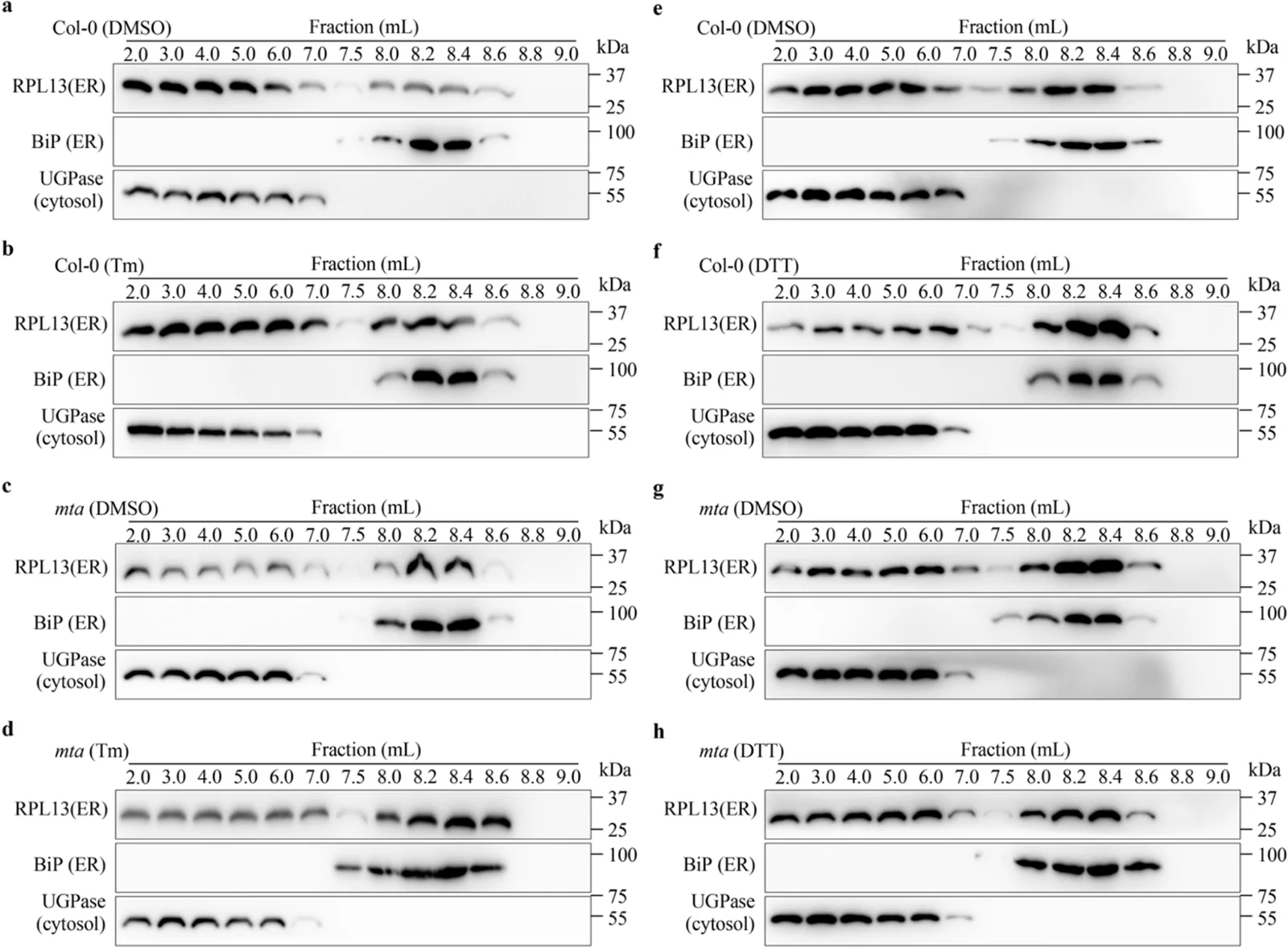
Microsomal purification for analysis of RNA distribution a–d, Immunoblots confirm the effective enrichment of microsomal fractions and their separation from cytosolic fractions under different treatments: DMSO-treated Col-0 (a), Tm-treated Col-0 (b), DMSO-treated mta (c), and Tm-treated mta (d). Panels a–d represent one of biological replicates corresponding to Fig. 4c–f. e–h, Immunoblots validate the microsomal enrichment and fractionation under DMSO- and DTT-treated conditions in both Col-0 (e, f) and mta genotypes (g, h). At least three independent experiments were performed, and representative images are shown. For a–h, immunoblots were detected with endogenous protein antibodies, respectively.

**Extended Data Fig 9.**
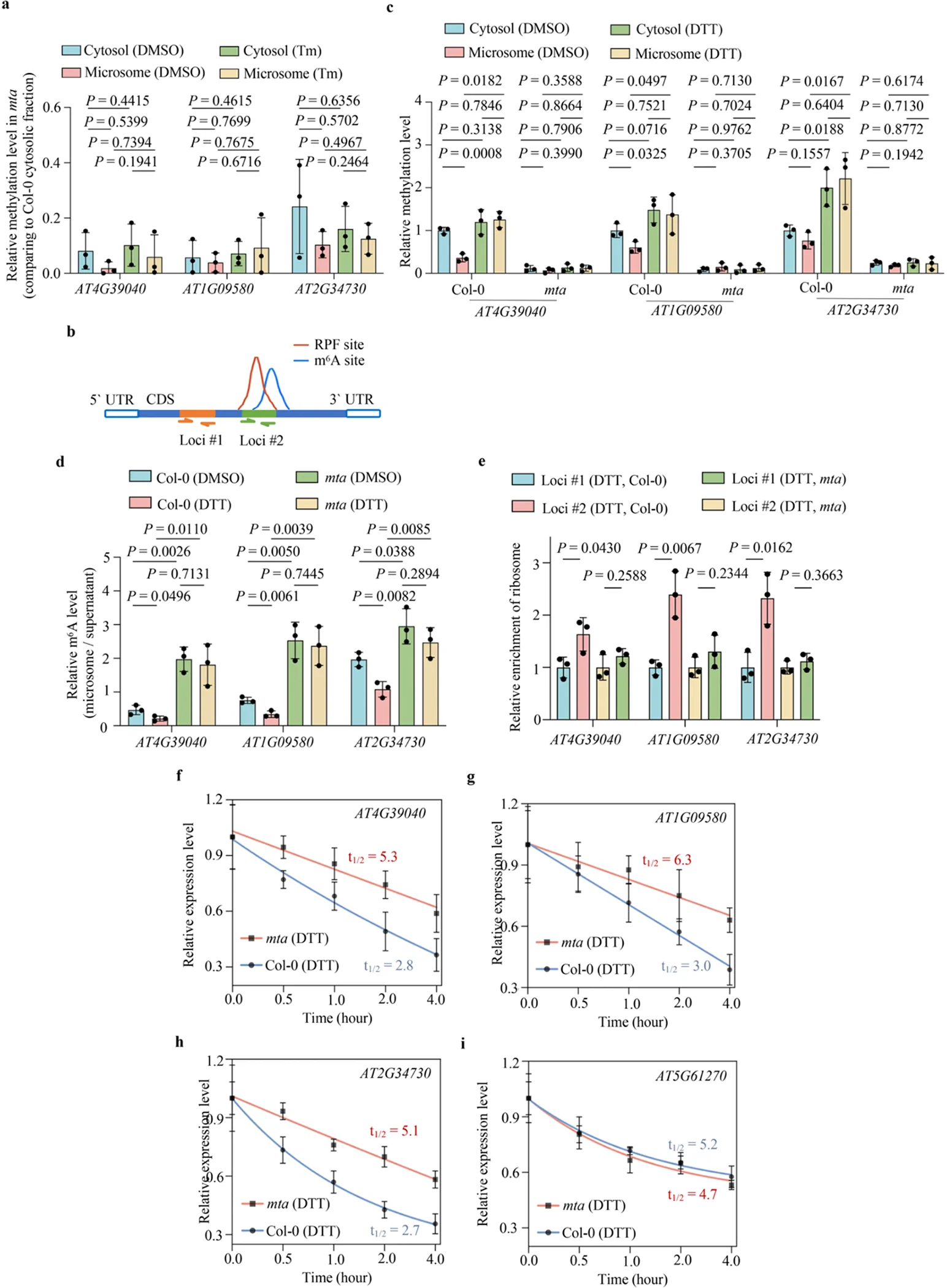
ER stress enhances CDS-m^6^A and ribosome co-accumulation and RNA degradation. a, RT-qPCR results demonstrate that treatment with Tm induced re-distribution of transcripts is compromised in *mta*. Relative enrichment was normalized to Col-0 cytosolic fraction (DMSO) in Fig. 4e which set as 1, with *Actin* serving as an internal control. b, Illustration of locus # 1 and # 2 for ribosome RIP assay. c, RT-qPCR assay demonstrates that treatment with DTT increases m^6^A levels on microsome-associated transcripts in Col-0, whereas m^6^A is markedly reduced in *mta*. For m^6^A quantification, relative enrichment was normalized to Col-0 cytosolic fraction (DMSO) which set as 1. *Actin* serves as an internal control. d, RT-qPCR assay shows that treatment with DTT reduces the microsomal association of transcripts with CDS-m^6^A in Col-0, with a less pronounced effect observed in *mta*. For transcript abundance across fractions, relative levels were calculated as (IP/Input in microsome) / (IP/Input in supernatant). e, Ribosomal RIP-qPCR analysis shows that treatment with Tm enhances ribosome enrichment at sites where ribosome signals overlap with CDS-localized m^6^A. The loci 1 and 2 are located at the 5` distal end and near the ribosome-CDS-m^6^A overlap site, respectively (illustrated in Extended Data Fig. 9b). Enrichment levels were calculated as IP/Input ratios, with relative enrichment determined by dividing the enrichment at locus 2 by that at locus 1. f–i. RT-qPCR analysis shows mRNA degradation rates of *AT4G39040* (f), *AT1G09580* (g), *AT2G34730* (h), and *AT5g61270* (i) in 10-day-old seedlings. WT and *mta* seedlings were co-treated with actinomycin D and either DMSO or Tm and sampled at indicated time points. *Actin* serves as an internal control. Half-lives, first-order exponential decay model, y(t) = y_0_e^−kt^. Curves were fitted to a one-phase decay model. For a, c-h, data are presented as means ± s.d. from three biological replicates. P values, unpaired two-sided t-test.

**Extended Data Fig 10.**
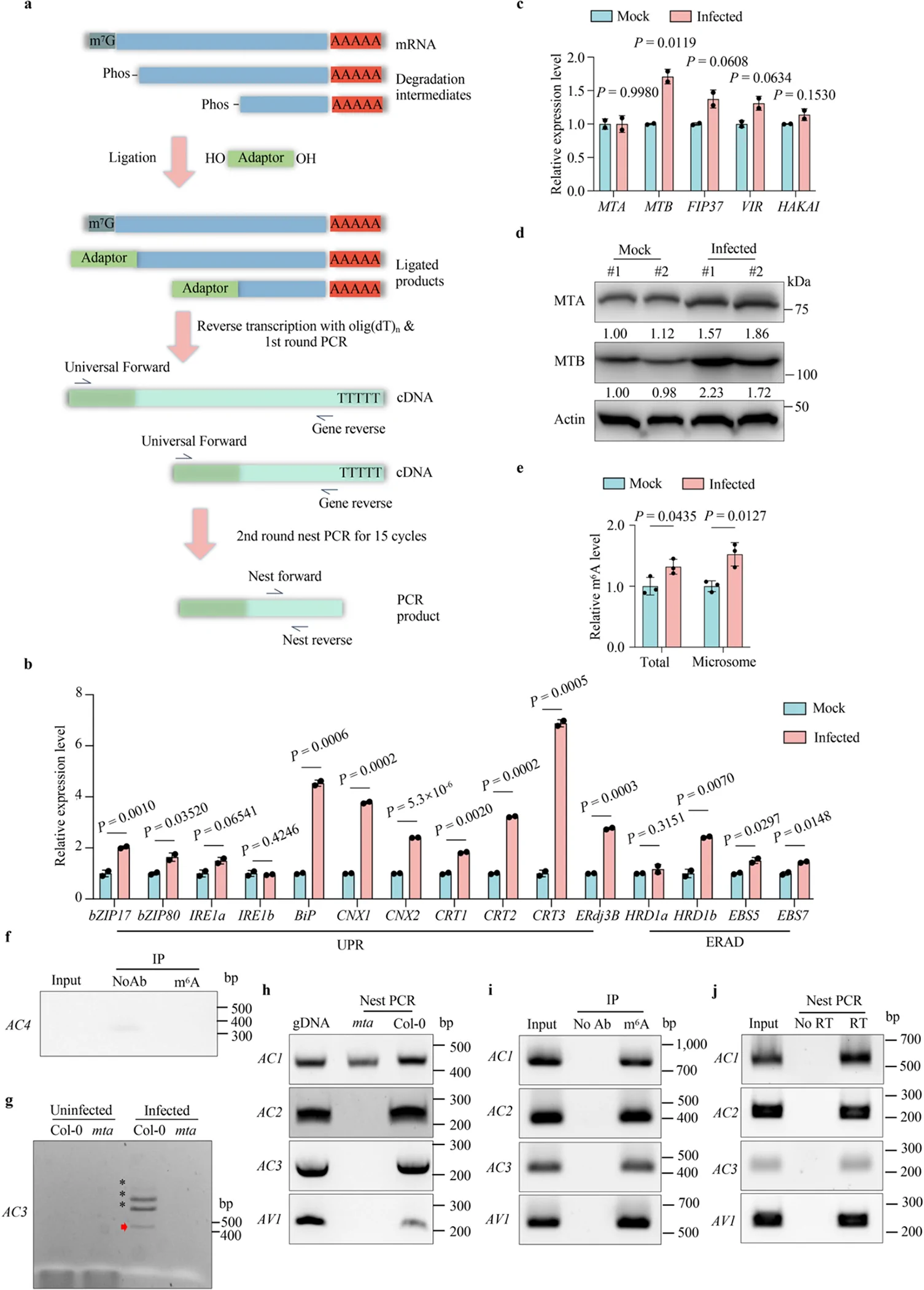
m^6^A promotes co-translational degradation of viral transcripts on the ER. a, Illustration of strategy to use degradome PCR to identify the decay intermediates of virus transcripts. b,c, RNA-seq analysis reveals increased expression levels of UPR and ERAD pathways (a) and writers (b) following viral infection in Col-0. d, Immunoblot assays show elevated writers’ protein levels after viral infection. Endogenous antibodies were used for detection. *Actin* serves as a loading control. e, Mass spectrometry result shows the increased m^6^A level in both total and microsomal mRNAs after virus infection vs mock. f, MeRIP-PCR identifies the m^6^A methylation on virus transcripts *AC4*. Con, mock control; Inf, virus infection. g, DNA gel documented that first-round amplification identified a potential virus *AC3* transcripts in degradome PCR. h, Second-round nested PCR validation validated the indeed virus transcripts identified in first round PCR. i, MeRIP-PCR identifies the m^6^A methylation on virus transcripts identified in ER fraction purified from infected tobacco. Inf, virus infected. j, Second-round nested PCR validation validated the indeed virus transcripts identified in ER fraction purified from infected tobacco. NoRT represents ligated products were not subjected to RT-PCR. For b, c, and e, data are presented as means ± s.d from three biological replicates. P values, unpaired two-sided t-test. For f–j, at least three independent experiments were performed, and representative images are shown.

## Supplementary Material

Source_Figs

Source_ numerical

Supplementary table 2 Analysis of TRAP-seq

Supplementary table 4 Analysis of PARE-seq

Supplementary table 1 Analysis of MeRIP-seq

Supplementary table 3 Analysis of degradome-seq

Supplementary table 5 List of oligos used in this study

## Figures and Tables

**Fig 1.**
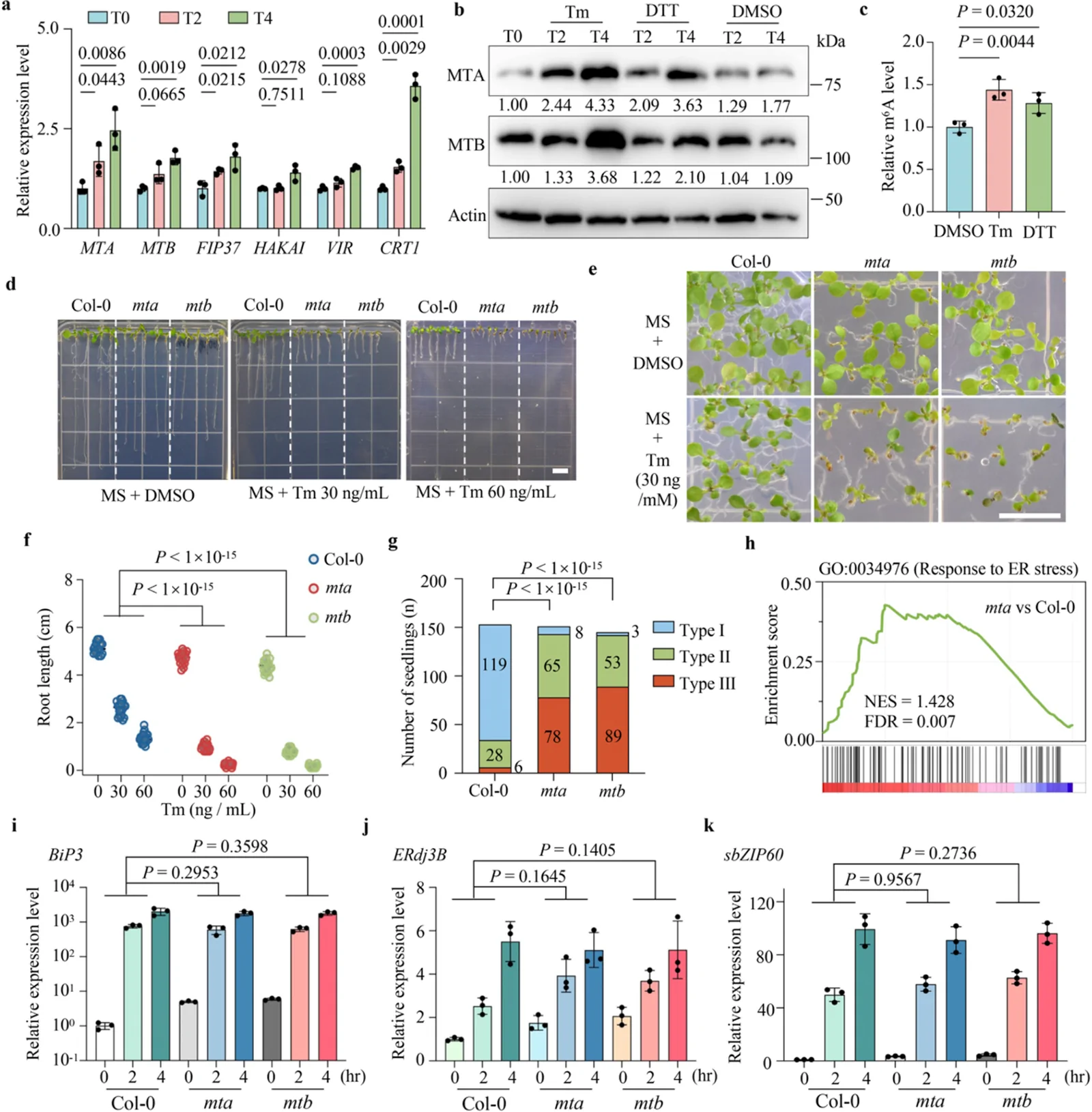
m^6^A modification is implicated in ER stress response. a,b. RT-qPCR (a) and immunoblot (b) analyses reveal significantly higher levels of m^6^A writers’ transcripts and proteins in Tm- and DTT-treated samples versus DMSO-treated controls. *VIR*, *VIRILIZER*. *CRT1*, an ER stress response gene, serves as a positive control. At least three independent experiments were performed, and representative images are shown. c. Mass spectrometry detects elevated mRNA m^6^A levels in response to treatments with Tm and DTT. d–g. Images (d,e) and quantitative analyses (f,g) show that writer mutants are more sensitive to treatment with Tm, displaying shorter roots (d,f) and dwarfed phenotypes (e,g) compared to Col-0. Photographs were taken of 10-day-old seedlings. Scale bars, 1 cm. Data represent at least 20 independent plants. h. Gene Set Enrichment Analysis (GSEA) indicates an activated ER stress response pathway in *mta* versus Col-0. Normalized enrichment score (NES) and false discovery rate (FDR q-value) are shown. A positive NES indicates pathway upregulation in *mta* vs Col-0. The bottom panel displays genes in ER stress response pathway ordered from most upregulated (left, red) to most downregulated (right, blue). i–k. RT-qPCR analysis shows increased expression of UPR markers *BiP3* (i), *ERdj3B* (j), and s*bZIP60* (k) upon treatment with Tm. Samples were collected at indicated time points. Fold changes were calculated as (treatment with Tm / DMSO) and normalized to Col-0 at T_0_ (set to 1). *Actin* was used as an internal control. For panels a, c, and i–k, data represent mean ± s.d. from three independent experiments. P-values, unpaired two-sided t-test (a,c), two-sided fisher extract test (g) and two-way ANOVA with Dunnett’s multiple comparison test (f, i–k); the values are indicated.

**Fig 2.**
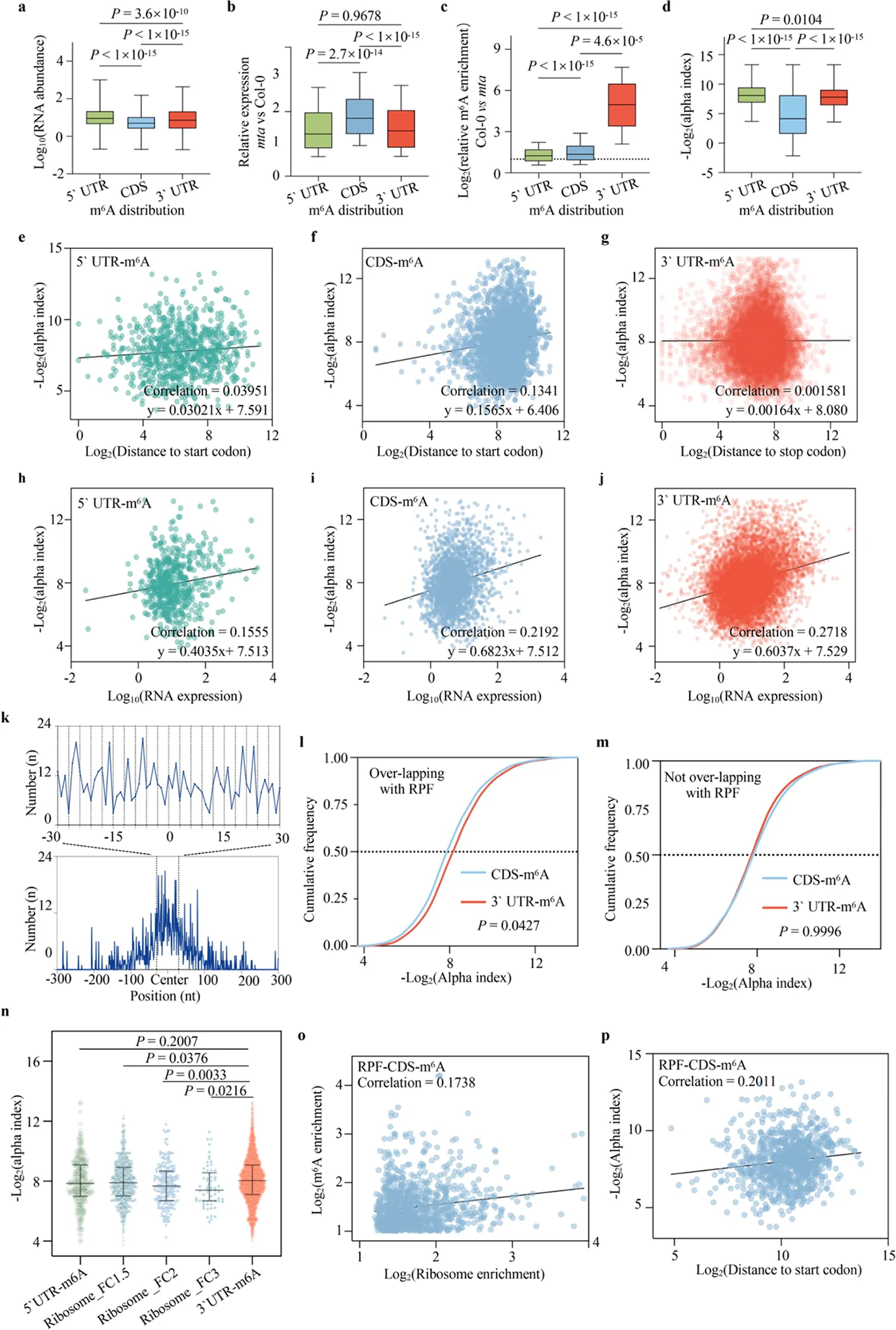
Co-localization of ribosome protected fragments and the m^6^A sites in the CDS highly correlates with RNA decay. a. Blot-plot analysis reveals that the transcripts with CDS-m^6^A exhibit the lowest RNA abundance (FPKM) compared to the ones with 5′ UTR or 3′ UTR-m^6^A modifications in WT plants. b. Transcripts with m^6^A in the CDS show a greater increase in abundance than those with m^6^A in the 5′ UTR or 3′ UTR in *mta* versus Col-0. c. The change in m^6^A levels in the CDS region between *mta* and Col-0 is comparable to that in the 5′ UTR, but much smaller compared to the much larger difference seen in the 3′ UTR. d. Transcripts with CDS-m^6^A modifications display a higher decay rate than those with m^6^A modifications in 5′ UTR or 3′ UTR. e–g. Correlation between the distance from m^6^A modifications to the start codon (e, f) or stop codon (g) and RNA decay rate shows a significant association for CDS-m^6^A transcripts, but not for those with m^6^A in the 5′ or 3′ UTR (e–g). h–j. Correlation between transcript abundance and RNA decay rates for transcripts with m^6^A in the 5′ UTR (h), CDS (i), and 3′ UTR (j). k, Distance distribution between RPFs and m^6^A sites. The profile is centered on m^6^A sites. The x-axis indicates the relative position of ribosome-protected fragments (RPFs) with respect to m^6^A sites, with negative and positive values denoting upstream (5′) and downstream (3′) positions, respectively. The Y-axis represents the number of RPFs at each corresponding position. Bottom: an overall view of distribution of overlaps. Top: A zoomed-in view of the X-axis from −30 to 30 nt. l, m, Cumulative distribution plots show the degradation rates of methylated transcripts with (l) and without (m) RPF overlap. The intersection of dashed lines indicates the median degradation rate. Blue and red lines represent transcripts with m^6^A in the CDS and 3′ UTR, respectively. n, Scatter dot plot analysis demonstrates that transcripts with m^6^A in the CDS exhibit increased RNA decay rates when overlapping with higher RPF rates. Sites were stratified by ribosome enrichment levels at >1.5-, 2-, and 3-fold (IP/input) to distinguish ribosomal association. FC, foldchange. o, Correlation between the distance from m^6^A modifications to the start codon and RNA decay rate for the transcripts with RPF-CDS-m^6^A. p, Analysis of the correlation between CDS-m^6^A level and ribosome enrichment at overlapping loci. Boxes and bars indicate interquartile ranges (25th–75th percentiles), with horizontal lines representing the medians (a–d, n). P-values, an unpaired two-sided t-test (a–d, and n), and Kolmogorov-Smirnov test (l, m). Correlations, Pearson coefficients (e–j, o, and p).

**Figure 3.**
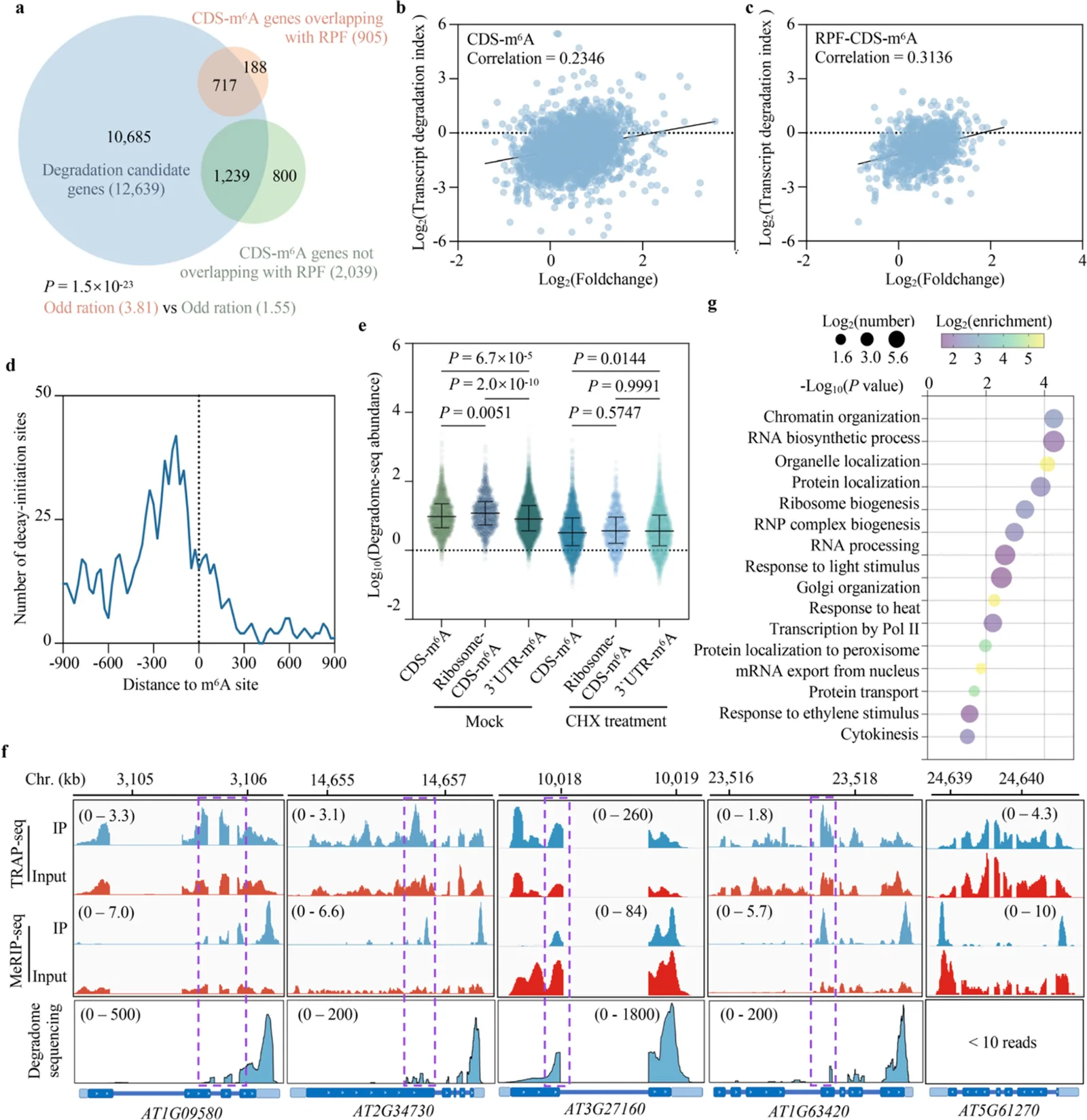
Ribosome protected fragments and the m^6^A sites in the CDS correlate with RNA decay intermediate. a, Venn analysis shows that transcripts harboring RPF-CDS-m^6^A are highly enriched among those exhibiting decay initiation within the CDS identified in degradome-seq. The odds ratio represents the ratio of overlapping to non-overlapping transcripts. b,c, Correlation between TDI and expression changes in *mta* vs Col-0 for transcripts with CDS-m^6^A (b) or RPF-CDS-m^6^A (c). Both show positive correlations, with stronger associations for RPF-CDS-m^6^A transcripts. Correlations, Pearson coefficients. d, Distance distribution between decay-initiation sites and m^6^A within transcripts containing RPF-CDS-m^6^A. Profiles are centered on m^6^A sites. X-axis, negative and positive numbers indicate positions upstream (5′) and downstream (3′) sides flanking m^6^A loci, respectively. Data are shown in 50-nt windows with 25-nt overlap. Y-axis, the number of decay-initiation sites within each window. e, Scatter plot shows reduced statistic differences in intermediate abundance among transcripts with indicated m^6^A distribution after treatment with CHX. Lines, medians and quartiles. P values, one-way ANOVA with Tukey’s multiple comparison test. Bars indicate interquartile ranges (25th–75th percentiles), with horizontal lines representing the medians. f, Integrated Genomics Viewer (IGV) profiles of representative genes illustrate the accumulation of RNA decay intermediates around loci with overlapping CDS-m^6^A and ribosome signals. Input and IP tracks are shown in red and blue, respectively. Chr., chromosome. Ribo-seq and MeRIP-seq X-axes represent reads per million, while Degradome-seq shows raw read counts. *AT5G61270* lacks sufficient reads (>10) in the CDS region, resulting in a blank track. g, GO analysis of transcripts with overlapping degradome signals and RPF-CDS-m^6^A. P values, two-sided fisher exact test (a) and one-way ANOVA with Dunnett’s multiple comparison test (e).

**Figure 4.**
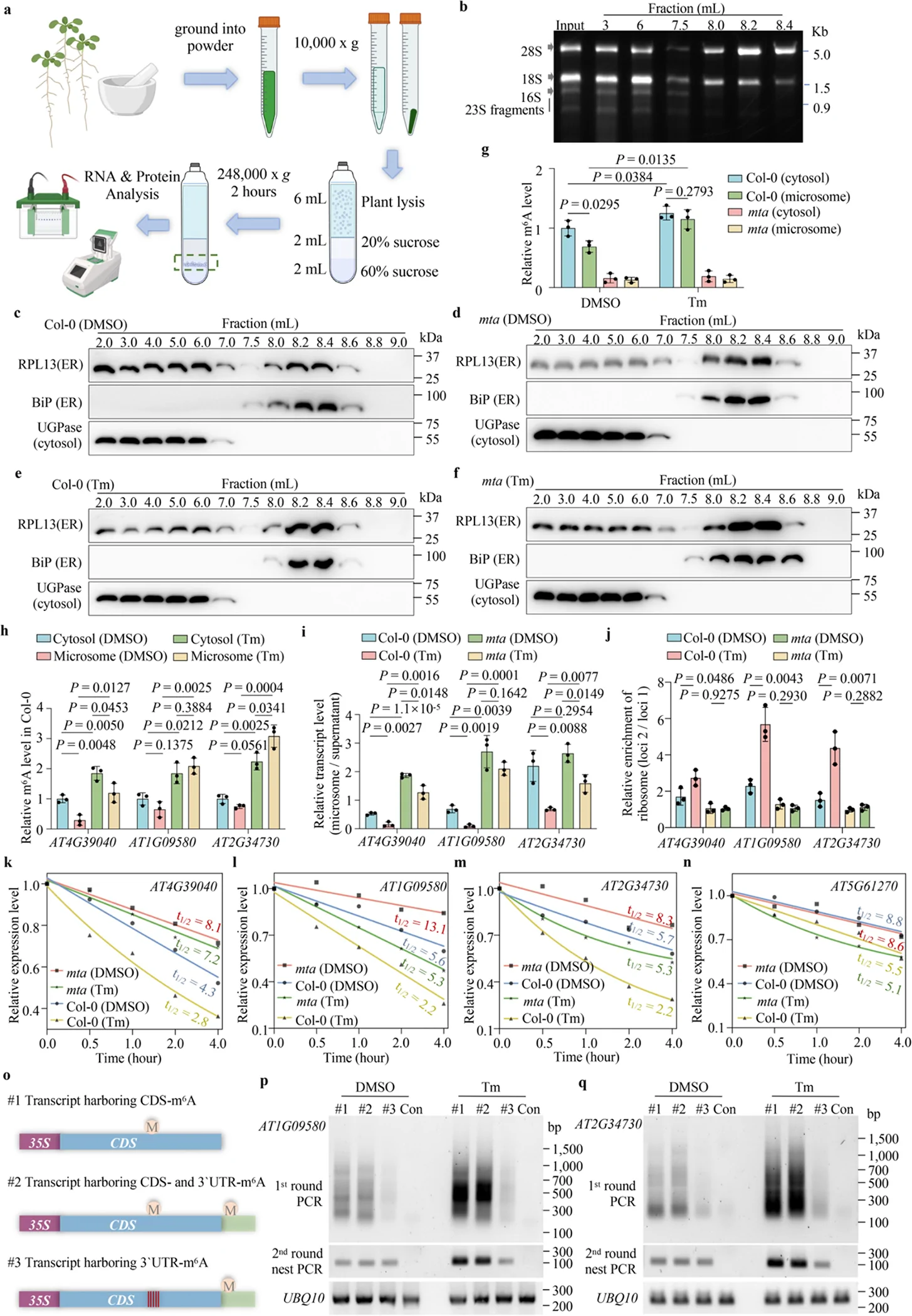
Stress-induced CDS-m^6^A on microsomes promotes RNA degradation. a, Schematic illustration of microsome extraction from plant powder for RNA and protein assays. Briefly, plant supernatant was filtered twice through Miracloth, followed by centrifugation at 10,000 x g to remove nuclei and chloroplasts. The resulting soluble supernatant was subjected to sucrose gradient centrifugation to enrich for higher sedimentation coefficient microsomal fractions. BiP and RPL13 serves as marks. See Methods for detailed procedures. Created with Biorender.com. b, RNA electrophoresis reveals that microsomal fractions are enriched in nuclear-encoded 28S and 18S rRNA, without chloroplast rRNA species. c-f, Immunoblot analysis confirms the enrichment of microsomal fractions and their clear separation from cytosolic fractions across DMSO treated Col-0 (c), Tm treated Col-0 (d), DMSO treated *mta* (e) and Tm treated *mta* (f). g, Mass spectrometry detects increase m^6^A levels in two fractions upon ER stress. h, RT-qPCR result demonstrates that treatment with Tm increases m^6^A levels on microsome-associated transcripts in Col-0. For m^6^A quantification, relative enrichment was normalized to Col-0 cytosolic fraction (DMSO) which was set as 1. *Actin* serves as an internal control. i, RT-qPCR result demonstrates that treatment with Tm decreases microsomal association of CDS-m^6^A transcript in Col-0, with a less pronounced effect in *mta*. For transcript abundance across fractions, relative levels were calculated as [(IP / Input in microsome) / (IP / Input in supernatant)]. j, Ribosomal RIP-qPCR analysis shows that treatment with Tm enhances ribosome enrichment at sites where ribosome signals overlap with CDS-localized m^6^A. The loci 1 and 2 are located at the 5` distal end and near the ribosome-CDS-m^6^A overlap site, respectively (illustrated in Extended Data Fig. 9b). Enrichment levels were calculated as IP / Input ratios, with relative enrichment determined by dividing the enrichment at locus 2 by that at locus 1. k–n, RT-qPCR analysis shows that slower mRNA degradation rates of *AT4G39040* (k), *AT1G09580* (l), *AT2G34730* (m), and *AT5G61270* (n) in *mta* vs WT. Ten-day-old seedlings were co-treated with actinomycin D and either DMSO or Tm and sampled at corresponding time points. *Actin* serves as an internal control. Curves, one-phase decay model. Half-lives, first-order exponential decay model, y(t) = y_1_e^−kt^. o. Images shows the transcripts used for detecting RNA decay intermediates. The red line in construct #3 indicates a base substitution. p,q. PCR analysis of *AT1G09580* (p) and *AT2G34730* (q) shows that CDS-m^6^A promotes stronger accumulation of RNA decay intermediates on the ER compared with 3′ UTR-m^6^A. UBQ10, loading control. For b–f, p, and q, at least three independent experiments were performed, and representative images are shown. For g–m, data are presented as means ± SD from three independent experiments. For g–j, P-values were calculated using an unpaired two-sided t-test.

**Figure 5.**
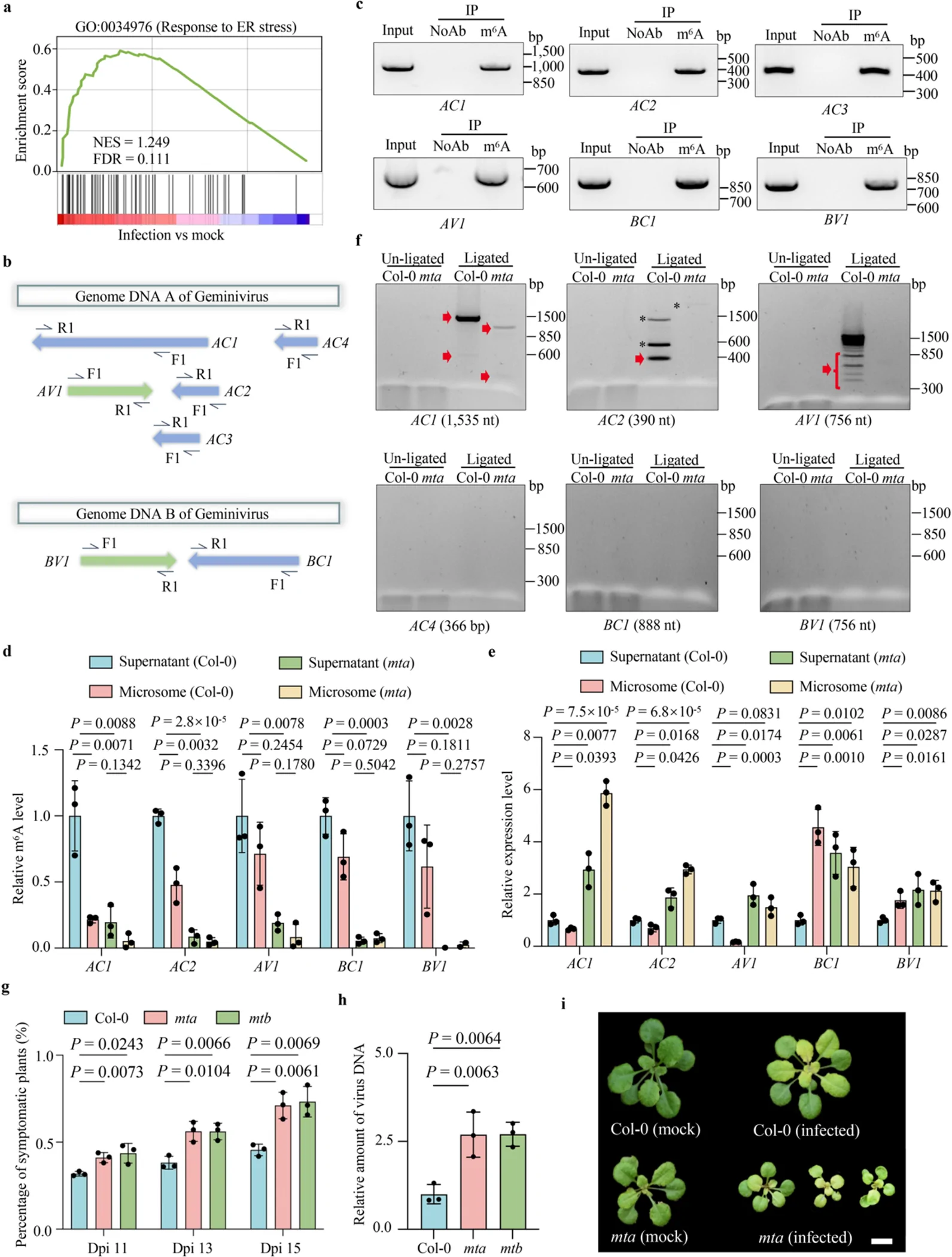
CDS-m^6^A triggers degradation of viral RNA. a, GSEA reveals an activated ER stress response pathway in Geminivirus-infected plants versus mock controls. Normalized enrichment score (NES) and false discovery rate (FDR q-value) are shown. A positive NES indicates pathway upregulation in infected vs mock plants. The bottom panel displays genes in ER stress response pathway ordered from most upregulated (left, red) to most downregulated (right, blue). b,c, Illustration of the primer design used for MeRIP-PCR (b) and corresponding results (c) showing the m^6^A methylation on virus transcripts. d, MeRIP analysis quantifies m^6^A modification levels on viral RNA in microsomal versus supernatant fractions. Enrichment was calculated as IP/Input ratios, and normalized to the value in Col-0 supernatant, which was set as 1. e, RT-qPCR analysis of viral RNA across fractions reveals the decreased accumulation of *AC1*, *AC2*, and *AV1* but increased amount of *BC1* and *BV1* in the microsomal fraction relative to cytosol in Col-0, but not in *mta*. Transcript levels in the cytosolic fraction of Col-0 were normalized to 1, with *Actin* as an internal control. f, Degradome PCR analysis of microsomal associated RNA detects degradation intermediates of viral RNA generated during co-translational processes. Red arrows indicate amplification products corresponding to putative degradation intermediates, while asterisks denote non-specific bands. g, Statistical analysis shows that *mta* and *mtb* mutants had increased symptomatic rates than Col-0. The viral symptomatic plants were counted on days 11, 13, and 15 post-inoculation. h, Viral DNA quantification shows increased virus titers in *mta* and *mtb* mutants compared to Col-0. Seedlings were collected at 16 days post-infection, and viral DNA levels were normalized to that in Col-0, which was set as 1. i, Photographs of mock and virus-infected plants demonstrate that m^6^A writer mutants were hypersusceptible to viral infection than Col-0. At least 60 plants were used for virus infection with representative plants selected for imaging. For c, f, at least three independent experiments were performed, and representative images are shown. For panels d, e, g, and h, data represent mean ± s.d. from three independent experiments. P values, unpaired two-sided t-test.

**Figure 6.**
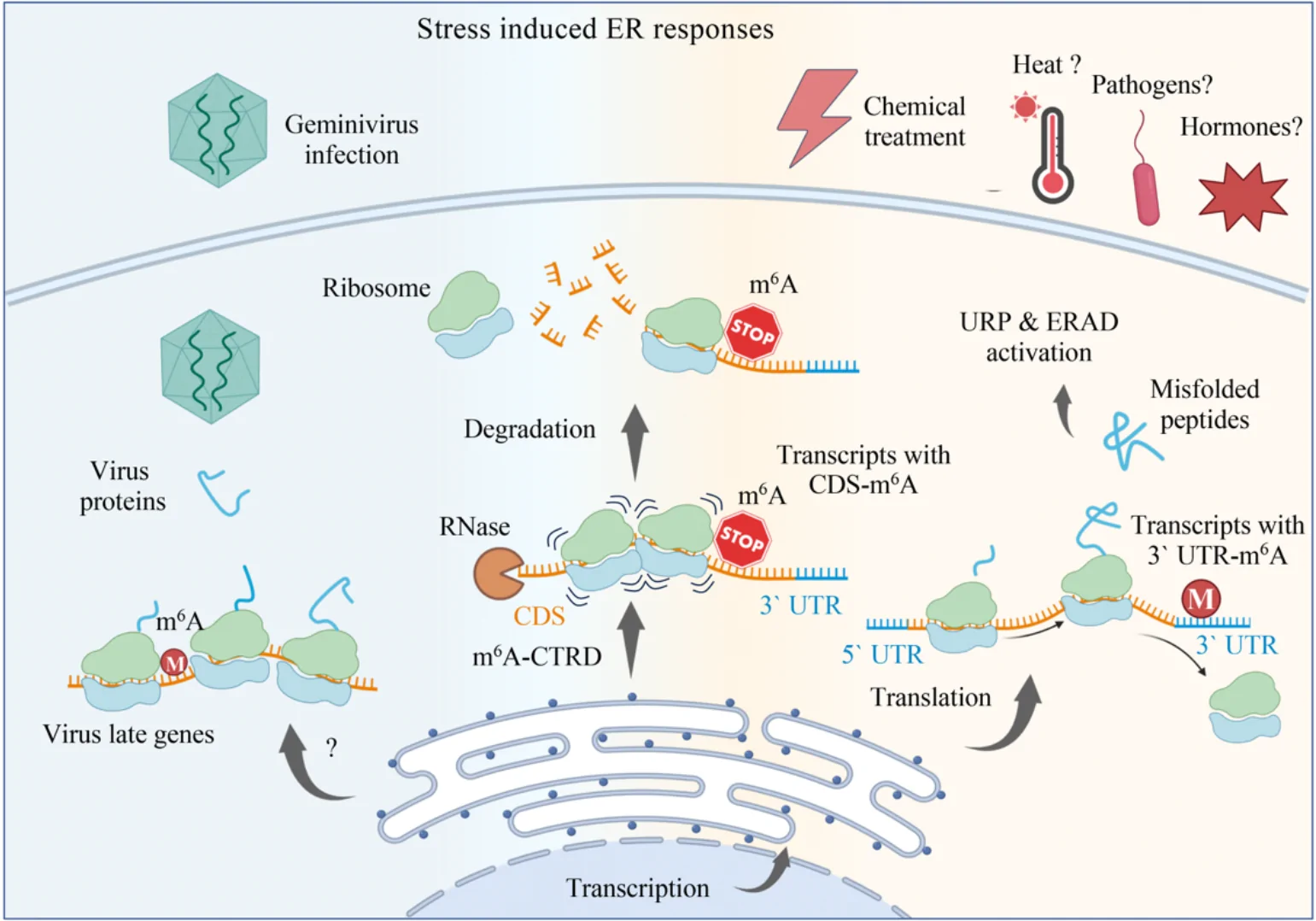
Proposed model for CDS-localized m^6^A-mediated RNA degradation under stress. Under stress conditions, CDS-m^6^A modifications promote rapid mRNA degradation on the ER, thereby alleviating the translational burden on ER-associated ribosomes and enabling plants to adapt to adverse conditions. Specifically, stress-induced upregulation of m^6^A modifications in the CDS facilitates targeted mRNA decay, which may in turn regulate the ER stress response pathway and contribute to the maintenance of cellular homeostasis. This model integrates the interplay between m^6^A modifications, ribosome co-localization, and mRNA degradation, highlighting their coordinate roles in modulating plant stress tolerance. Created with Biorender.com.

## Data Availability

Sequencing datasets are publicly accessible through the NCBI Gene Expression Omnibus (GEO) and European Nucleotide Archive (ENA) as follows: RNA-seq datasets for heat stress (7-day-old seedlings) under PRJNA485260[^52^] and PRJEB20305[^53^]; treatment with ABA (4-week-old plants) under PRJNA472051[^54^]; treatment with SA (5-week-old plants) under PRJNA224133[^55^]; treatment with Flgin 22 (14-day-old seedlings) under PRJNA486344[^56^]; and treatment with Pst AvrRps4 (4-week-old plants) under PRJNA477936[^57^]. MeRIP-seq data from 3-week-old plants are available under PRJNA1102430[^59^]. Alpha index and ribosome TRAP-seq data from 5-day-old seedlings are deposited under PRJNA561620[^63^]. Degradome-seq data from 3-week-old plants are accessible under PRJNA1092576. GUMCT-seq data from floral samples are under PRJNA203678 (control) and PRJNA292441 (treatment with CHX)^49,71^. PARE-seq data from 10-day-old seedlings are under PRJNA484887[^68^]. Raw data for RNA half-life analysis from 5-day-old seedlings are available at ArrayExpress under accession E-ATMX-21[^87^].
