# Supplementary material for: CDS-localized m6A drives co-translational RNA decay to relieve biotic and abiotic endoplasmic reticulum stresses
Source: Nat Plants. Author manuscript; Available in PMC 2026 Oct 29. (PMC13717571; doi:10.1038/s41477-026-02299-4)

Fig. 1b

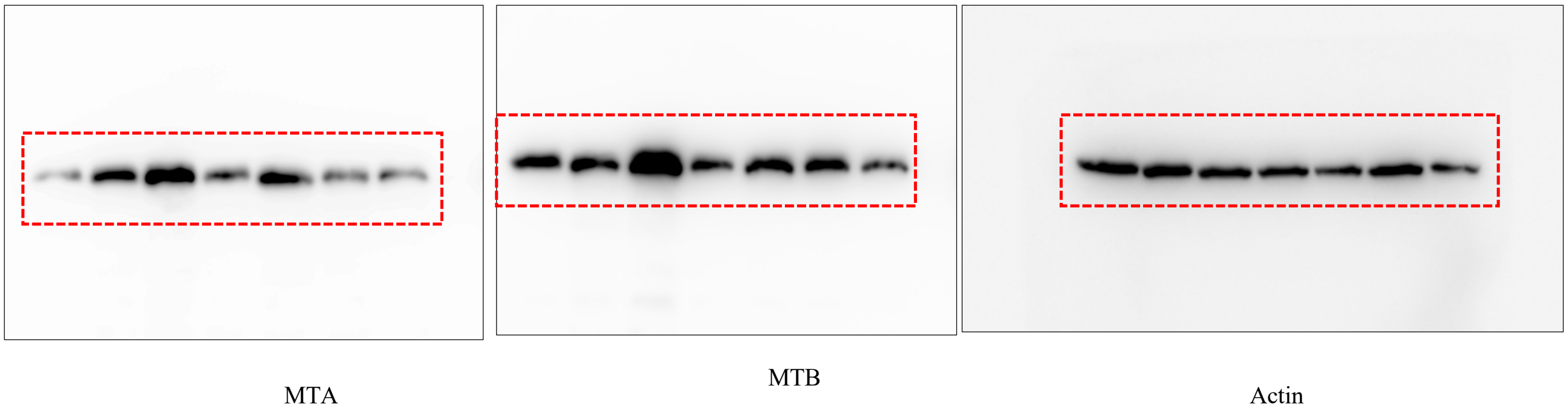

Fig. 4b

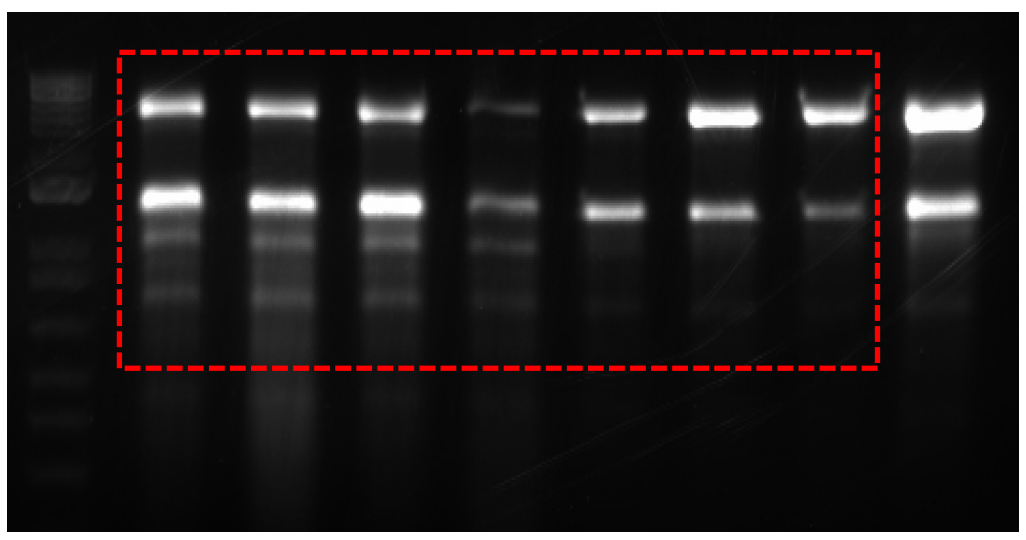

Fig. 4c

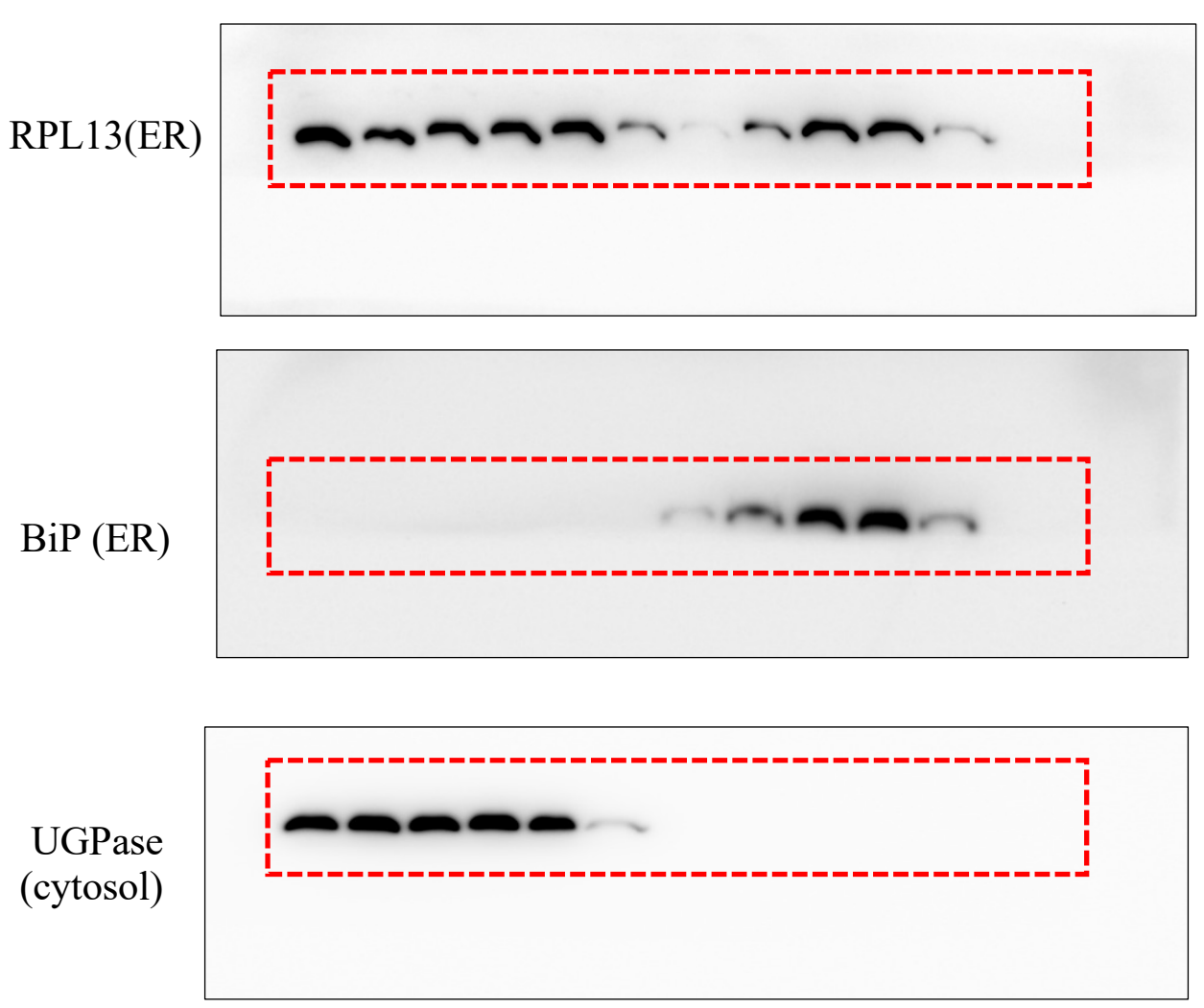

Fig. 4d

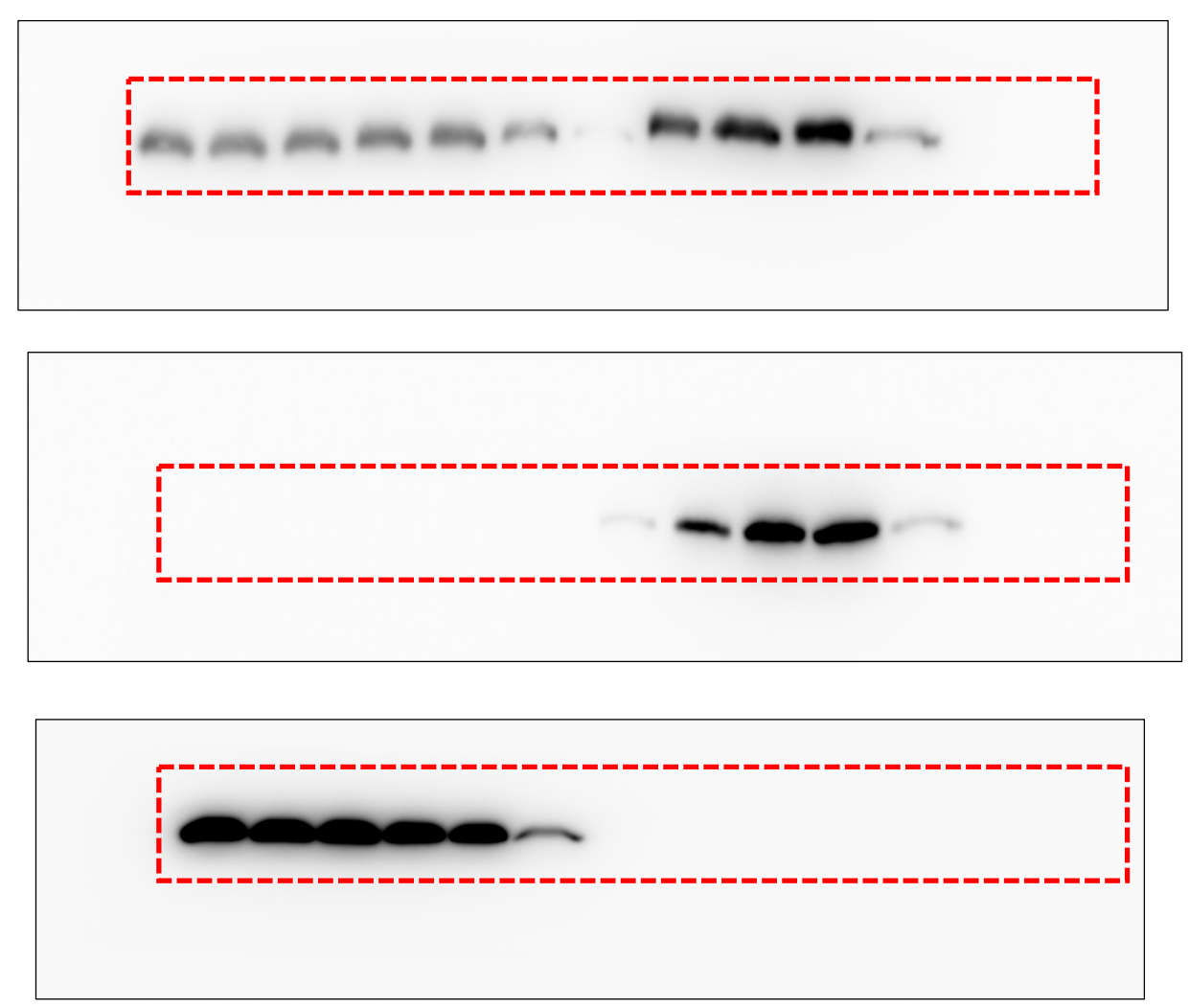

Fig. 4e

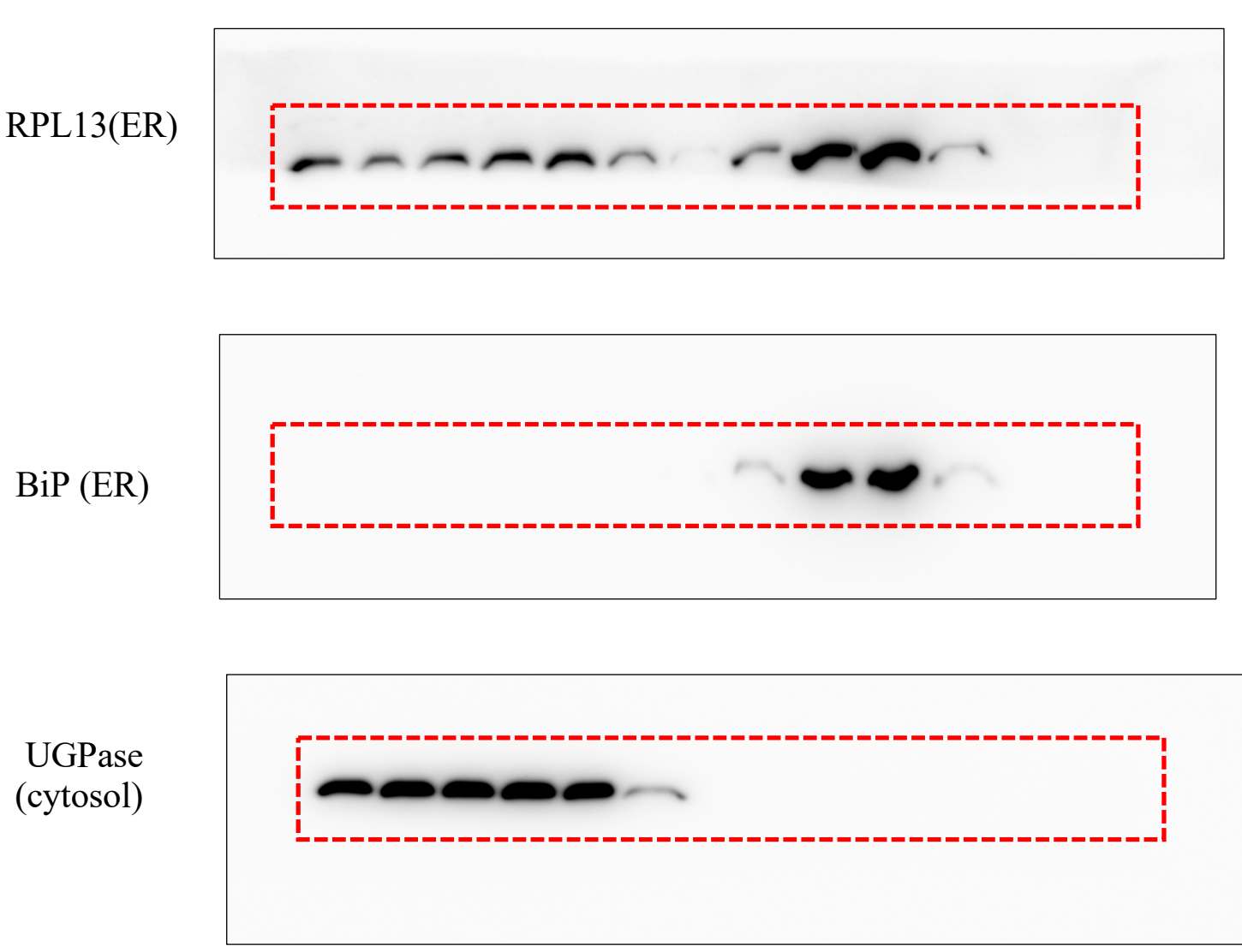

Fig. 4f

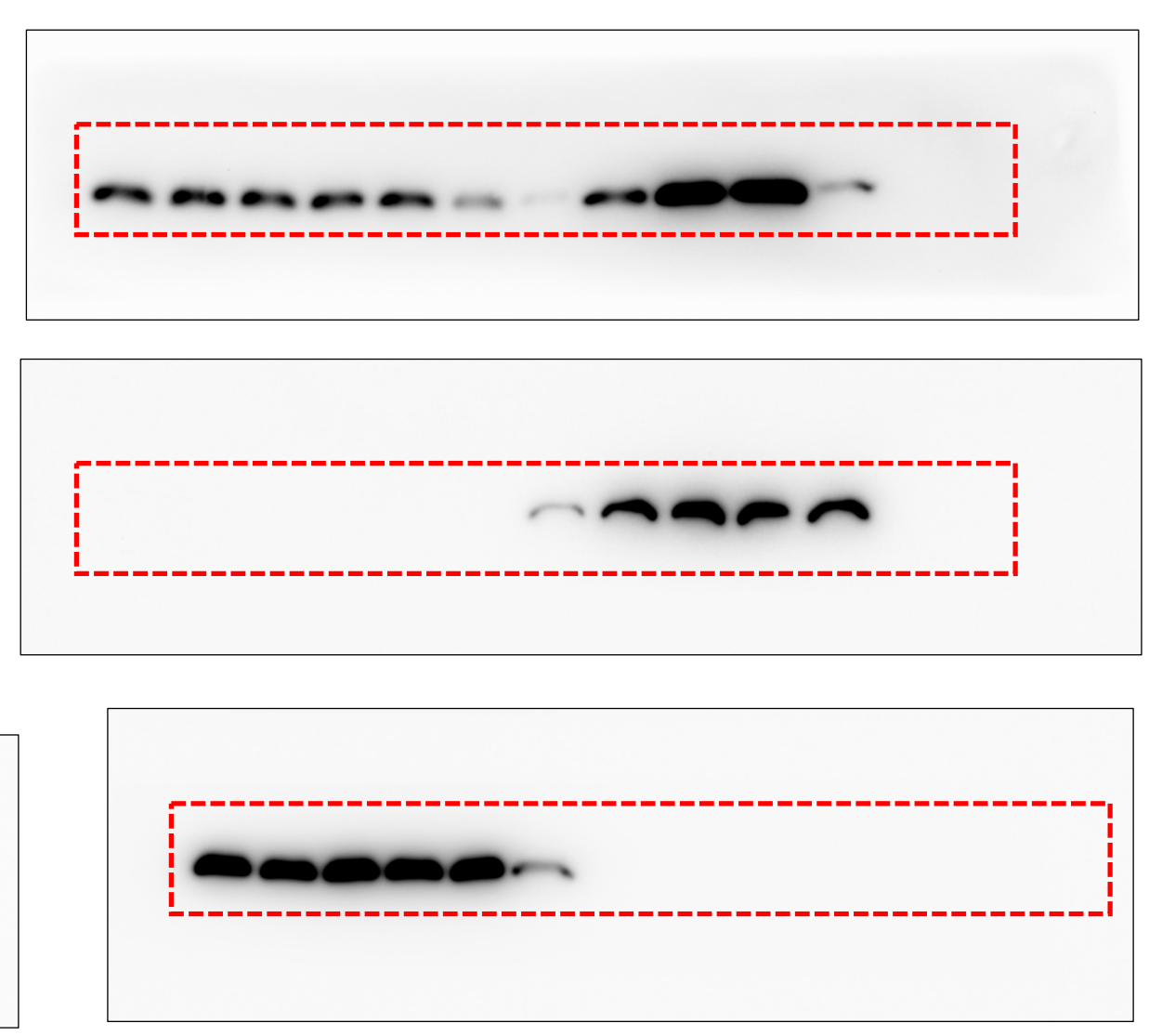

Fig. 4p

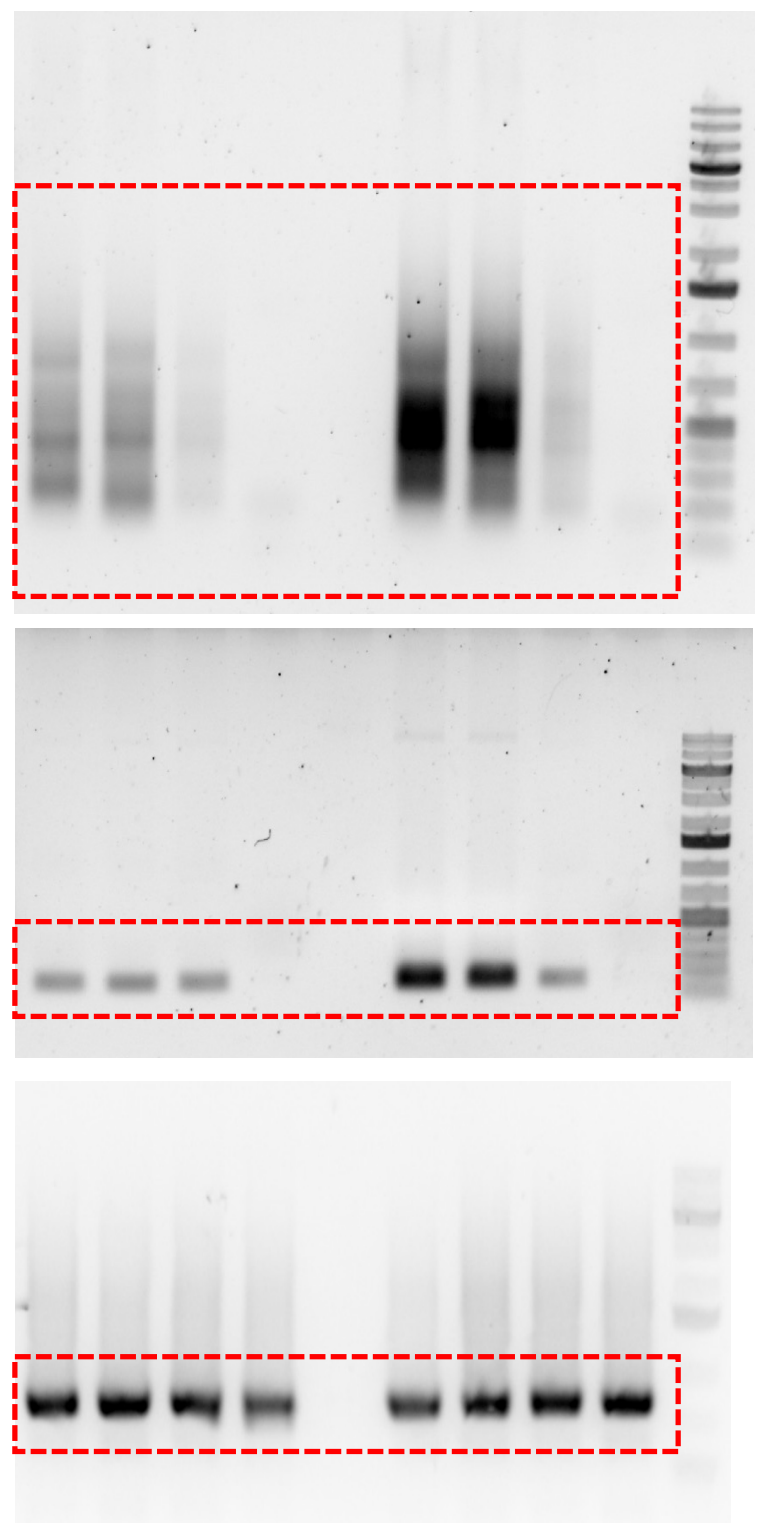

Fig. 4q

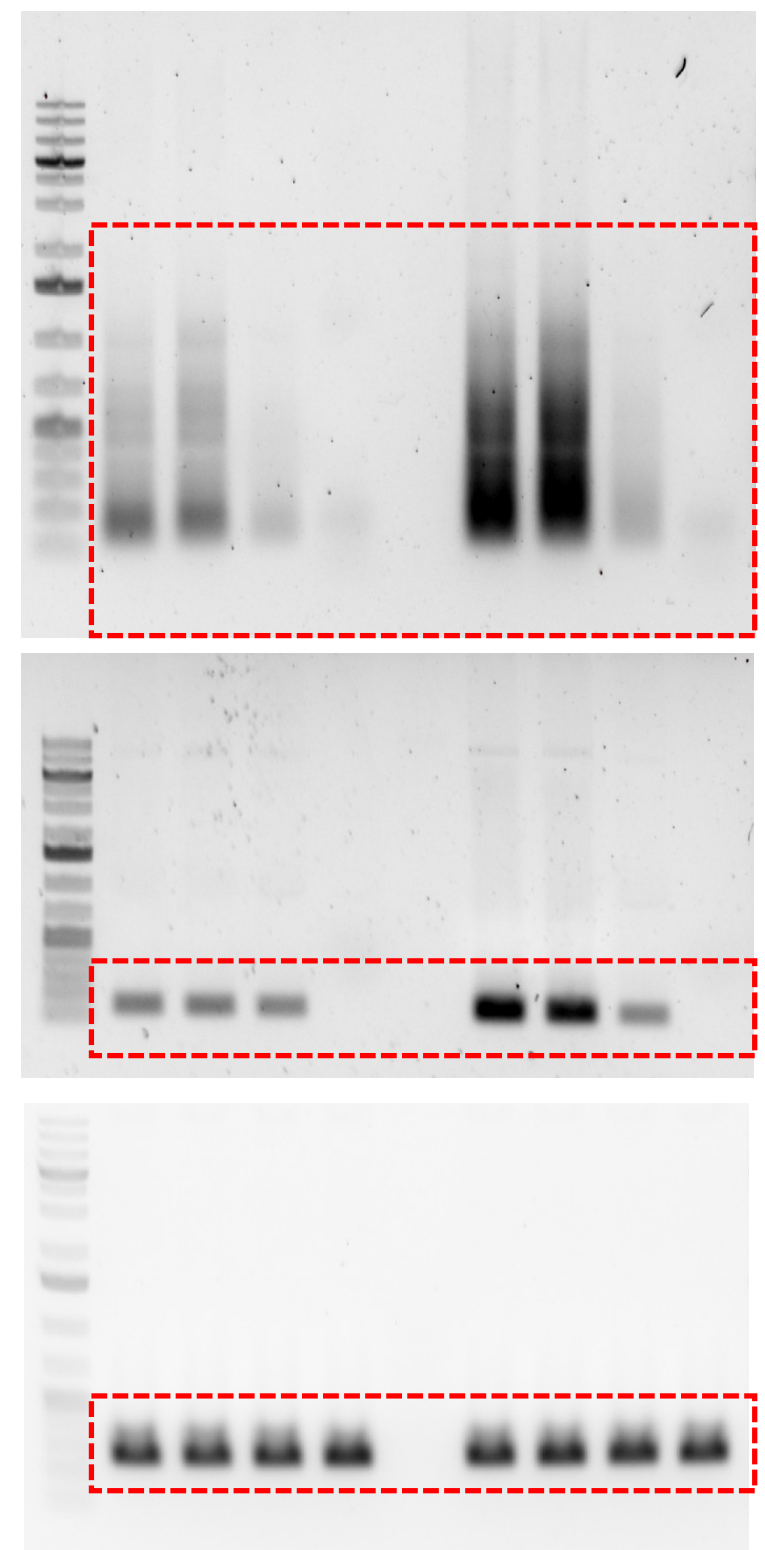

Fig. 5c and Extended Data Fig. 10f

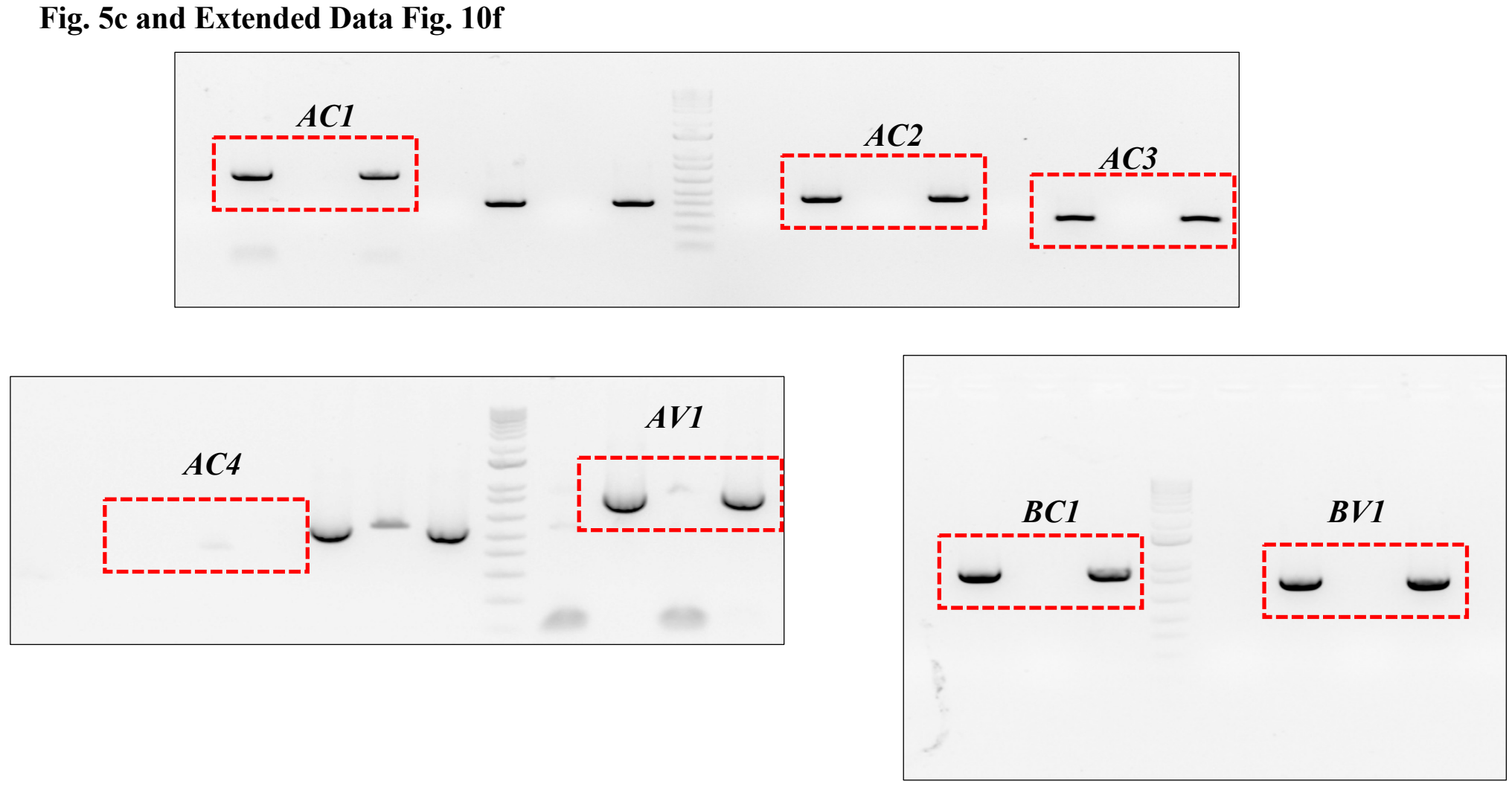

Fig. 5f and Extended Data Fig. 10g

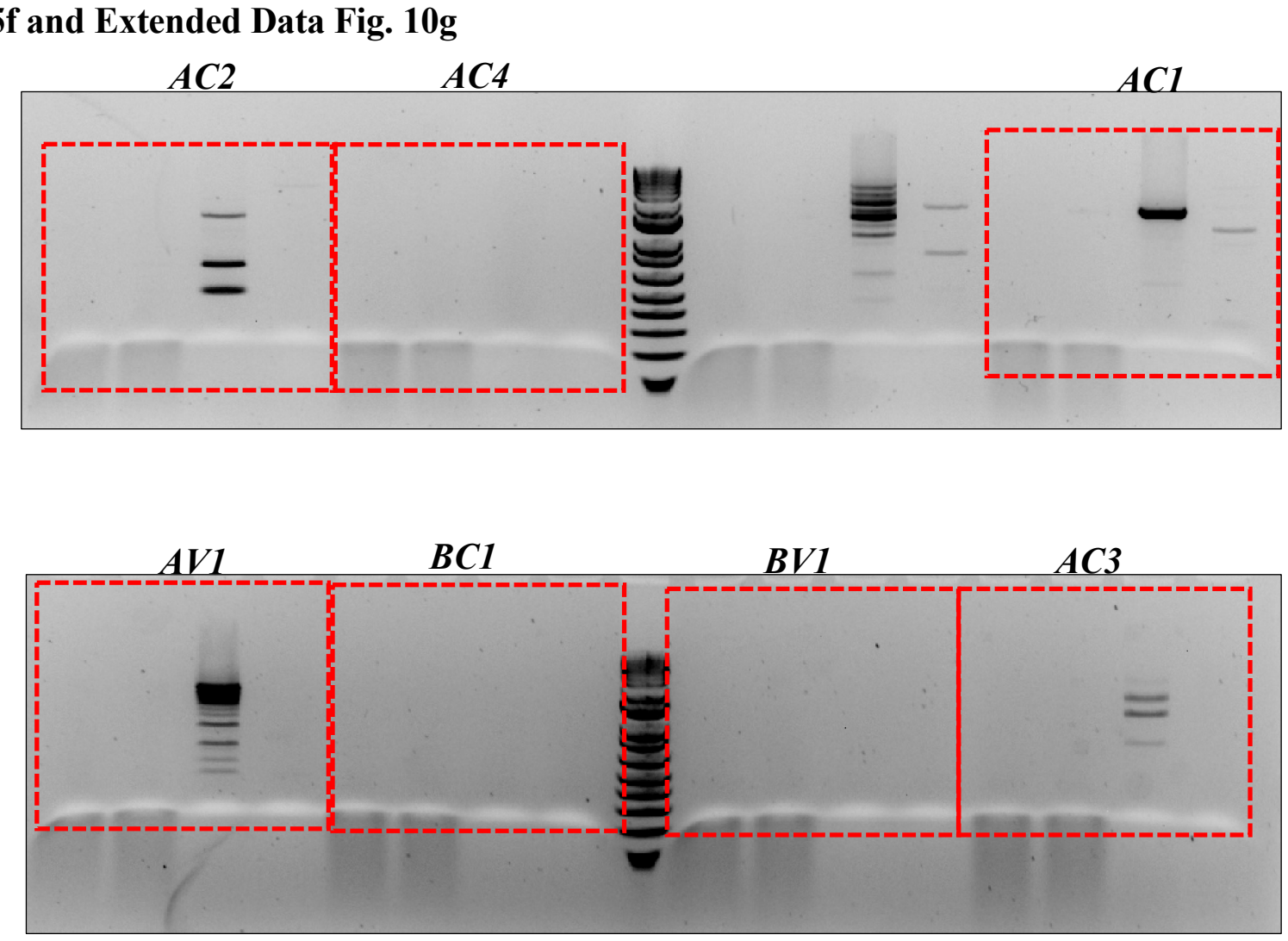

Extended Data Fig. 2a

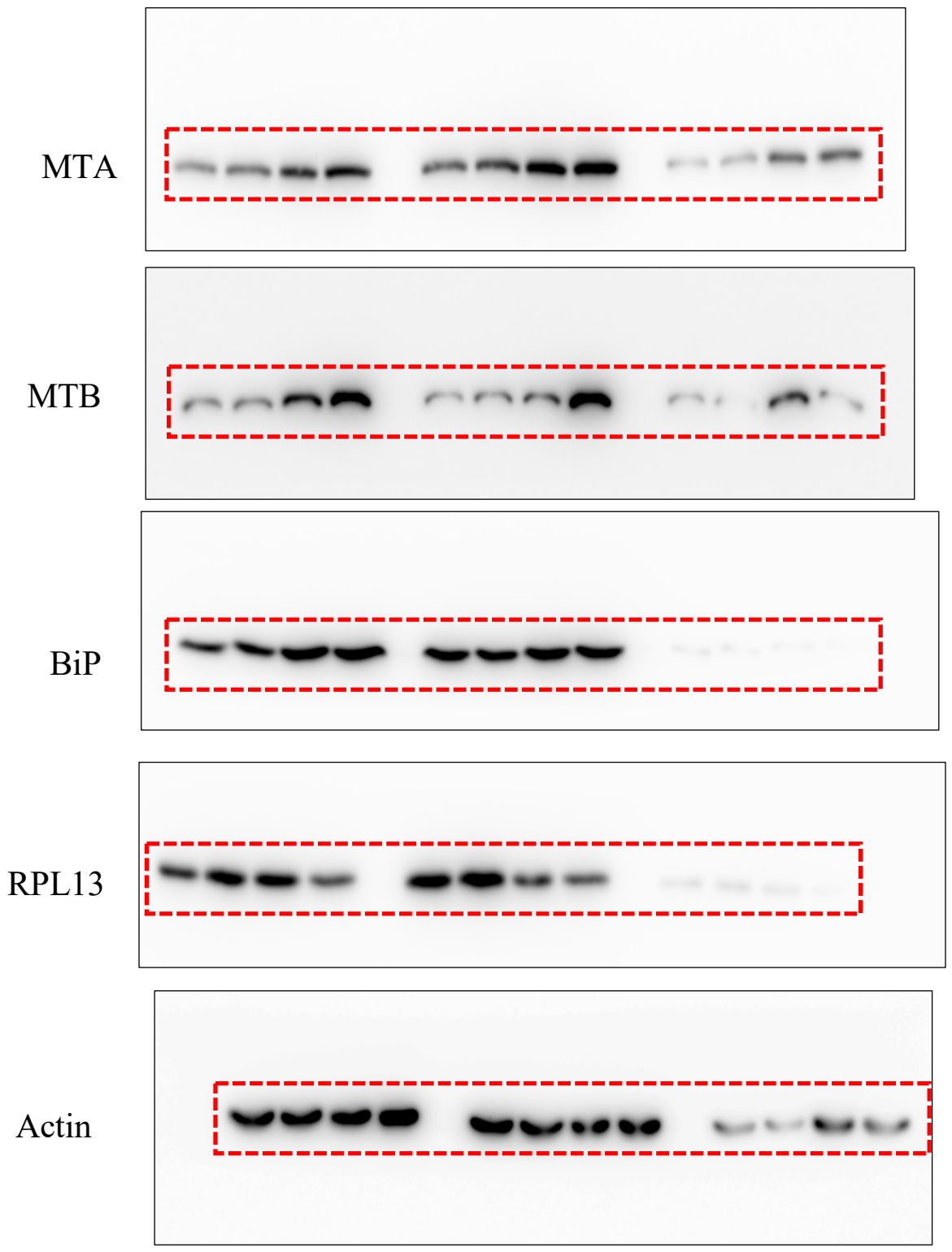

Extended Data Fig. 3c

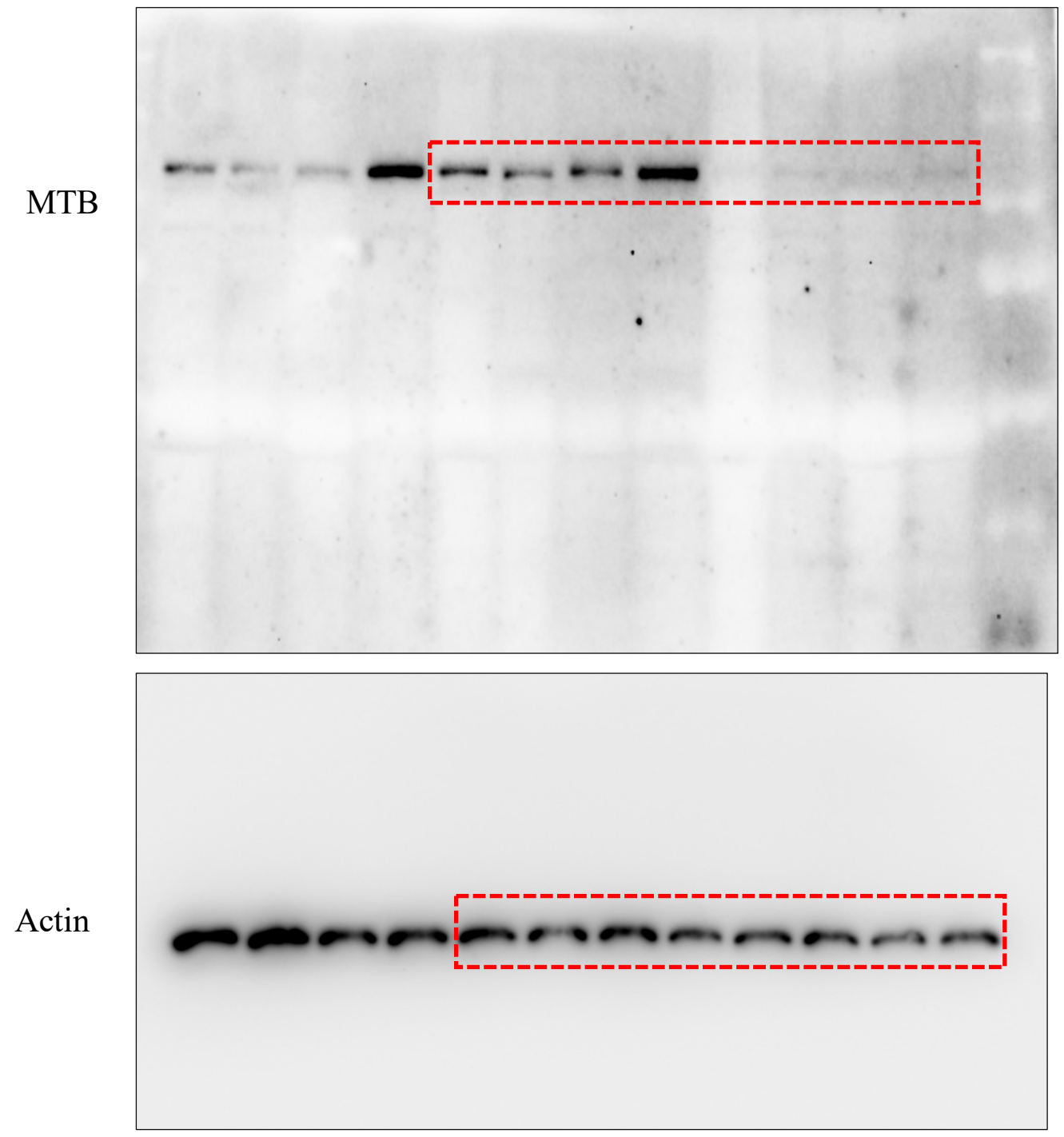

Extended Data Fig. 8a

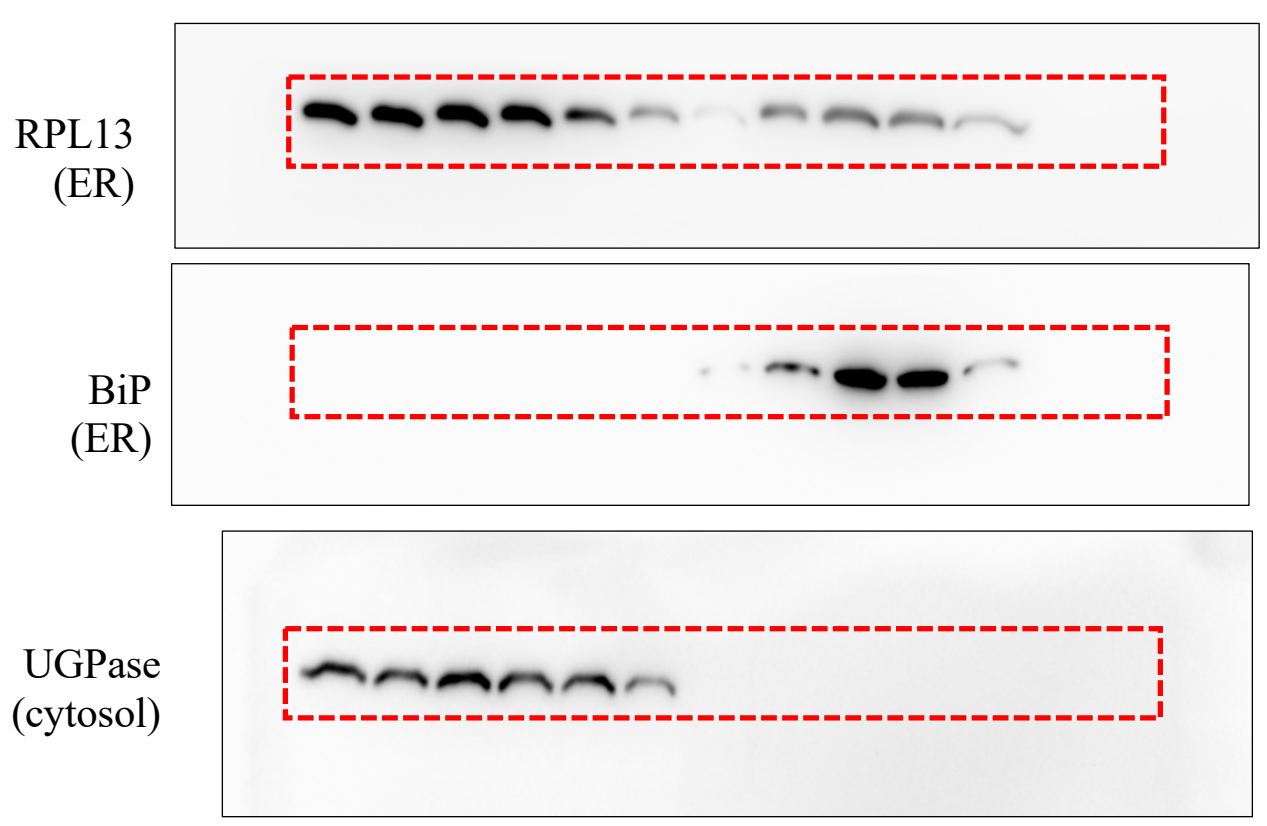

Extended Data Fig. 8b

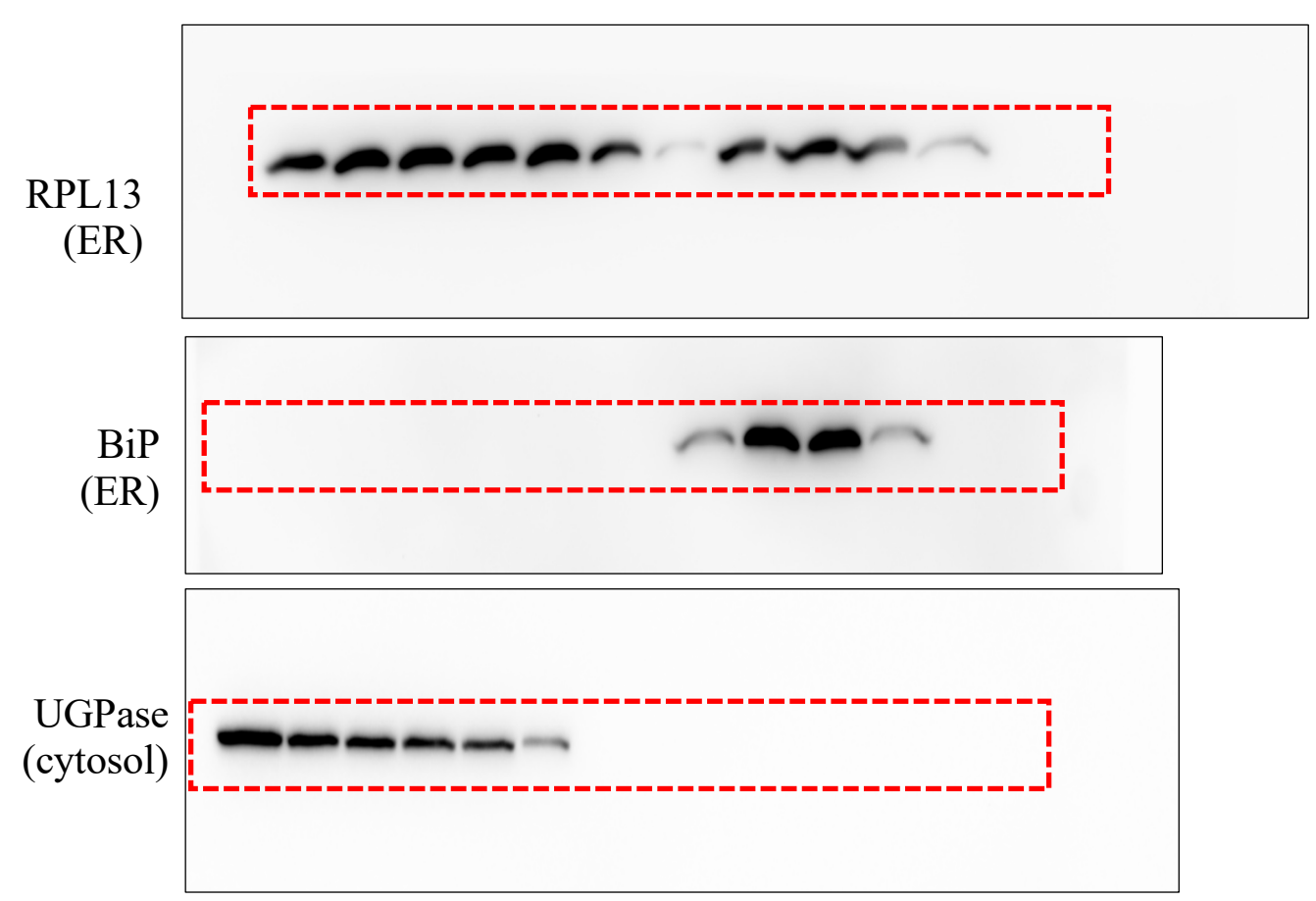

Extended Data Fig. 8c

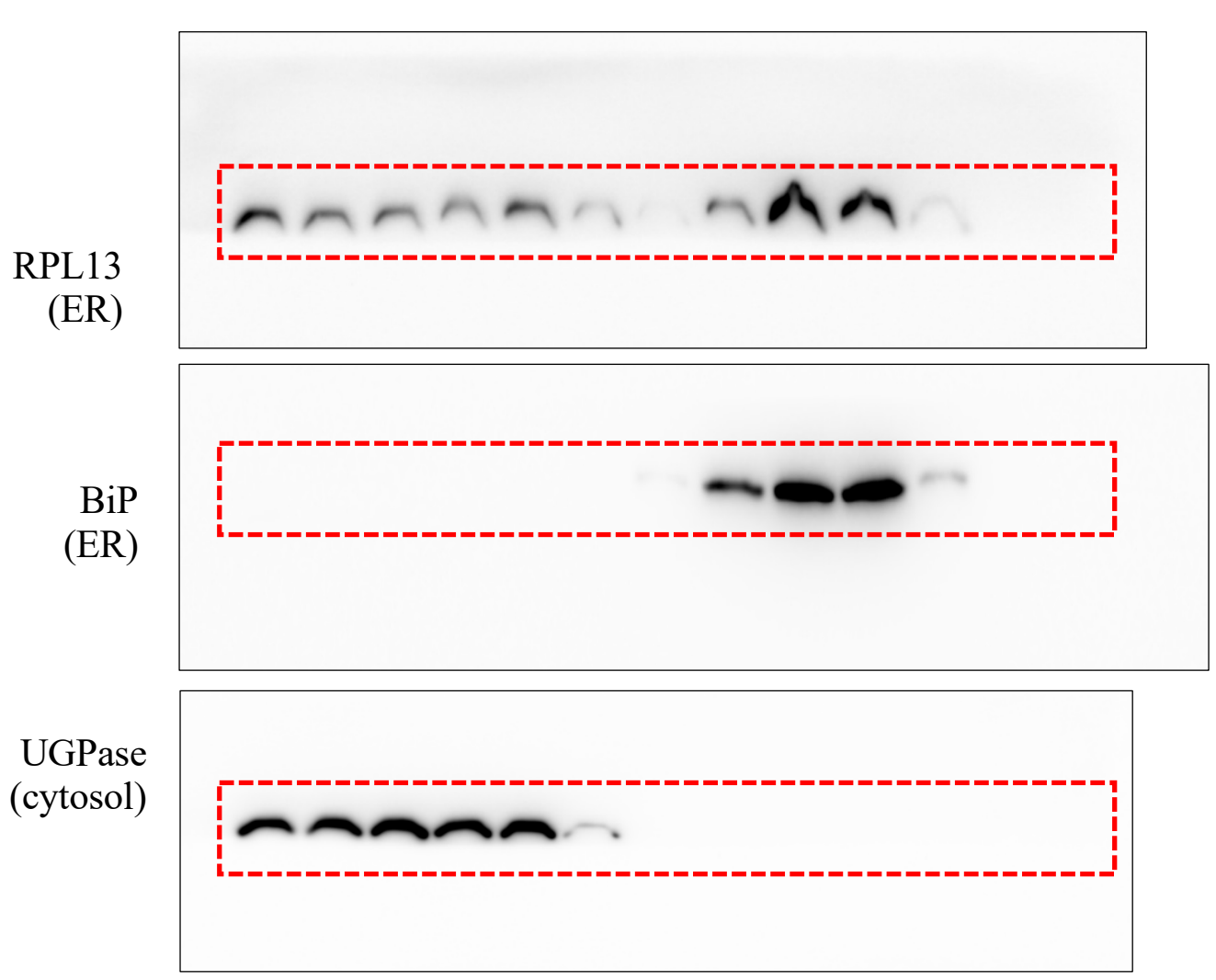

Extended Data Fig. 8d

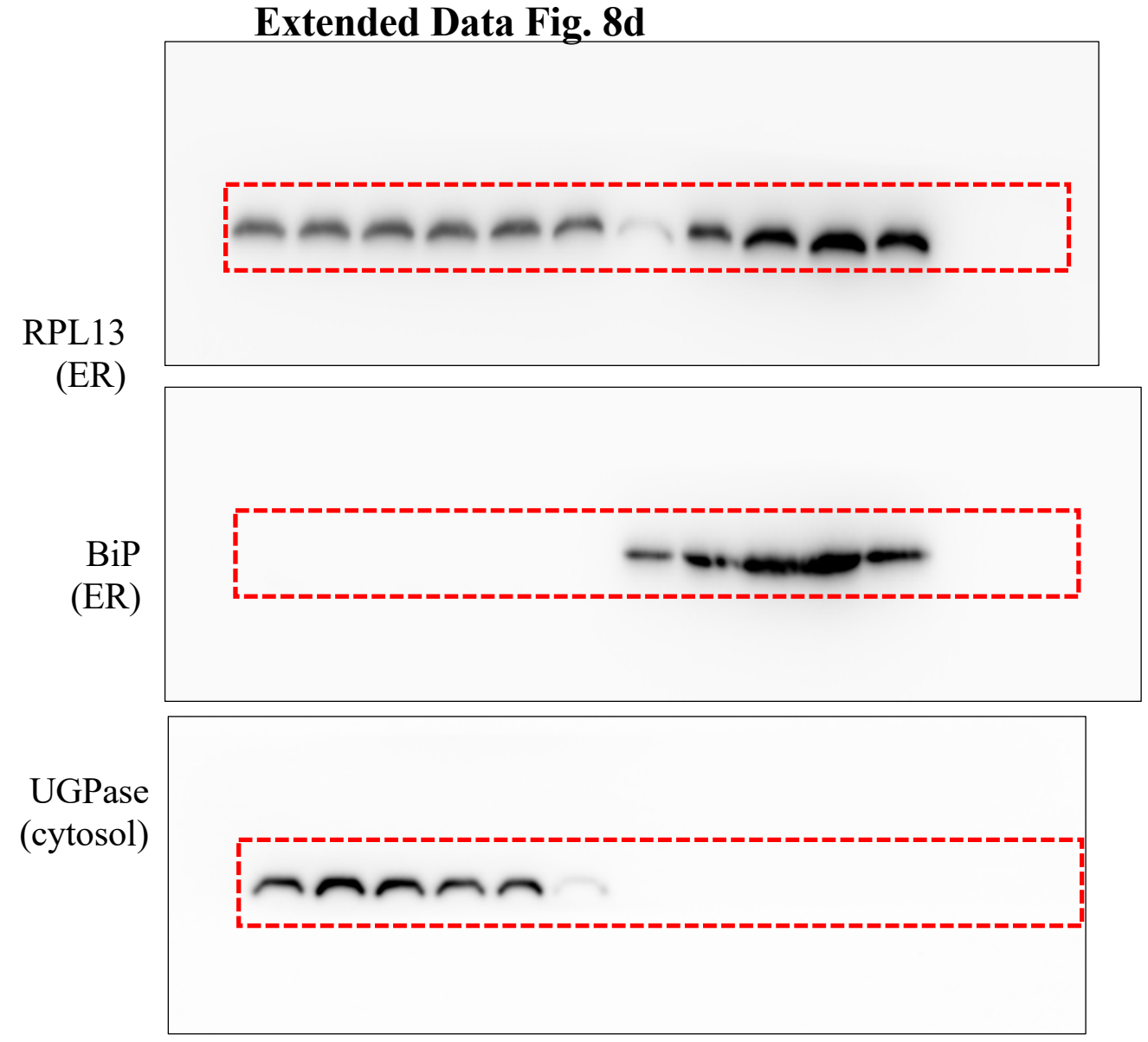

Extended Data Fig. 8e

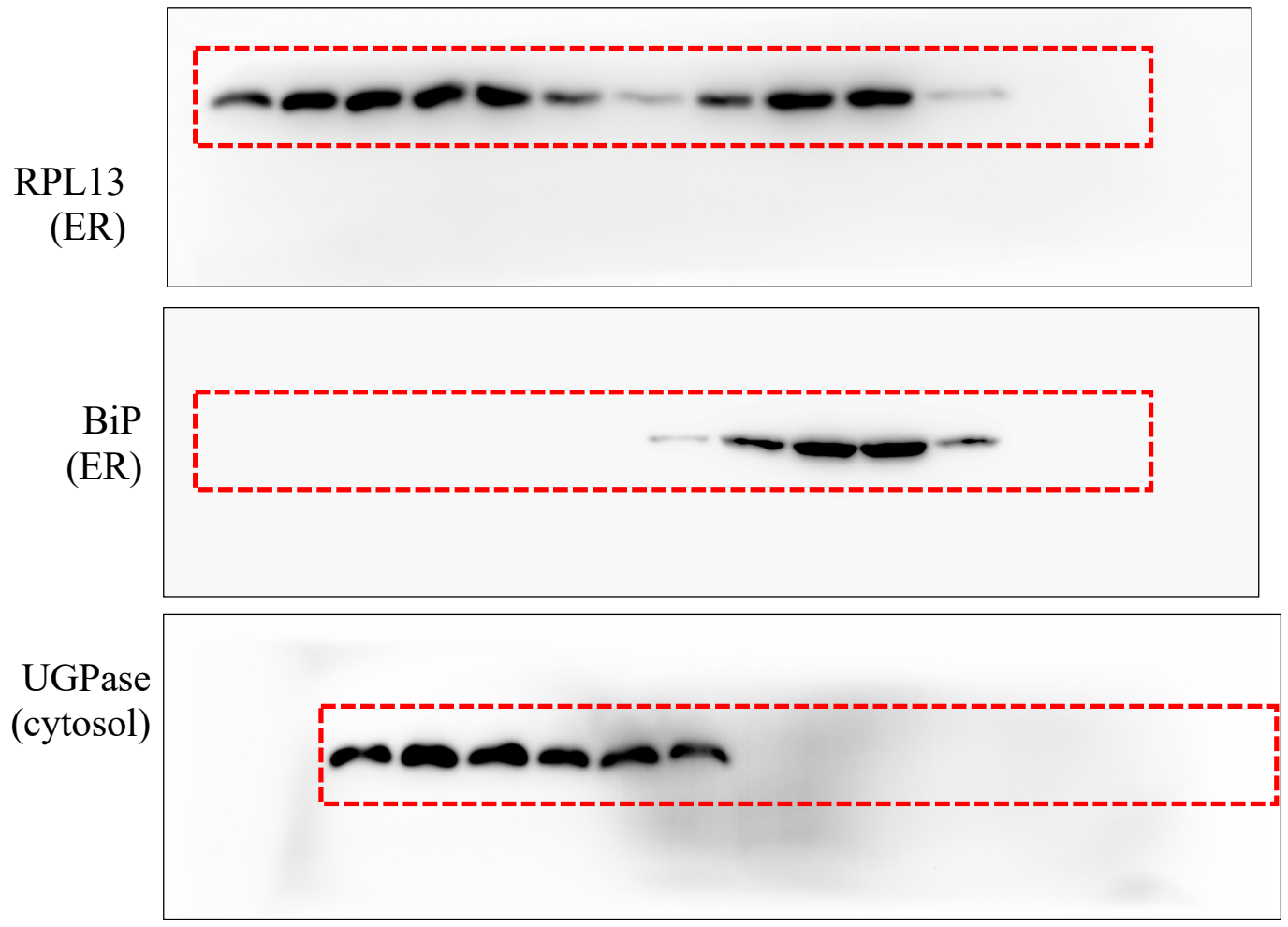

Extended Data Fig. 8f

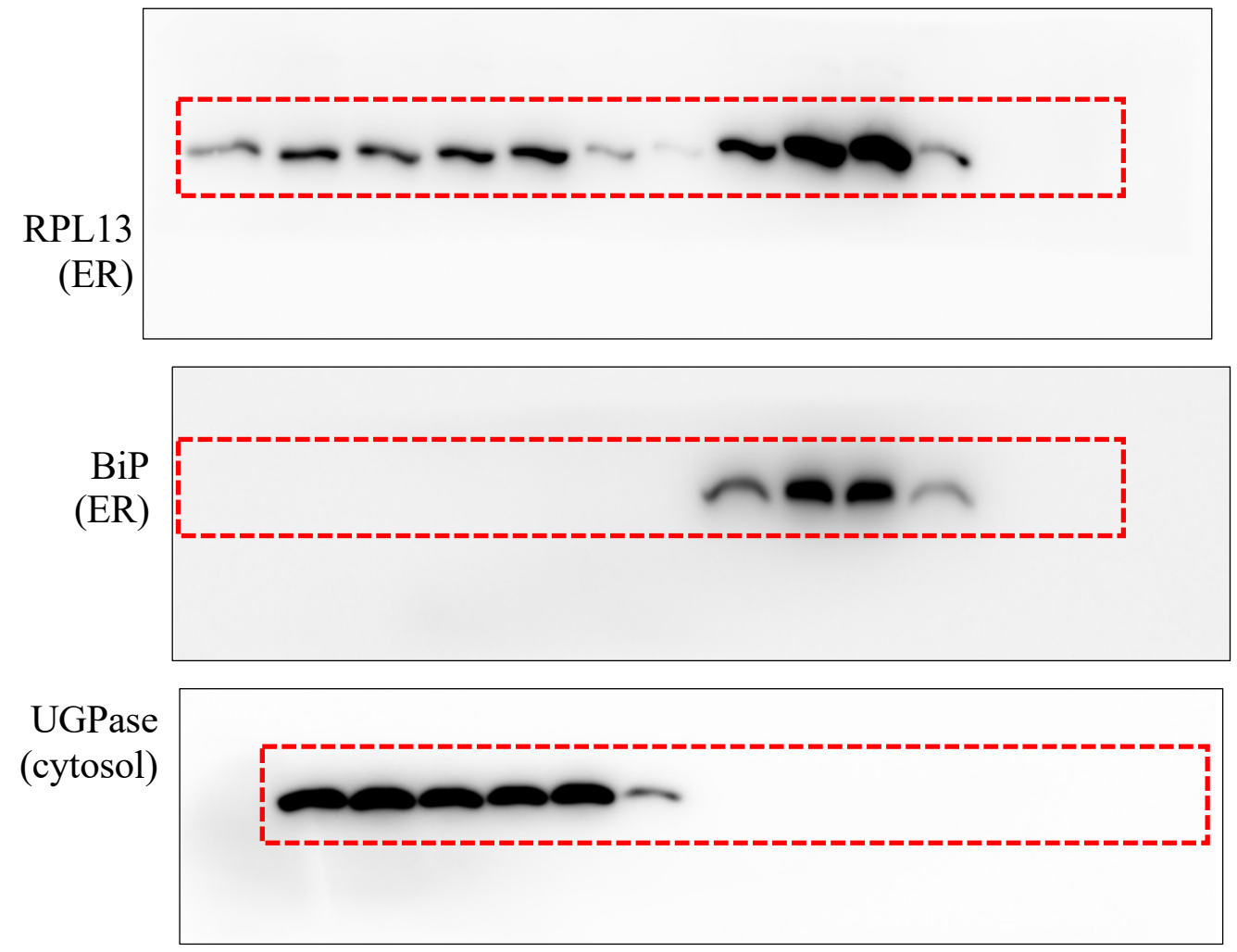

Extended Data Fig. 8g

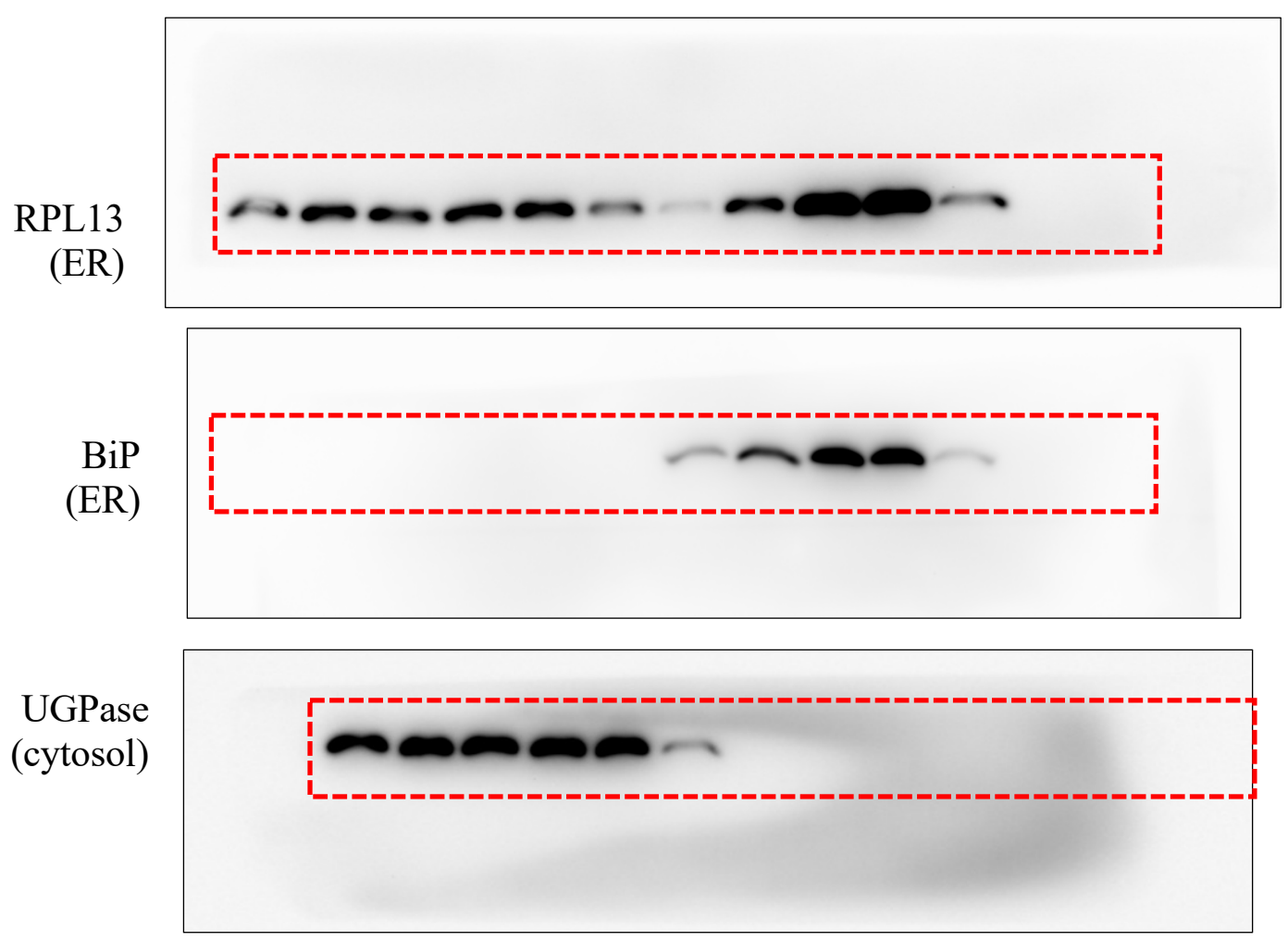

Extended Data Fig. 8h

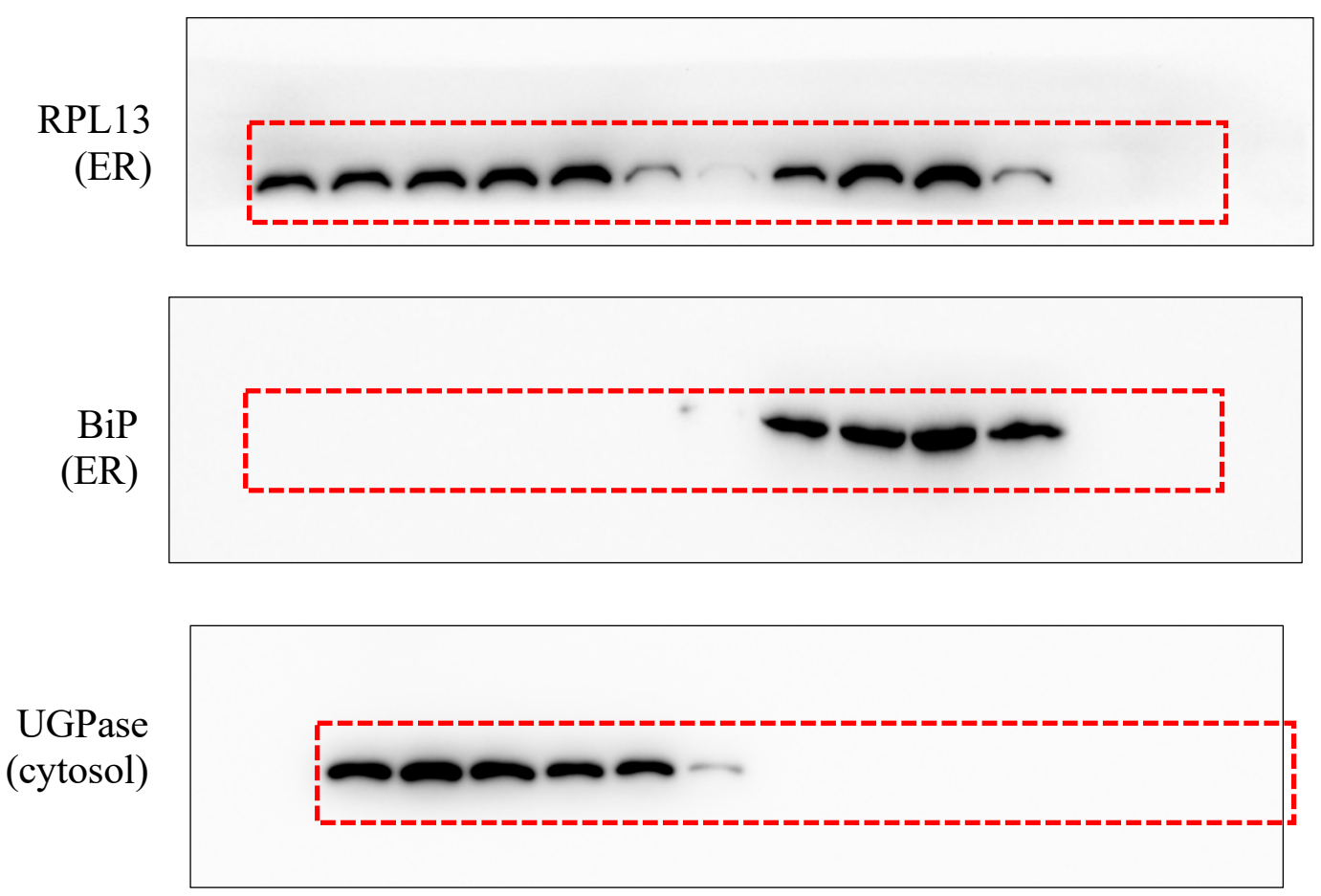

Extended Data Fig. 10d

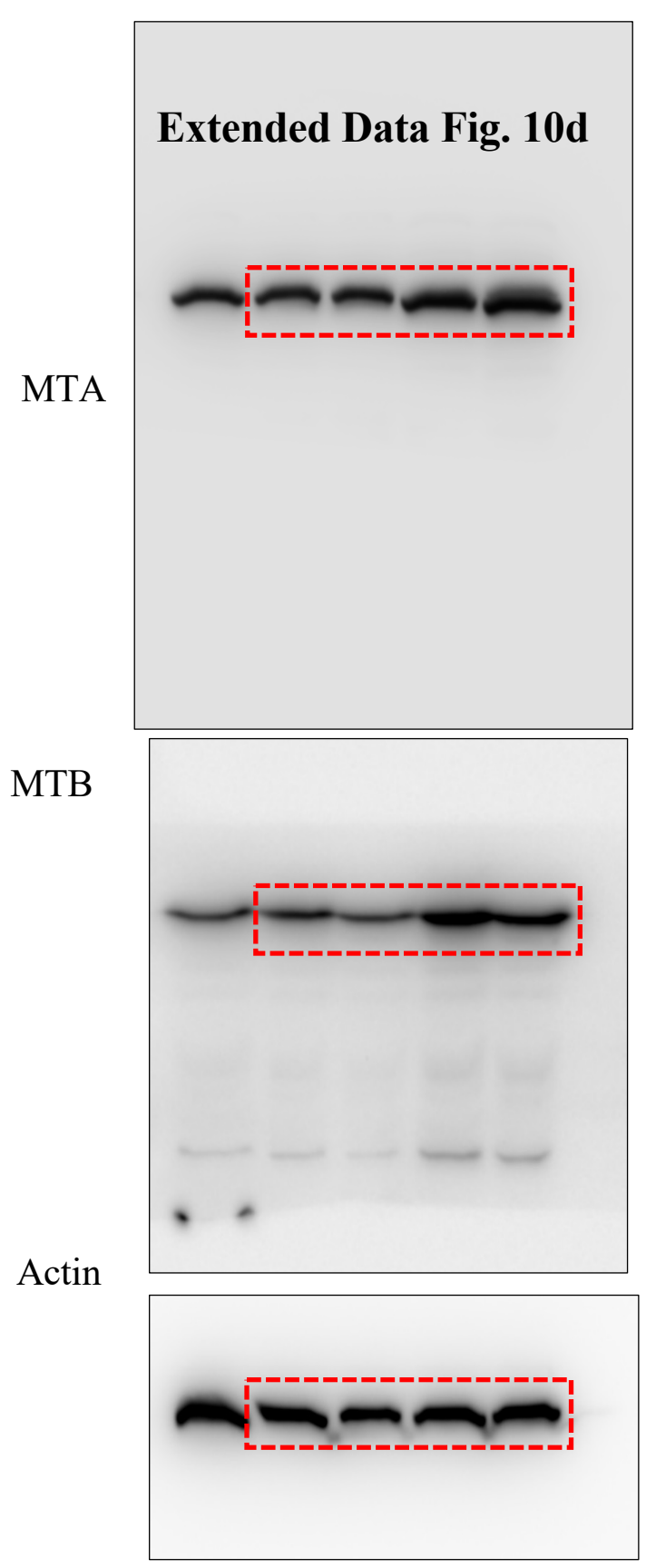

Extended Data Fig. 10h

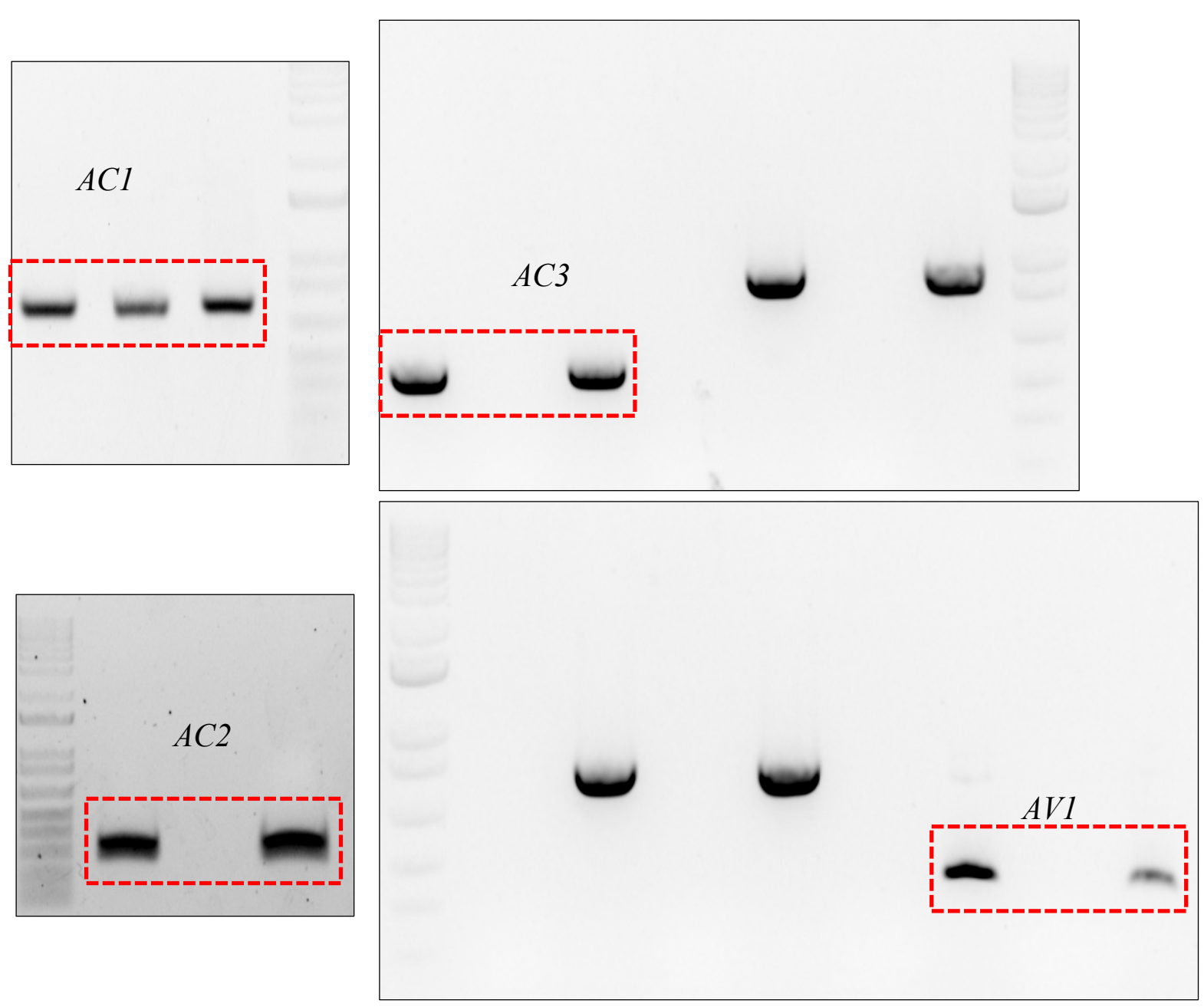

Extended Data Fig. 10i

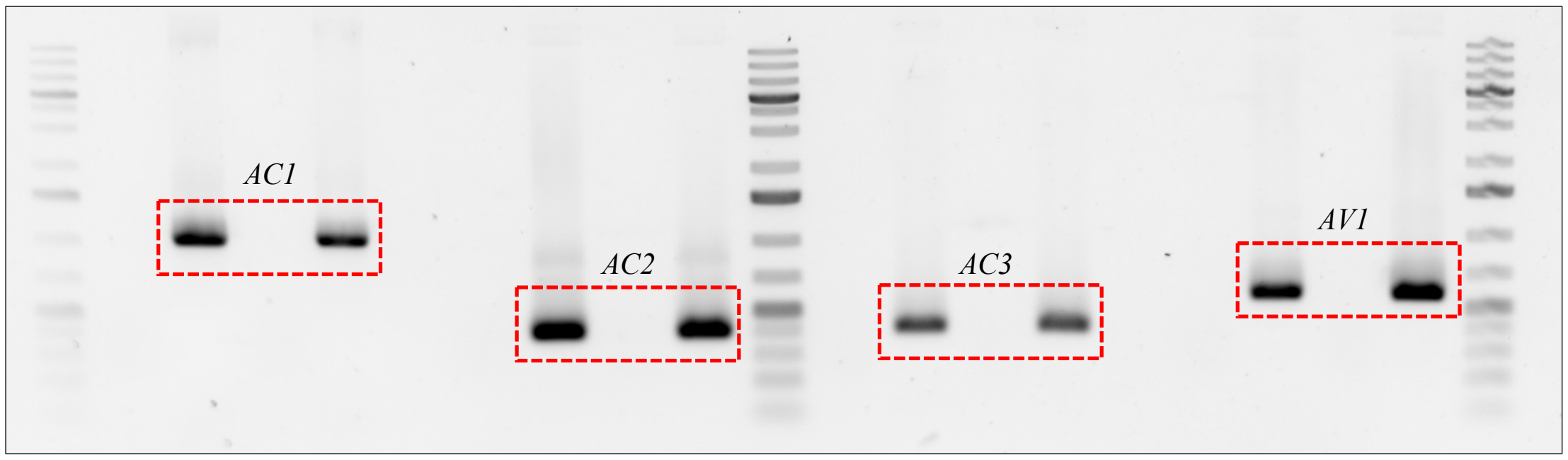

Extended Data Fig. 10j

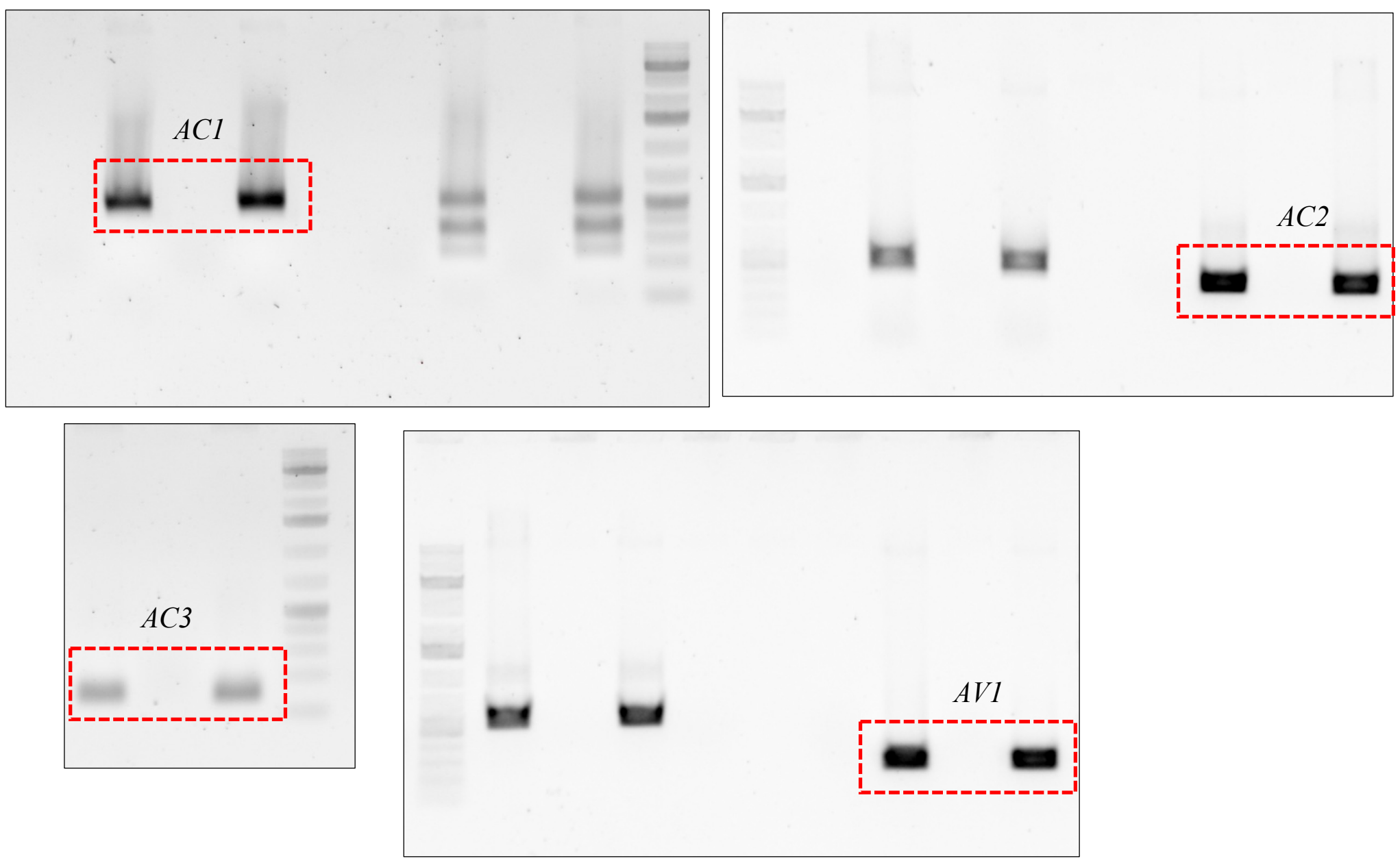

Supplement: Supplementary file 1 [file NIHMS2215862-supplement-Source_Figs.pdf]
